# DNAJA2 inhibits Newcastle disease virus replication by targeting its V protein to modulate the MDA5-MAVS pathway

**DOI:** 10.1186/s12866-025-04618-9

**Published:** 2025-12-22

**Authors:** Jiacong Mo, Fan Zhang, Yu Jiang, Mingrong Li, Shuting Wu, Ruiai Chen

**Affiliations:** 1 College of Veterinary Medicine, South China Agricultural University, Guangzhou, 510642 P. R. China; 2 Zhaoqing Branch Centre of Guangdong Laboratory for Lingnan Modern Agricultural Science and Technology, Zhaoqing, 526238 P. R. China

**Keywords:** Newcastle disease virus, MDA5, MAVS, IFN-β, DnaJ heat shock protein family member A2

## Abstract

**Background:**

As a molecular chaperone of heat shock protein 70 (HSP70), DnaJ heat shock protein family member A2 (DNAJA2) can facilitate protein folding under stress conditions and has recently emerged as a novel and critical regulator that modulates the innate immune response. The V protein of Newcastle disease virus (NDV) is a crucial virulence factor; however, its relationship with the heat shock protein family remains poorly understood.

**Results:**

We identified the interaction between DNAJA2 and NDV V proteins through immunoprecipitation‒mass spectrometry (IP‒MS) and confirmed that this interaction occurred mainly at residues 101–367 aa of DNAJA2. This finding was further validated by confocal microscopy and coimmunoprecipitation (co-IP). We found that the expression level of DNAJA2 is elevated during NDV infection. The overexpression of DNAJA2 enhances the expression of melanoma differentiation-associated gene 5 (MDA5) and mitochondrial antiviral signaling protein (MAVS), which upregulate the production of interferon-stimulated genes (ISGs) and interferon-β (IFN-β), thereby inhibiting the replication of NDV. In contrast, DNAJA2 knockdown promoted viral replication.

**Conclusions:**

Notably, DNAJA2-mediated modulation of innate immunity occurred specifically in the presence of NDV or NDV V proteins, suggesting that DNAJA2 potentially activates immune signaling pathways by targeting the NDV V protein. These findings establish the role of DNAJA2 against NDV and combine the heat shock protein family with the innate immune pathway, providing new institutional insights into the relationship between the virus and the host and the prevention and control of the virus.

**Supplementary Information:**

The online version contains supplementary material available at 10.1186/s12866-025-04618-9.

## Background

Newcastle disease (ND) is a highly contagious viral disease caused by Newcastle disease virus (NDV) and is currently causing ongoing harm to the poultry industry worldwide. Different NDV strains exhibit significant differences in tissue tropism and clinical symptom severity, with lesions primarily observed in the nervous, digestive, respiratory, and reproductive systems [1]. Highly infectious and economically detrimental viral diseases are often caused by lethal NDV [2].

NDV belongs to the Paramyxovirus genus [3]. Compared with a solitary negative-sense RNA strand, the NDV genome is responsible for the production of eight proteins [4]. The NDV V protein consists mainly of 239 amino acids, whose molecular weight is 36 kDa. The ZNF domain in the NDV V protein may block the interferon signaling pathway through multiple pathways [5]. Numerous studies have demonstrated that the V protein of NDV is a negative regulator of immunity. For example, it can directly inhibit interferon secretion [6]. Additionally, it can degrade mitochondrial antiviral signaling protein (MAVS) to inhibit type I interferon synthesis during the ubiquitination process [7]. Additionally, the V protein promotes viral proliferation by activating the MEK/ERK pathway [8]. Consequently, the NDV V protein is crucial in the virus lifecycle.

Heat shock protein family members play various roles in viral infections. For example, they support viral entry into host cells by forming complexes on the cell surface [9]. Although DnaJ heat shock protein family member A2 (DNAJA2) belongs to the heat shock protein family, the functions of DNAJA2 are multifaceted and diverse. As an example, it has the ability to control its engagement with unfolded substrate proteins and heat shock protein 70 (HSP70) [10]. Recent studies have demonstrated that it can degrade CSB proteins via chaperone-mediated autophagy, which can regulate transcription-coupled repair [11]. With respect to immune signaling pathways, aberrant mitosis resulting from defects in DNAJA2 has been shown to activate the cGAS-STING signaling pathway [12]. DNAJA2 seldom appears in reference to viral infections and is recognized solely as a crucial host element necessary for Japanese encephalitis virus (JEV) infection [13]. However, the involvement of DNAJA2 in the replication of NDV is still unclear.

Understanding the relationship between the heat shock protein family and the NDV virus is crucial for its prevention and control. The V protein of NDV is associated primarily with immune evasion. Identifying the host proteins that interact with the V protein can lead to new approaches for preventing and treating viruses and developing drugs. These results indicate that DNAJA2 not only plays a key role in host restriction during NDV infection but also reveals a unique mechanism by which DNAJA2 combats NDV infection.

## Materials and methods

### Cell culture and viruses

Dulbecco’s modified Eagle’s medium (DMEM; Invitrogen, USA), supplemented with 10% fetal bovine serum (FBS; Gibco), 2 mM L-glutamine, and 1% penicillin‒streptomycin, was used to culture the chicken fibroblasts (DF-1). DF-1 cells were cultured at 37 °C with 5% carbon dioxide. The NDV strain DHN3, which was isolated and identified by our laboratory in 2015 [14], was amplified from DF-1 for use in this study. UMNSAH/DF-1 (Chicken Embryo Fibroblasts) Purchased from Pronasai’s official website in September 2023, Product Code: CL-0279.

### Plasmid constructs

The DNAJA2 gene was amplified in DF-1 via a library sequence (GenBank accession number: NM _ 001005841.2). The EGFP-N1 or pXJ40-MYC vectors were used to clone cDNA encoding full-length chicken DNAJA2 and mutants with DNAJA2 deletions. Every construct was verified through DNA sequencing analysis conducted by Sangon Biotech in Shanghai. We acquired the pCMV-Flag and pCMV-Flag-V vectors from our laboratory [15]. Each primer used in this research is displayed in Table 1.

**Table 1:** Primers used for construction of plasmids and qRT-PCR

Primers	Forward primer (5′−3′)	Reverse primer (5′−3′)
Pxj40-MYC-DNAJA2	GCCATGGAGGCCCGAATTCGGATGGCGAACGTGGCCGACAC	GCGGCCGCGGTACCTCGAGTTACTGATGGGCACACTGTACCC
pEGFP-DNAJA2(1–100)	GCGCTACCGGACTCAGATCTCATGGCGAACGTGGCC	TACCGTCGACTGCAGAATTCGCATGAAATTGAACAATCCACCACC
pEGFP-DNAJA2(101–367)	GCGCTACCGGACTCAGATCTCATGGGTGGTCAGAGTAGAAGTCG	TACCGTCGACTGCAGAATTCGATCACCAATTACATTAGGAAATTCTGGTCTAGC
pEGFP-DNAJA2(368–411)	GCGCTACCGGACTCAGATCTCATGGCAGAAGAGGTAGATCTTCAG	TACCGTCGACTGCAGAATTCGCTGATGGGCACACTGTACCC
pEGFP-DNAJA2	GCGCTACCGGACTCAGATCTCATGGCGAACGTGGCC	TACCGTCGACTGCAGAATTCGCTGATGGGCACACTGTACCC
QP-DNAJA2 (qRT-PCR)	TGTCCGTGTAGTCAGAGGT	CTGGGCTAATCCAGTTATT
QP-HN (qRT-PCR)	TCACAGGGACTGAAGAGGA	CCGTAAACTGGGAACCATA
QP-MDA5 (qRT-PCR)	GATGCCGCCAGAAGAGTAT	GGAATGTTATTAGTGAAGGGTT
QP-MAVS (qRT-PCR)	TGACTCAAACAAGGGAAGTAATG	AATCAGAGCGATGCCAACA
QP-IFNα (qRT-PCR)	CGCCAAAGCCTCCTCAACC	CAGGCGCAGGCGCTGTAAT
QP-IFNβ (qRT-PCR)	CACCACCACCTTCTCCTGC	TGGCTGCTTGCTTCTTGTC
QP-OASL (qRT-PCR)	CGCCCTGCTAAGCTGAAAGACCT	TGTGCCCTCCTCCCAGGCGTA
QP-IFI127 (qRT-PCR)	ACACTCCTCAGGCTTTACCAG	CTCCTTTGCCACCCATTGAGA
QP-ISG15 (qRT-PCR)	CTTGTGCAGCATCTGAAGTCC	ATCTGCACGTCCTTCTTACGG
QP-MX1 (qRT-PCR)	GTCATTACTCGCTGTCCTC	TCTTGGGCTTTTCTTATTGCT

### RNA extraction and real-time quantitative PCR

Complete cellular RNA was extracted from NDV-infected DF-1 cells via the TRIzol reagent (TransGen, China). The Evo M-MLV RT Mix Kit (Accurate Biology, China) was subsequently used to reverse transcribe 1 µg of total RNA to generate cDNA. The genomic copy number of the target gene was quantified via relative quantitative real-time PCR with SYBR Green real-time PCR premix (Accurate Biology, China) and specific primer pairs (Table 1) on the Archimed Analyzer system (Applied Accurate Biology). β-actin served as the internal benchmark for comparison.

### RNA interference

Small interfering RNA (siRNA) targeting DNAJA2 (siDNAJA2: 5′-CGGAGAAACGTGAGTTATA-3′) was generated by Sangon (Shanghai) to inhibit the endogenous expression of the DNAJA2 protein. Concurrently, a plasmid was created to express the negative control siRNA (**siNC**). DF1 cells were transfected in 12-well plates with siDNAJA2 or siNC for 24 h. The effectiveness of siDNAJA2 in targeting DNAJA2 was assessed via real-time quantitative PCR (qRT‒PCR) and Western blot (WB) tests.

### Western blot and coimmunoprecipitation assays

Six-well plates were used to seed DF-1 cells, which were subsequently transfected with the designated plasmids via the Lipofectamine 3000 reagent (Invitrogen, USA) for 36 h. The cells were then collected in NP-40 lysis buffer. A total of 40 µL was taken as the input sample, and anti-Flag or anti-Myc affinity beads (Sigma‒Aldrich, USA) were used to incubate the remaining lysis buffer. After washing with NP-40 buffer, the proteins were separated via SDS sample buffer. A 10% sodium dodecyl sulfate‒polyacrylamide gel electrophoresis (SDS‒PAGE) was used to separate the proteins, and a polyvinylidene fluoride (PVDF) membrane was used to transform the proteins (Roche, UK). The membrane was blocked with high-efficiency blocking buffer suitable for Western blotting and incubated with primary antibodies, including anti-MDA5, anti-MAVS, anti-IFI127, anti-ISG15, anti-TBK1, anti-IRF3, anti-P-IRF3, anti-Myc, anti-Flag, anti-DNAJA2, and anti-β-actin (Proteintech Group, China), as well as an anti-HN antibody from our laboratory (Han et al., 2025). The membrane was then incubated with Dylight 800-labeled goat anti-rabbit IgG and Dylight 680-labeled goat anti-mouse IgG (Abbkine, China) as secondary antibodies.

### IF assay

DF-1 cells were inoculated in glass-bottomed confocal dishes (in a 12-well setup) and incubated for one day until the fusion of cells occurred. In accordance with the guidelines provided by the manufacturer, the cells were cotransfected with the pCMV-Flag V and pCMV-Myc-DNAJA2 plasmids via the Lipofectamine 3000 reagent (Invitrogen, USA). At 36 h after transfection, the cells were fixed with 4% paraformaldehyde for 10 min at ambient temperature, followed by permeabilization with 0.5% Triton X-100 for an additional 10 min. The blocking process utilized QuickBlock™ immunostaining buffer (Beyotime, China) for 30 min at ambient temperature. For immunostaining, the samples were incubated for one hour at room temperature with mouse anti-Myc and rabbit anti-Flag monoclonal antibodies diluted at a ratio of 1:500. The process involved a one-hour incubation with Alexa Fluor 488-conjugated anti-mouse IgG and Alexa Fluor 594-conjugated anti-rabbit IgG secondary antibodies at a dilution of 1:100. The nuclei were counterstained with DAPI for five minutes. The stained cells were analyzed via a Leica confocal laser scanning microscope, and the resulting images were processed via Leica Application Suite X software.

### Molecular docking analysis

The structural models of V and DNAJA2 were obtained from the AlphaFold Protein Structure Database (https://alphafold.ebi.ac.uk/). Protein-protein docking was performed using the HDOCK server, with V as the ligand and DNAJA2 as the receptor. The docking results were ranked based on the HDOCK scoring function, and the highest-scoring complex was selected for further analysis. The selected model was visualized and analyzed using PyMOL (https://www.schrodinger.com/pymol).

### Statistical analysis

This study used GraphPad Prism software, version 5 (GraphPad Software, La Jolla, CA, USA, 2012). Comparisons between different treatment groups were performed via an unpaired, two-tailed Student’s t test assuming unequal variances (* *p* < 0.05, ** *p* < 0.01, *** *p* < 0.001, **** *p* < 0.0001, and ns no statistical significance).

## Results

### DNAJA2 interacts with the NDV V protein, and NDV infection upregulates DNAJA2 expression

**Fig. 1:**
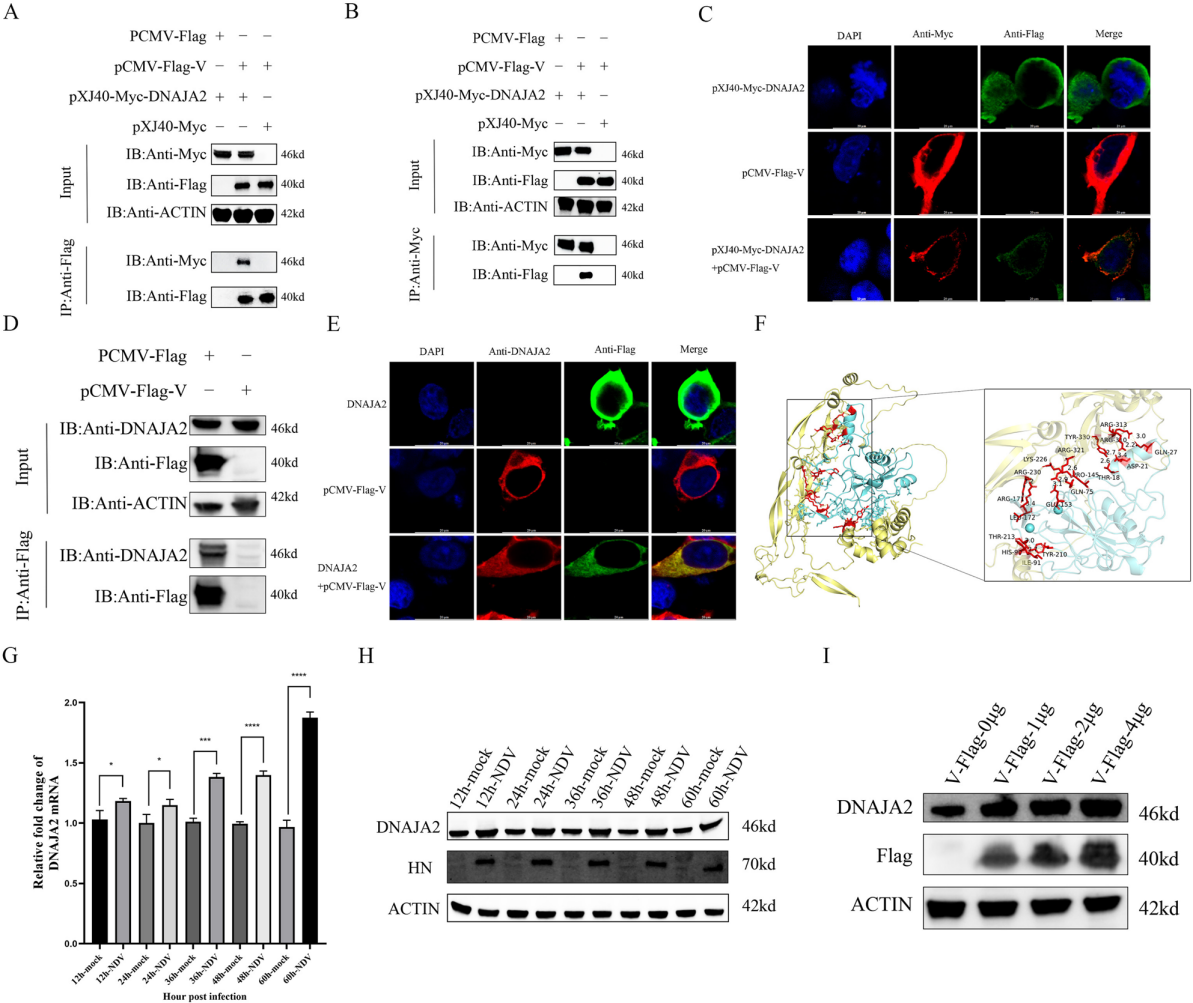
DNAJA2 interacts with the NDV V protein, and NDV infection upregulates DNAJA2 expression. **A** DF-1 cells were transiently transfected with either pCMV-Flag or pCMV-Flag-V constructs along with pXJ40-Myc or pXJ40-Myc-DNAJA2 plasmids for 24 h, followed by anti-Flag immunoprecipitation. Both the input lysates and immunoprecipitated (IP) complexes were examined via western blot analysis with anti-Flag and anti-Myc antibodies. **B** In separate experiments, DF-1 cells were transiently transfected with pCMV-Flag or pCMV-Flag-V combined with pXJ40-Myc or pXJ40-Myc-DNAJA2 for 24 h before being subjected to anti-Myc immunoprecipitation. Protein samples from both the input and IP fractions were evaluated via western blotting via identical antibody pairs. **C** Confocal microscopy analysis was performed on DF-1 cells coexpressing Flag-V (green fluorescence) and Myc-DNAJA2 (red fluorescence), with antigen detection achieved through rabbit anti-Flag polyclonal antibodies and mouse anti-Myc monoclonal antibodies, respectively. Nuclear visualization was accomplished via DAPI counterstaining (blue). **D** In separate experiments, DF-1 cells were transiently transfected with pCMV-Flag or pCMV-Flag-V for 24 h before being subjected to anti-Flag immunoprecipitation. Protein samples from both the input and IP fractions were evaluated via western blotting via identical antibody pairs. **E** Confocal microscopy analysis was performed on DF-1 cells coexpressing Flag-V (green fluorescence) and DNAJA2 (red fluorescence), with antigen detection achieved through rabbit anti-DNAJA2 polyclonal antibodies and mouse anti-Flag monoclonal antibodies, respectively. Nuclear visualization was accomplished via DAPI counterstaining (blue). **F** Use the molecular docking server HDOCK to analyze the interaction between V and DNAJA2. Employ the 3D molecular structure visualization software Pymol to analyze the interaction regions between V and DNAJA2. **G** NDV-infected DF-1 cells were collected at various time points (12, 24, 36, 48, and 60 h post-infection) for quantitative assessment of DNAJA2 expression levels via qRT‒PCR analysis. **H** Following NDV infection of DF-1 cells at 12-, 24-, 36-, 48- and 60-hour intervals, DNAJA2 protein levels were quantified via western blot analysis, followed by assessment with ImageJ software for data processing. **I** After 24 h of transfection with 0–4 µg of pCMV-Flag-V, DNAJA2 and Flag protein levels were examined via western blotting, and quantitative analysis was performed via ImageJ software

In view of IP-MS, which has been completed by our laboratory [15], we searched for proteins related to NDV V and obtained DNAJA2. To determine the interaction of DNAJA2 with NDV V proteins, the construct pXJ40-Myc-DNAJA2 was cotransfected with pCMV-Flag-V in DF-1 cells, and cell lysis buffer was collected for Co-IP. The results revealed that DNAJA2 interacts with the NDV V protein (Fig. 1A, B). In addition, confocal microscopy revealed that the DNAJA2 and NDV V proteins colocalized within the cytoplasm of DF-1 cells (Fig. 1C). To further confirm the interaction between DNAJA2 and the NDV V protein, a Flag-tagged V expression plasmid was transfected into DF-1 cells. Both laser confocal microscopy and co-immunoprecipitation analyses demonstrated that Flag-V colocalizes and interacts with endogenous DNAJA2 (Fig. 1D, E, Fig. S3). To define the structural basis of this interaction, three-dimensional models of V were generated using AlphaFold and subjected to docking analysis with DNAJA2 via the HDOCK platform. The simulations revealed that V binds DNAJA2, with binding energies ranging from − 214.43 kcal·mol⁻¹ to −277.61 kcal·mol⁻¹ for the V-DNAJA2 complexes (Fig. S1). Structural visualization confirmed these results, showing DNAJA2 (yellow) bound to V (cyan) (Fig. 1F). DF-1 cells infected with NDV (MOI = 1) were used to confirm the effect of NDV on DNAJA2 expression. The mRNA level of DNAJA2 was detected via qRT‒PCR. The results revealed a significant increase in DNAJA2 expression levels from 12 to 60 h.p.i. (Fig. 1G). Western blotting analysis revealed that NDV infection significantly increased DNAJA2 protein expression from 12 to 60 h.p.i. (Fig. 1H). To determine whether the increase in DNAJA2 expression observed during NDV infection is directly attributable to the NDV V protein, DF-1 cells were transfected with increasing amounts of Flag-tagged V plasmid. Western blot analysis showed a clear dose-dependent upregulation of endogenous DNAJA2 protein in response to V overexpression (Fig. 1I). These results indicate that the induction of DNAJA2 is specifically mediated by the NDV V protein rather than by other cellular stimuli associated with viral infection. Collectively, these findings indicate that DNAJA2 physically associates with the NDV V protein and colocalizes with it in the cytoplasm, and that DNAJA2 expression is significantly induced both during NDV infection and upon dose-dependent overexpression of the V protein.

### DNAJA2 inhibits NDV replication 

**Fig. 2:**
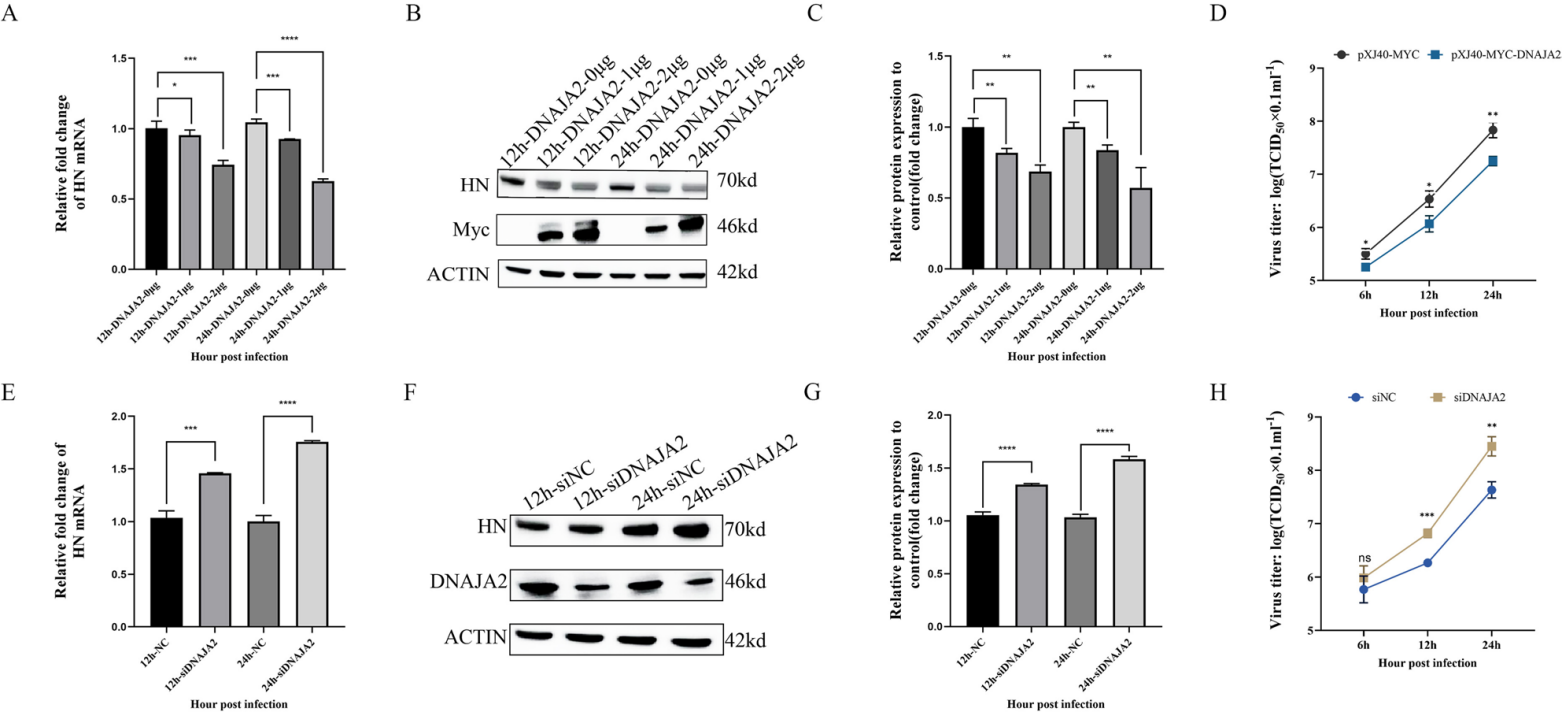
DNAJA2 inhibits NDV replication. **A** DF-1 cells were transfected with the pXJ40-Myc-DNAJA2 plasmid at concentrations of 1 and 2 µg for 24 h prior to 12/24-hour NDV infection. Viral HN mRNA expression was quantified through qRT‒PCR assays. **B**, **C** After 24 h of transfection with 1–2 µg of pXJ40-Myc-DNAJA2 and subsequent 12/24-hour NDV exposure, HN protein levels were examined via western blotting, and quantitative analysis was performed via ImageJ software. **D** Reed-Muench methodology was employed to determine virus titers in DNAJA2-overexpressing DF-1 cells. **E** After 24 h of treatment with siNC or siDNAJA2 followed by NDV infection (12/24 h), HN mRNA expression patterns were assessed through qRT‒PCR analysis. **F**, **G** Protein expression profiles of HN in NDV-infected DF-1 cells pretreated with siNC/siDNAJA2 (for 24 h) were determined via western blotting and subsequently evaluated via ImageJ. **H** Viral load quantification in DNAJA2-silenced DF-1 cell populations was conducted via the Reed‒Muench calculation method

To assess the role of DNAJA2 in NDV replication, DF-1, which is characterized by DNAJA2 overexpression and interference, was used in NDV infection studies. In DNAJA2-overexpressing cells, pXJ40-Myc-DNAJA2 was transfected at 1 and 2 µg into DF-1 cells. The expression of the NDV HN gene was measured by qRT‒PCR in DF-1 cells overexpressing DNAJA2. Compared with the control, DF-1 cells overexpressing DNAJA2 presented significantly decreased NDV HN gene mRNA levels (Fig. 2A). To further confirm this, DF-1 cells were transfected with 1–2 µg of pXJ40-Myc-DNAJA2 and infected with NDV. Western blotting was used to measure the protein levels of NDV HN. The results indicated that DF-1 cells overexpressing pXJ40-Myc-DNAJA2 presented significantly decreased expression of the NDV HN protein (Fig. 2B, C). Viral titers in DF-1 cells overexpressing pXJ40-Myc-DNAJA2 and pXJ40-Myc were also detected. The results revealed that viral titers in cells overexpressing DNAJA2 were significantly decreased, approximately 3- to 6-fold (Fig. 2D). In DNAJA2-silenced cells, the NDV HN gene mRNA levels were significantly increased (Fig. 2E). The HN protein levels exhibited a comparable trend (Fig. 2F, G). Viral titers were assessed in DF-1 cells with DNAJA2 interference. The results revealed that the viral titer was obviously increased approximately 5-fold in DF-1 cells with DNAJA2 interference (Fig. 2H). These results revealed that DNAJA2 inhibits the replication of NDV in DF-1 cells.

### The 101–367 amino acid domain of DNAJA2 interacts with NDV V proteins and exerts antiviral effects

**Fig. 3:**
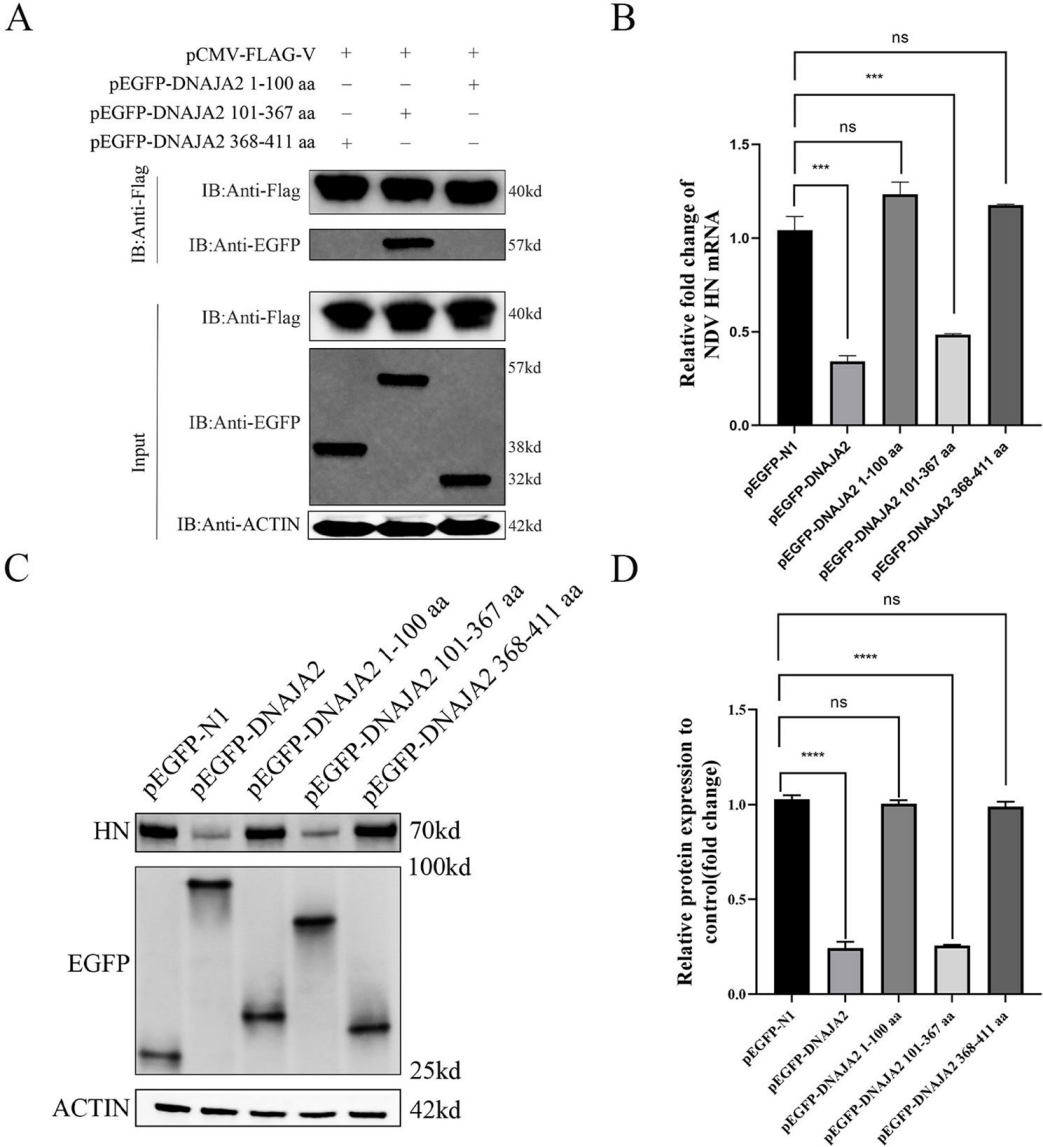
The 101–367 amino acid domain of DNAJA2 interacts with NDV V proteins and exerts antiviral effects. **A** DF-1 cells were transfected for 24 h with either pEGFP-DNAJA2 fragments (1–100 aa, 101–367 aa, or 368–411 aa) or pCMV-Flag-V, followed by anti-Flag immunoprecipitation. Both the lysate samples and immunoprecipitated complexes were examined via western blot analysis with anti-Flag and anti-EGFP antibodies. **B** Following 24 h of transfection with pEGFP-N1, full-length pEGFP-DNAJA2, or its truncated variants (1–100 aa, 101–367 aa, 368–411 aa), DF-1 cells were infected with NDV for an additional 24 h. Quantitative RT‒PCR was performed to measure the NDV HN mRNA expression levels. **C**, **D** In parallel experiments, NDV-infected DF-1 cells transfected with identical plasmid constructs were subjected to western blot analysis to detect HN protein levels, with subsequent quantification performed via ImageJ software. The experimental timeline maintained consistent 24-hour periods for both the transfection and viral infection phases across all conditions

To determine the DNAJA2 domain required for interaction with NDV V, we created a series of EGFP-tagged truncated DNAJA2 mutants. DF-1 cells expressing these mutants were collected and analyzed by anti-Flag bead immunoprecipitation. The findings revealed that the DNAJA2 1–100 amino acid domain and the DNAJA2 368–411 amino acid domain were unable to engage with NDV V, in contrast to the DNAJA2 101–367 amino acid domain, which engaged with NDV V (Fig. 3A). This result is consistent with the previous results of molecular docking. To characterize the relationships between the domains of DNAJA2, which interact with the NDV V protein, and its antiviral capacity, pEGFP-N1, pEGFP-DNAJA2 and the truncated mutants of DNAJA2, pEGFP-DNAJA2 amino acids 1–100, pEGFP-DNAJA2 amino acids 101–367, and pEGFP-DNAJA2 amino acids 368–411, were transfected into DF-1 and infected with NDV, after which the mRNA expression of the NDV HN gene was determined via qRT‒PCR. The results demonstrated that the NDV HN gene mRNA levels significantly decreased in cells transfected with pEGFP-DNAJA2 or pEGFP-DNAJA2 amino acids 101–367 (Fig. 3B). The expression of NDV HN in cells overexpressing the amino acids pEGFP-N1, pEGFP-DNAJA2, pEGFP-DNAJA2 amino acids 1–100, pEGFP-DNAJA2 amino acids 101–367, and pEGFP-DNAJA2 amino acids 368–411 was determined via western blotting. The expression of the NDV HN protein significantly decreased in cells overexpressing the pEGFP-DNAJA2 or pEGFP-DNAJA2 amino acids 101–367 (Fig. 3C, D). These findings suggest that the DNAJA2 amino acid domain 101–367 is the major domain that interacts with the NDV V protein and has antiviral activity similar to that of the parent DNAJA2.

### DNAJA2 enhances the innate immune response in NDV infection

**Fig. 4:**
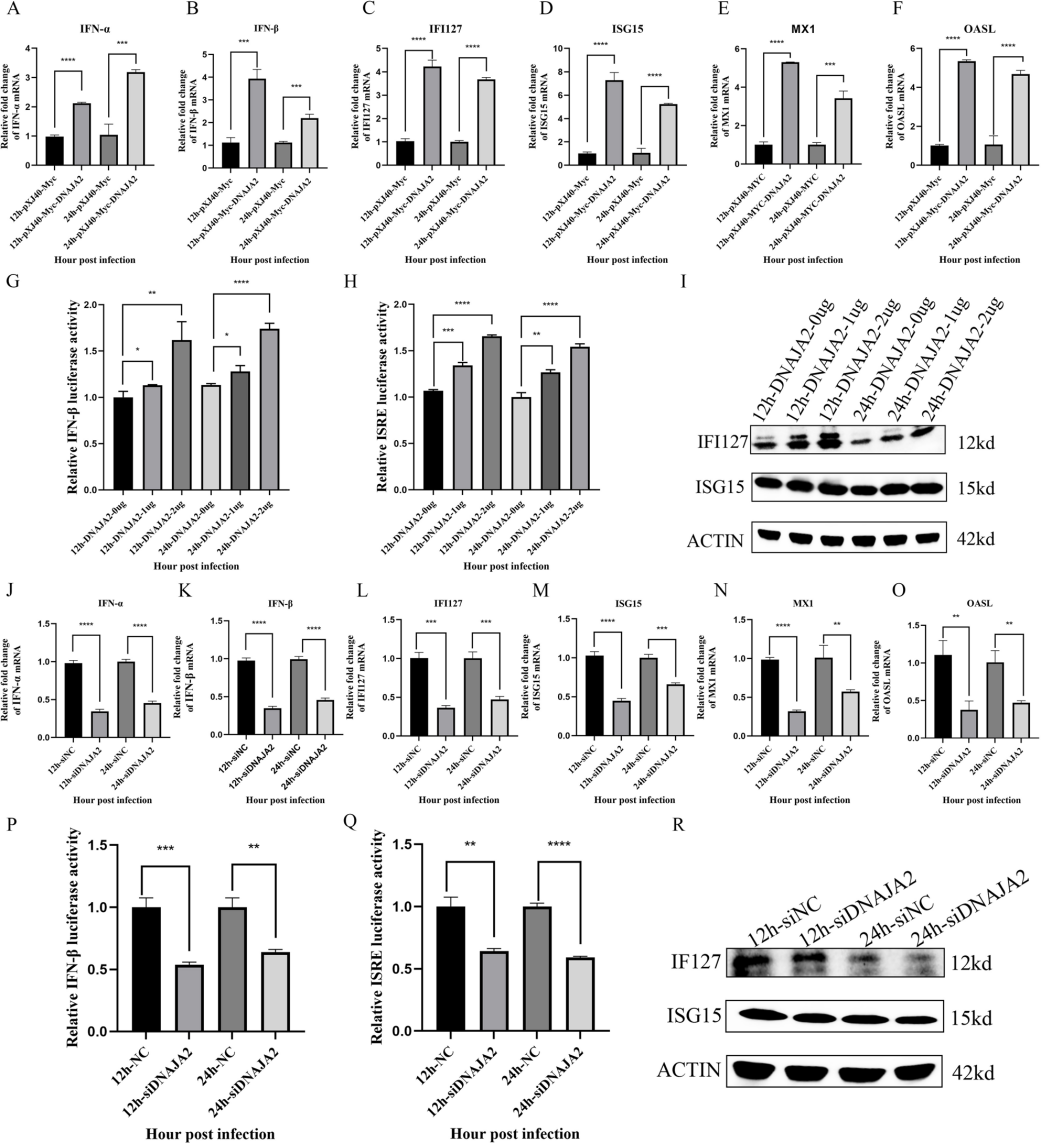
DNAJA2 enhances the innate immune response in NDV infection. **A**-**F** DF-1 cell cultures were transfected for 24 h with either pXJ40-Myc or pXJ40-Myc-DNAJA2 constructs, followed by Newcastle disease virus infection for 12–24 h. The subsequent quantification of IFN-α, IFN-β, IFI127, ISG15, MX1, and OSAL transcript levels was performed through quantitative reverse transcription PCR analysis. **G** In separate experiments, DF-1 cells were cotransfected with various concentrations (1–2 µg) of pXJ40-Myc/pXJ40-Myc-DNAJA2 alongside dual-reporter plasmids containing IFN-β promoter-driven firefly luciferase and constitutive Renilla luciferase constructs. Following 24 h of incubation and subsequent NDV challenge (12/24 h), luminescence signals from both reporters were measured via a multimode microplate reader. **H** A similar experimental setup was replicated via reporter plasmids containing interferon-stimulated response element (ISRE) regulatory sequences instead of IFN-β promoters, with luminescent measurements conducted under identical infection conditions. **I** Additional investigations involving DF-1 cell transfection with pXJ40-Myc or pXJ40-Myc-DNAJA2 constructs. DF-1 cells were infected with pXJ40-Myc-DNAJA2 at concentrations of 1–2 µg, followed by NDV infection for 12- and 24-hour periods. Western blot analysis was performed to quantify IFI127 and ISG15 protein expression levels, and densitometric measurements were conducted via ImageJ software. **J**‒**O** In separate experiments, DF‒1 cultures were transfected with either siNC or siDNAJA2 for 24 h before NDV challenge at 12-h and 24-h intervals. Quantitative reverse transcription PCR was used to measure the transcriptional levels of the IFN-α, IFN-β, IFI127, ISG15, MX1, and OSAL genes. **P** Dual-luciferase reporter assays were performed by cotransfecting DF-1 cells with siNC/siDNAJA2, the IFN-β promoter-driven firefly luciferase construct, or the Renilla luciferase control plasmid for 24 h prior to NDV treatment. Luminescence signals from both reporters were quantified via a multimode microplate reader following viral exposure at specified time points. **Q** A similar reporter methodology was used with an ISRE-containing firefly luciferase plasmid alongside a Renilla control, with the transfected cells subjected to NDV infection. The relative luminescence units for both reporter systems were determined through enzymatic detection at 12 and 24 h postinfection. **R** DF-1 cell models were transfected with either siNC or siDNAJA2 constructs, followed by NDV infection for 12- and 24-hour durations. Subsequent western blot analysis was used to quantify the protein expression profiles of IFI127 and ISG15, and densitometric measurements were performed with ImageJ software for statistical evaluation

To determine whether DNAJA2 inhibits viral replication by activating the type I interferon pathway, it is effective to detect the mRNA levels of interferon-α (IFN-α), interferon-β (IFN-β), IFI127, ISG15, MX1, and OSAL in DF-1 cells overexpressing DNAJA2 and infected with NDV. The results revealed that the mRNA expression levels of IFN-α, IFN-β, IFI127, ISG15, MX1, and OSAL were significantly increased (Fig. 4A-F). Subsequently, 1 and 2 µg of pXJ40-Myc-DNAJA2 and the firefly fluorescent plasmid with the IFN-β promoter or ISRE response element and the sea kidney fluorescent plasmid were transfected into DF-1 cells, which were infected with NDV, and the fluorescence intensities of the firefly fluorescence and sea kidney fluorescence of the cells were detected at 24 h. The results revealed that DNAJA2 significantly elevated the activity of the IFN-β promoter (Fig. 4G) and the ISRE response element (Fig. 4H). To further confirm this, DF-1 cells were transfected with pXJ40-Myc-DNAJA2 at 1 and 2 µg and infected with NDV. The results indicated that compared with pXJ40-Myc-Myc-DNAJA2-overexpressing DF-1 cells, pXJ40-Myc-DNAJA2-overexpressing DF-1 cells significantly increased the protein expression of IFI127 and ISG15 (Fig. 4I). In DF-1 cells with DNAJA2 interference, the RNA levels of IFN-α, IFN-β, IFI127, ISG15, MX1, and OSAL were also detected. The results revealed that the mRNA expression levels of IFN-α, IFN-β, IFI127, ISG15, MX1, and OSAL were obviously reduced at 12 and 24 h after infection (Fig. 4J-O). The activities of the IFN-β promoter (Fig. 4P) and ISRE response elements (Fig. 4Q) were also significantly decreased in DF-1 cells that were DNAJA2-interfered and infected with NDV. Similarly, the protein levels of IFI127 and ISG15 were significantly decreased in cells with DNAJA2 interference and those infected with NDV (Fig. 4R). These results demonstrate that DNAJA2 enhances the innate immune response during NDV infection.

### DNAJA2 targets the NDV V protein to activate the MDA5-MAVS signaling pathway

**Fig. 5:**
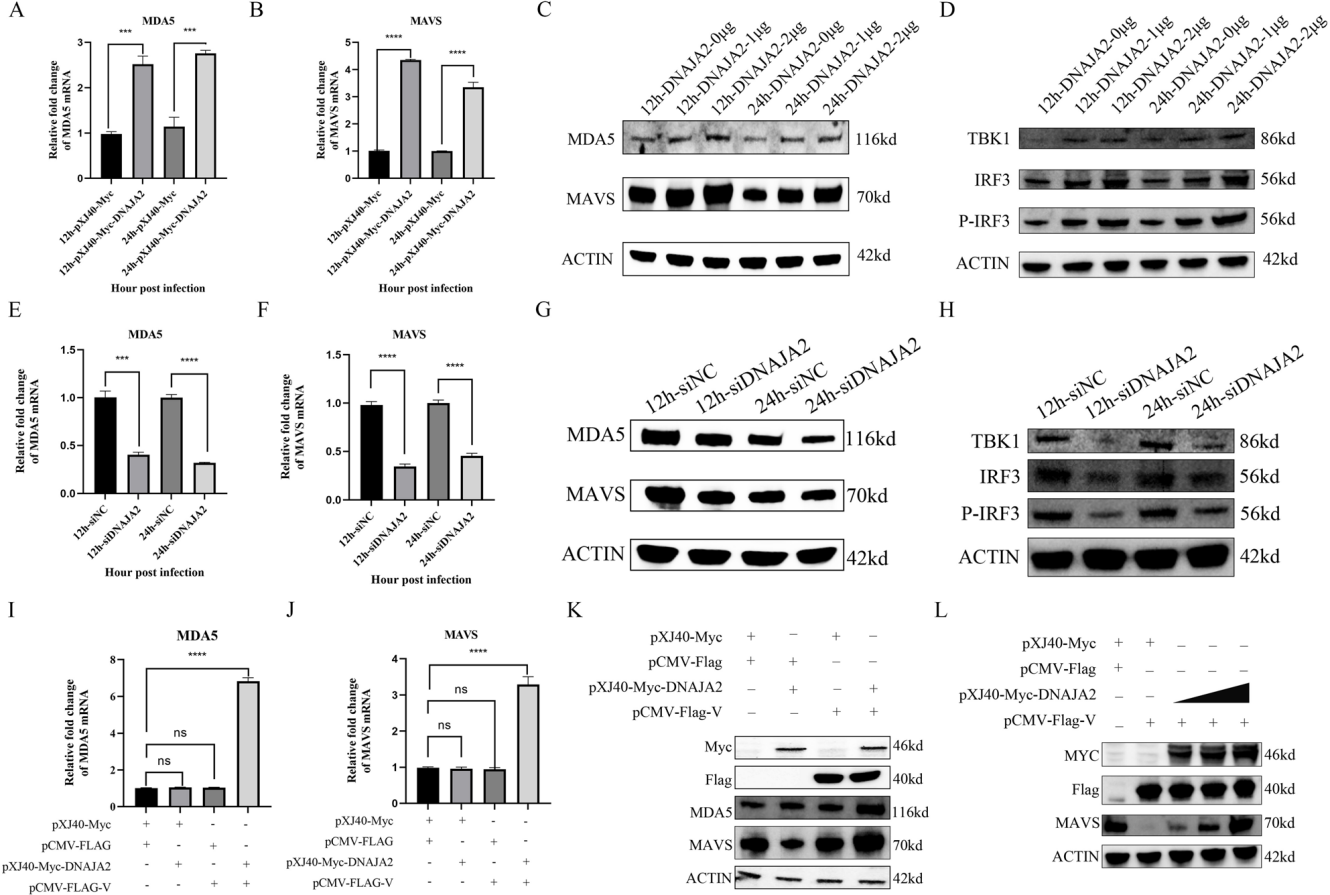
DNAJA2 targets the NDV V protein to activate the MDA5-MAVS signaling pathway. **A**, **B** DF-1 cells were introduced with either pXJ40-Myc or pXJ40-Myc-DNAJA2 plasmids for one day prior to exposure to NDV for periods of 12 and 24 h. Quantitative reverse transcription PCR was used to measure the MDA5 and MAVS mRNA expression levels. **C** Following transfection with various concentrations (1–2 µg) of pXJ40-Myc or pXJ40-Myc-DNAJA2 constructs, DF-1 cells were infected with NDV for identical time intervals. Western blot analysis coupled with ImageJ software quantification was utilized to assess the MDA5 and MAVS protein concentrations. **D** DF-1 cells were transfected with different amounts of pXJ40-Myc or pXJ40-Myc-DNAJA2 (1–2 µg) and subsequently infected with NDV for the same duration. Western blot analysis, followed by densitometric quantification using ImageJ software, was performed to evaluate the expression levels of TBK1, IRF3, and P-IRF3. **E**, **F** Experimental cells received either control siRNA (siNC) or DNAJA2-targeting siRNA (siDNAJA2) 24 h before NDV challenge. Subsequent mRNA quantification of target genes was performed via qRT‒PCR methodology. **G** Protein expression levels of MAVS and MDA5 in siRNA-transfected DF-1 cells following NDV infection were examined by immunoblotting, followed by densitometric analysis. **H** Protein expression levels of TBK1, IRF3, and P-IRF3 in siRNA-transfected DF-1 cells following NDV infection were assessed by immunoblotting and quantified by densitometric analysis. **I**, **J** Cotransfection experiments combining pXJ40-Myc or pXJ40-Myc-DNAJA2 with pCMV-Flag or pCMV-Flag-V vectors were conducted over 24 h, with subsequent evaluation of transcriptional changes in MDA5 and MAVS via qRT‒PCR. **K** DF-1 cells were cotransfected with combinations of pXJ40-Myc or pXJ40-Myc-DNAJA2 alongside pCMV-Flag or pCMV-Flag-V vectors. Western blot analysis was performed to evaluate MDA5 and MAVS protein expression, with subsequent quantification conducted through ImageJ software. **L** DF-1 cells were co-transfected with a fixed amount of V protein and increasing amounts of DNAJA2, followed by assessment of MAVS expression. MAVS protein levels were measured by immunoblotting and quantified by densitometric analysis

The MDA5-MAVS signaling pathway is essential in NDV infection. To verify whether DNAJA2 activates the MDA5-MAVS signaling pathway, DF-1 cells were transfected with pXJ40-Myc and pXJ40-Myc-DNAJA2 and subsequently infected with NDV. qRT‒PCR and WB were used to measure the expression of MDA5 and MAVS mRNA and protein, respectively. Compared with those in the control, the mRNA levels of MDA5 and MAVS were significantly increased (Fig. 5A, B), and the protein levels showed a similar trend (Fig. 5C). Next, we validated the activation of the MDA5-MAVS pathway by measuring downstream proteins at the protein level. Significant increases in TBK1, IRF3, and P-IRF3 further confirmed MDA5-MAVS pathway activation (Fig. 5D). The expression of MDA5 and MAVS was also detected in DF-1 cells with interference with the expression of DNAJA2. qRT‒PCR revealed that the mRNA levels of MDA5 and MAVS were obviously lower in cells with DNAJA2 interference than in the control cells (Fig. 5E, F). Western blot analysis revealed a significant decrease in the protein levels of MDA5 and MAVS in cells with DNAJA2 interference (Fig. 5G). Simultaneously, the protein levels of TBK1, IRF3, and P-IRF3 were significantly decreased (Fig. 5H). NDV V inhibits the expression of MDA5 and degrades MVAS via the ubiquitination pathway, which inhibits the innate immune response [7]. To determine whether DNAJA2 targets V to activate the MDA5-MAVS signaling pathway, pXJ40-Myc or pXJ40-Myc-DNAJA2 and pCMV-Flag or pCMV-Flag-V were transfected into DF-1 cells. Compared with those of the control, the mRNA levels of MDA5 and MAVS were obviously increased only in DF-1 cells transfected with pXJ40-Myc-DNAJA2 and pCMV-Flag-V (Fig. 5I, J). The protein levels of MDA5 and MAVS were also significantly increased only in the cells transfected with pXJ40-Myc-DNAJA2 and pCMV-Flag-V (Fig. 5K). To further investigate the role of DNAJA2 in V protein-mediated immunosuppression, we co-transfected cells with a fixed dose of V protein and increasing amounts of DNAJA2. The results showed that DNAJA2 not only reversed the V protein-induced degradation of MAVS but also activated MAVS, increasing its levels above baseline (Fig. 5L). These results suggest that DNAJA2 activates the MDA5-MAVS signaling pathway by targeting the NDV V protein.

## Discussion

NDV is a highly contagious avian virus that is characterized by its ability to transmit across species [16]. The NDV V protein, which is recognized as an important virulence factor, has been shown to interact with a wide range of host proteins [17]. To identify potential antiviral factors that interact with the NDV V protein, we analyzed previous immunoprecipitation-mass spectrometry results [15] and identified DNAJA2 as a key interacting protein. Notably, the overexpression of DNAJA2 inhibited NDV replication, and DNAJA2 interacted with NDV V through the 101–367 aa domain, which performs a major antiviral function. These findings suggest that DNAJA2 may function as a new type of restriction factor in antiviral defense.

Type I IFNs and ISGs are critical in host immune responses against NDV infection [18]. Like other paramyxoviruses, NDV uses multiple strategies to inhibit the production of IFNs and ISGs. NDV V protein can promote viral replication by hijacking and degrading host antiviral factors, but it can also be recognized by host defensive proteins that counteract its suppressive activity. These opposing actions reflect the virus–host “tug-of-war” during NDV infection. Research has shown that the NDV V protein can suppress IFN-β production through multiple mechanisms. One study reported that V promotes the ubiquitin-mediated degradation of DNMT3A, leading to reduced IRF7 expression and consequently diminished IFN-β transcription [19]. In addition, other work has demonstrated that the V protein suppresses IFN-β by facilitating RNF5-dependent degradation of MAVS, thereby disrupting downstream antiviral signaling [7]. NDV V proteins hijack host factors to promote viral replication. For example, NDV V proteins regulate cell proliferation and enhance viral replication by interacting with hnRNP H1 [20]. Moreover, host factors can inhibit viral replication by targeting the V protein of NDV. For example, EFTUD2 activates innate immunity by shearing MDA5 to inhibit viral replication [15]. Despite the numerous studies conducted to elucidate the potential biological functions of the NDV V protein, the interaction between NDV and DNAJA2, a family of heat shock proteins, remains poorly characterized.

DNAJA2, a molecular chaperone in the heat shock protein family, is usually activated to maintain protein stability during heat stress [21, 22]. Many studies have demonstrated the role of DNAJA2 in immunization. For example, DNAJA2 interacts with HAM1 to activate immunity [23], and DNAJA2 activates the cGAS-STING signaling pathway through aberrant mitosis [12]. These studies suggest that DNAJA2 has a positive regulatory function in immunity. The role of DNAJA2 in JEV infection has also been demonstrated, but in this study, DNAJA2 was identified as an NS3-interacting protein that promoted JEV replication [13]. However, the function of DNAJA2 in NDV replication is unclear.

Previous studies have demonstrated the immunosuppressive effects of NDV V [24]. On the basis of our study, we determined that DNAJA2 engages with NDV V and that DNAJA2 expression is upregulated which is driven by the NDV V protein during NDV infection. The overexpression of DNAJA2 in DF-1 cells inhibits NDV replication, whereas interference with DNAJA2 promotes NDV replication. We subsequently delineated the structural domains of DNAJA2 on the basis of NCBI. The 101–367 aa structural domain of DNAJA2 was found to be the main structural domain that interacts with NDV V and performs a major antiviral function. Owing to the immunosuppressive effects of NDV V, we subsequently examined the levels of type I interferons and ISGs. Type I interferons and ISGs were significantly upregulated in DNAJA2-overexpressing cells, whereas the inhibition of DNAJA2 significantly suppressed the levels of type I interferons and ISGs. This finding suggests that DNAJA2 upregulates innate immunity, which is suppressed by NDV V. Previous studies have demonstrated that NDV V proteins mainly inhibit the MDA5-MAVS signaling pathway [7, 25]; therefore, we wondered whether DNAJA2 upregulates the level of the MDA5-MAVS pathway. We examined the expression levels of MDA5,MAVS, TBK1, IRF3 and P-IRF3 and found that DNAJA2 promoted their expression. Further studies indicated that DNAJA2 specifically targets the NDV V protein, thereby regulating the MDA5-MAVS pathway. Several members of the DNAJ family have been characterized as activators of innate immune signaling [26], supporting the concept that DNAJA2 is part of the host antiviral machinery. Although DNAJA2 enhances type I IFN signaling in our study, its interaction with the NDV V protein is fully consistent with known virus–host dynamics. Viral antagonists frequently bind antiviral host factors not to activate them, but rather to inhibit, mislocalize, or neutralize their functions. This bidirectional competition is a classical feature of innate immune evasion: when the virus gains the advantage, it hijacks host proteins to promote immune suppression and replication; when the host prevails, antiviral proteins recognize viral components to trigger innate immune activation. A similar dual-direction regulatory relationship has been reported for porcine epidemic diarrhea virus (PEDV), where the N protein interacts with the host factor HNRNPA1. HNRNPA1 can sense the N protein and activate antiviral signaling, whereas PEDV counteracts this response by inducing the autophagic degradation of HNRNPA1 [27]. This example illustrates a common strategy in which viruses deliberately target immune-enhancing host factors, while hosts attempt to exploit the same interactions to mount an antiviral defense. Based on our data, we propose that DNAJA2 acts as a critical host defensive factor that recognizes and binds the NDV V protein to counteract V-mediated immune suppression. Through this interaction, DNAJA2 helps preserve and reinforce the MDA5–MAVS signaling axis during NDV infection, thereby contributing to an effective type I IFN response.

**Fig. 6:**
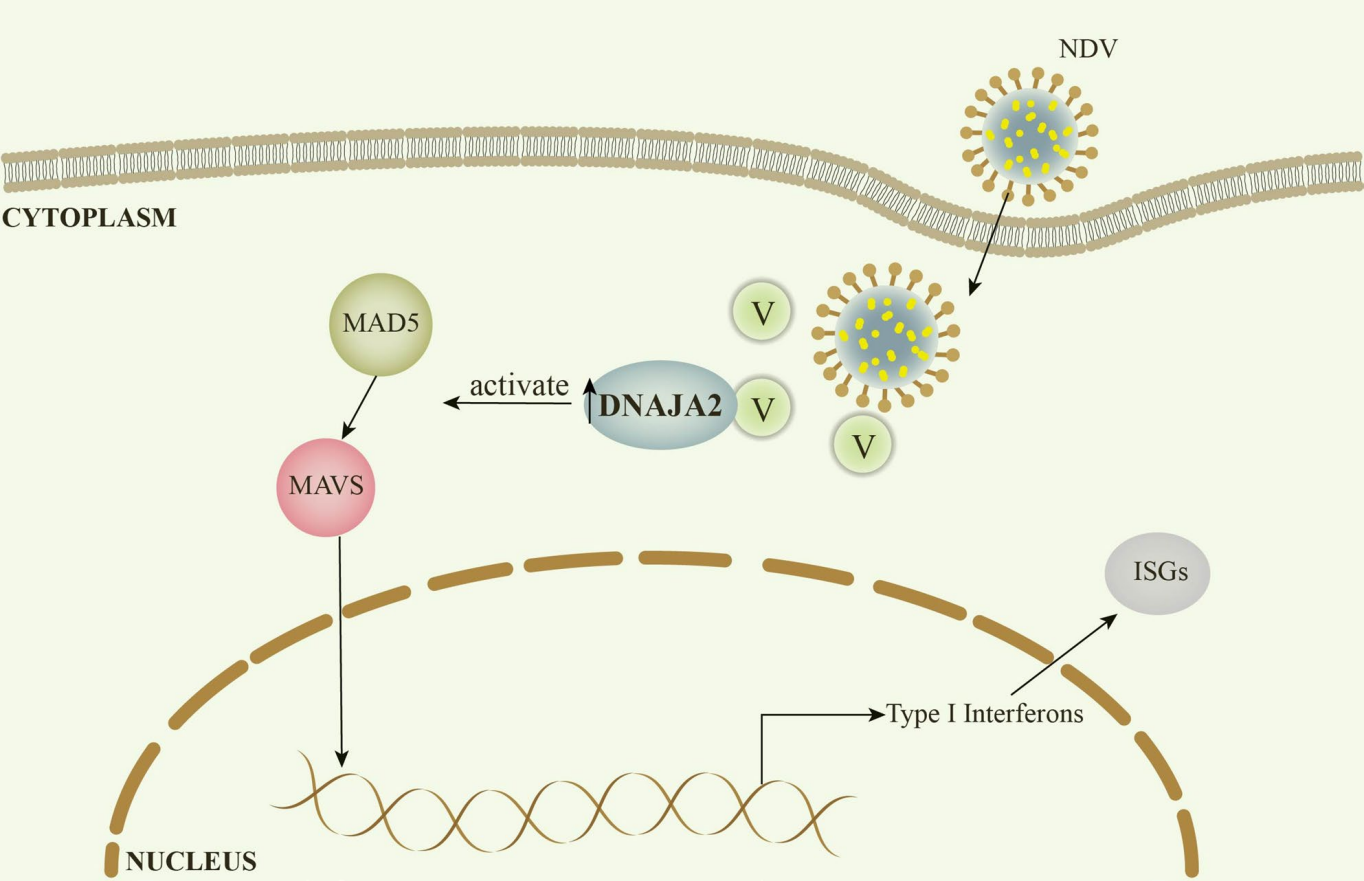
DNAJA2 inhibits Newcastle disease virus replication by targeting its V protein to modulate the MDA5-MAVS pathway

On the basis of our findings, we propose a model for the role of DNAJA2 in regulating innate immunity (Fig. 6). In this model, DNAJA2 exerts its antiviral effects by interacting with the NDV V protein, which activates the MDA5-MAVS pathway and induces the expression of type I interferons.

## Conclusion

In summary, the present study demonstrated that NDV V interacts with DNAJA2, thereby activating the MDA5-MAVS pathway and modulating IFN-β and ISG expression. These findings identify DNAJA2 as a host restriction factor that inhibits NDV replication. Our research contributes to the advancement of knowledge regarding the antiviral immunity of the heat shock protein family and identifies DNAJA2 as a potential therapeutic target.

## Supplementary Information

Supplementary Material 1.

Supplementary Material 2.

Supplementary Material 3.

## Data Availability

The datasets supporting the conclusions of this article are included within the article and its additional files.

## Abbreviations

Co-IP: Coimmunoprecipitation
DF-1: Chicken fibroblasts
DNAJA2: DnaJ Heat Shock Protein Family Member A2
HSP70: Heat shock protein 70
IFN-β: Interferon-β
IP-MS: Immunoprecipitation-mass spectrometry
ISGs: Interferon-stimulated genes
JEV: Japanese encephalitis virus
MAVS: Mitochondrial antiviral signaling protein
MDA5: Melanoma differentiation-associated gene 5
MS: Premass spectrometry
ND: Newcastle disease
NDV: Newcastle disease virus
PVDF: Polyvinylidene fluoride
SDS‒PAGE: Sodium dodecyl sulfate‒polyacrylamide gel electrophoresis
siRNA: small interfering RNA
qRT‒PCR: Real-time quantitative PCR
WB: Western blot
