# Supplementary material for: DNAJA2 inhibits Newcastle disease virus replication by targeting its V protein to modulate the MDA5-MAVS pathway
Source: BMC Microbiol. 2025 Dec 22;26:84. doi: 10.1186/s12866-025-04618-9 (PMC12870088; doi:10.1186/s12866-025-04618-9)
Supplement: Supplementary file 2 — Supplementary Material 2. [file 12866_2025_4618_MOESM2_ESM.docx]

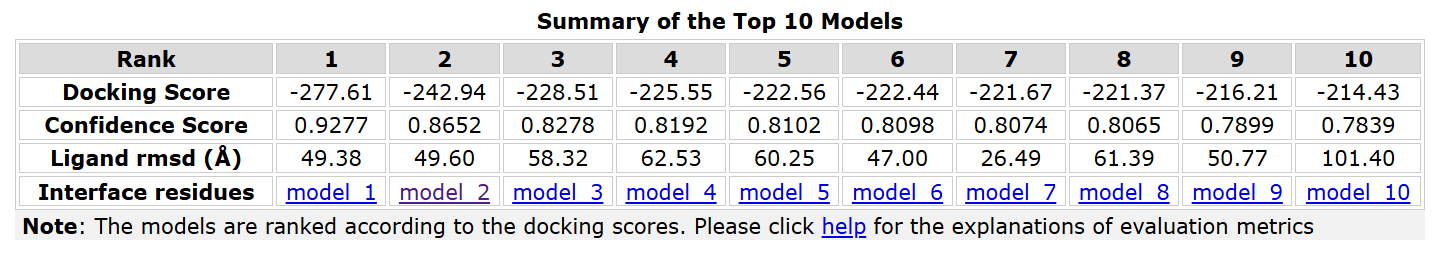


Fig. S1 Molecular docking analysis of V and DNAJA2 binding.

Binding energies for the first ten docking conformations of V with DNAJA2.

Fig. S2 Gray-scale analysis was conducted to quantify the relative expression of the target proteins. (A) Protein Gray Scale Analysis of Fig. 1H. (B) Protein Gray Scale Analysis of Fig. 1I. (C) Protein Gray Scale Analysis of Fig. 4I. (D) Protein Gray Scale Analysis of Fig. 4R. (E) Protein Gray Scale Analysis of Fig. 5C. (F) Protein Gray Scale Analysis of Fig. 5D. (G) Protein Gray Scale Analysis of Fig. 5G. (H) Protein Gray Scale Analysis of Fig. 5H. (I) Protein Gray Scale Analysis of Fig. 5K. (J) Protein Gray Scale Analysis of Fig. 5L.


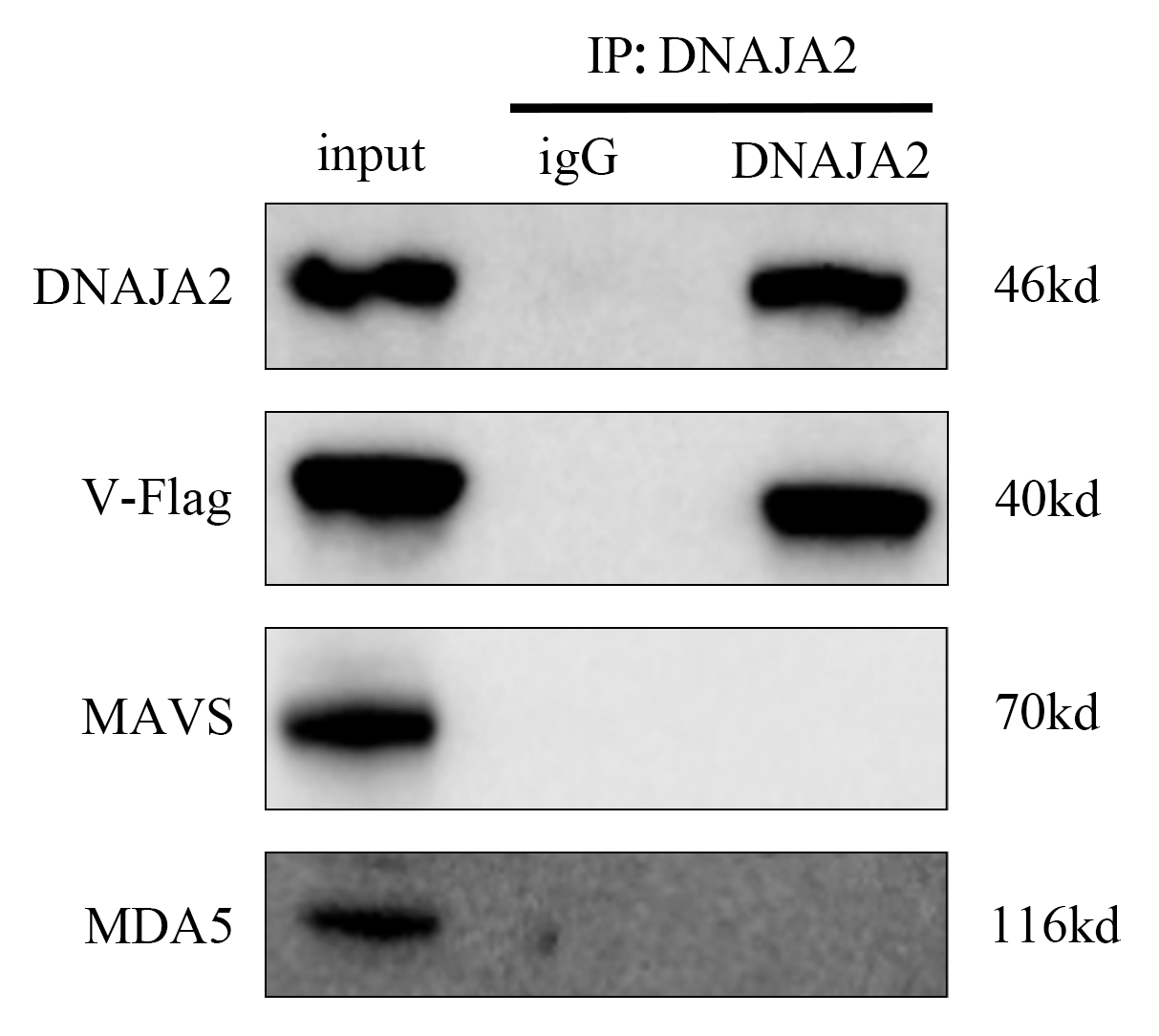


Fig. S3 DNAJA2 interact with V protein, but not directly interact with MAVS or MDA5.

DF-1 cells were transiently transfected with either pCMV-Flag or pCMV-Flag-V plasmids for 24 hours, followed by anti-DNAJA2 immunoprecipitation. Both the input lysates and immunoprecipitated (IP) complexes were examined via western blot analysis with anti-MDA5, anti-MAVS and anti-Flag antibodies.
