# Supplementary material for: DNAJA2 inhibits Newcastle disease virus replication by targeting its V protein to modulate the MDA5-MAVS pathway
Source: BMC Microbiol. 2025 Dec 22;26:84. doi: 10.1186/s12866-025-04618-9 (PMC12870088; doi:10.1186/s12866-025-04618-9)
Supplement: Supplementary file 3 — Supplementary Material 3. [file 12866_2025_4618_MOESM3_ESM.pdf]

Fig.1 A used

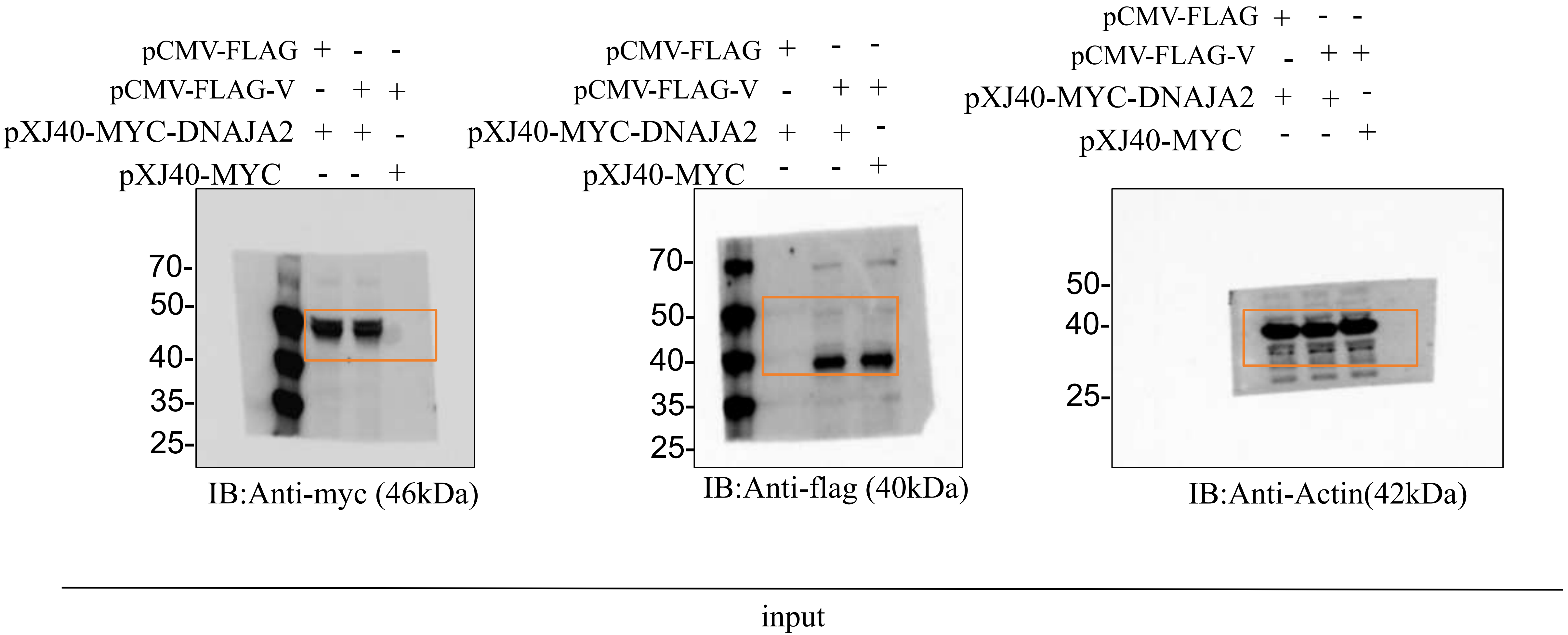

pCMV-FLAG + - -
pCMV-FLAG-V - + +
pXJ40-MYC-DNAJA2 + + -
pXJ40-MYC - - +
70-
50-
40-
35-
25-
IB:Anti-myc (46kDa)
pCMV-FLAG + - -
pCMV-FLAG-V - + +
pXJ40-MYC-DNAJA2 + + -
pXJ40-MYC - - +
70-
50-
40-
35-
25-
IB:Anti-flag (40kDa)
pCMV-FLAG + - -
pCMV-FLAG-V - + +
pXJ40-MYC-DNAJA2 + + -
pXJ40-MYC - - +
50-
40-
25-
IB:Anti-Actin(42kDa)
input

Fig.1 A used

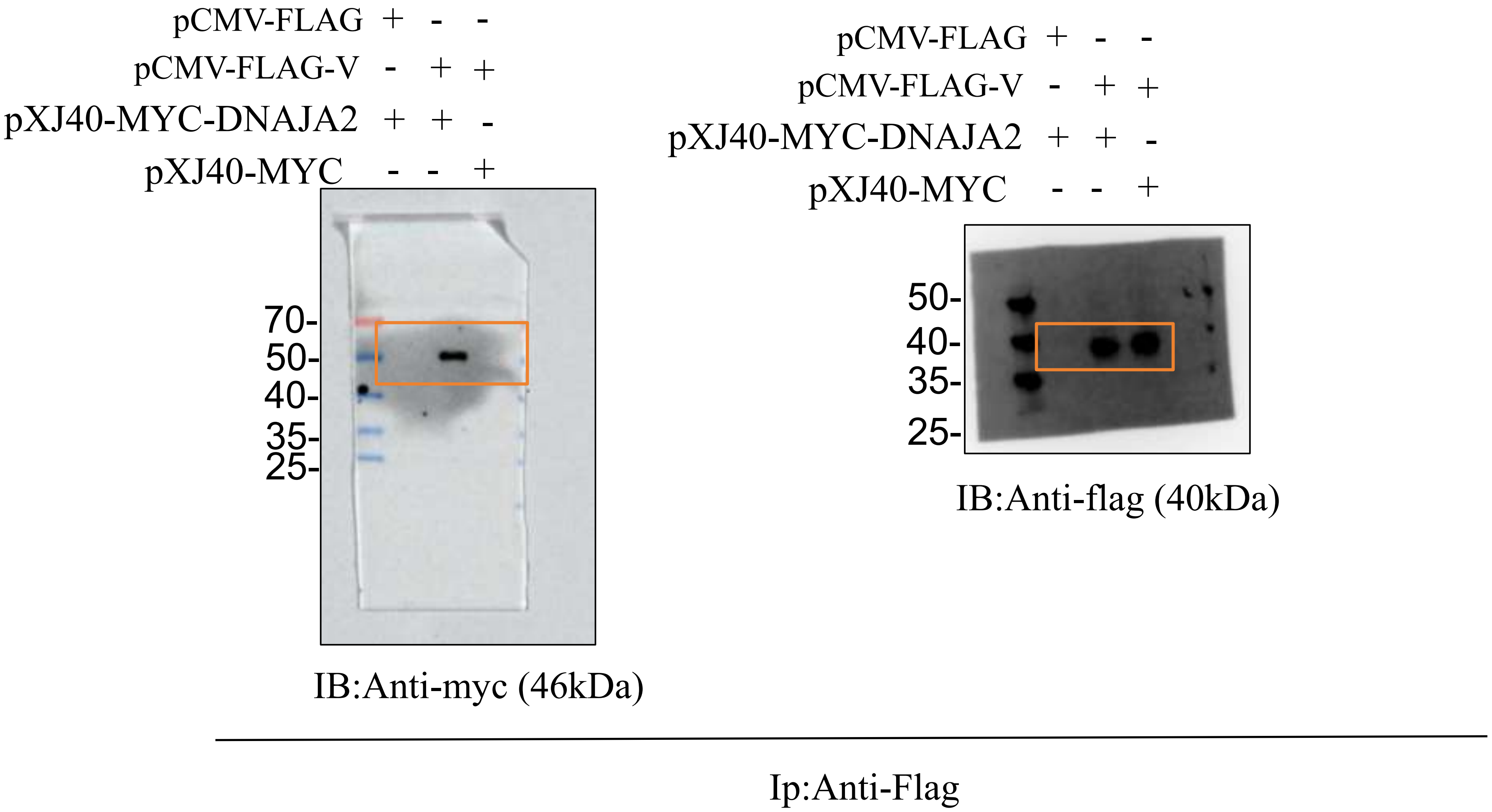
pCMV-FLAG + - -
pCMV-FLAG-V - + +
pXJ40-MYC-DNAJA2 + + -
pXJ40-MYC - - +
70-
50-
40-
35-
25-
IB:Anti-myc (46kDa)
pCMV-FLAG + - -
pCMV-FLAG-V - + +
pXJ40-MYC-DNAJA2 + + -
pXJ40-MYC - - +
50-
40-
35-
25-
IB:Anti-flag (40kDa)
Ip:Anti-Flag

Fig.1 B used

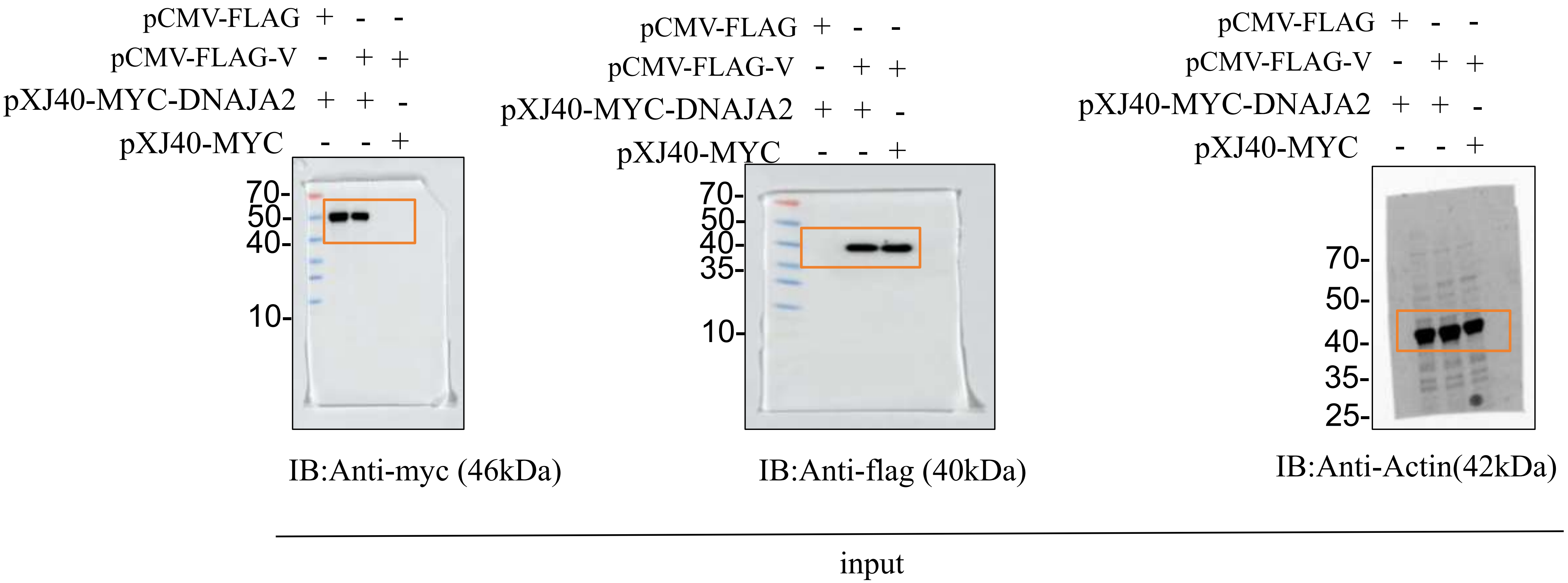
pCMV-FLAG + - -
pCMV-FLAG-V - + +
pXJ40-MYC-DNAJA2 + + -
pXJ40-MYC - - +
70-
50-
40-
10-
IB:Anti-myc (46kDa)
pCMV-FLAG + - -
pCMV-FLAG-V - + +
pXJ40-MYC-DNAJA2 + + -
pXJ40-MYC - - +
70-
50-
40-
35-
10-
IB:Anti-flag (40kDa)
pCMV-FLAG + - -
pCMV-FLAG-V - + +
pXJ40-MYC-DNAJA2 + + -
pXJ40-MYC - - +
70-
50-
40-
35-
25-
IB:Anti-Actin(42kDa)
input

Fig.1 B used

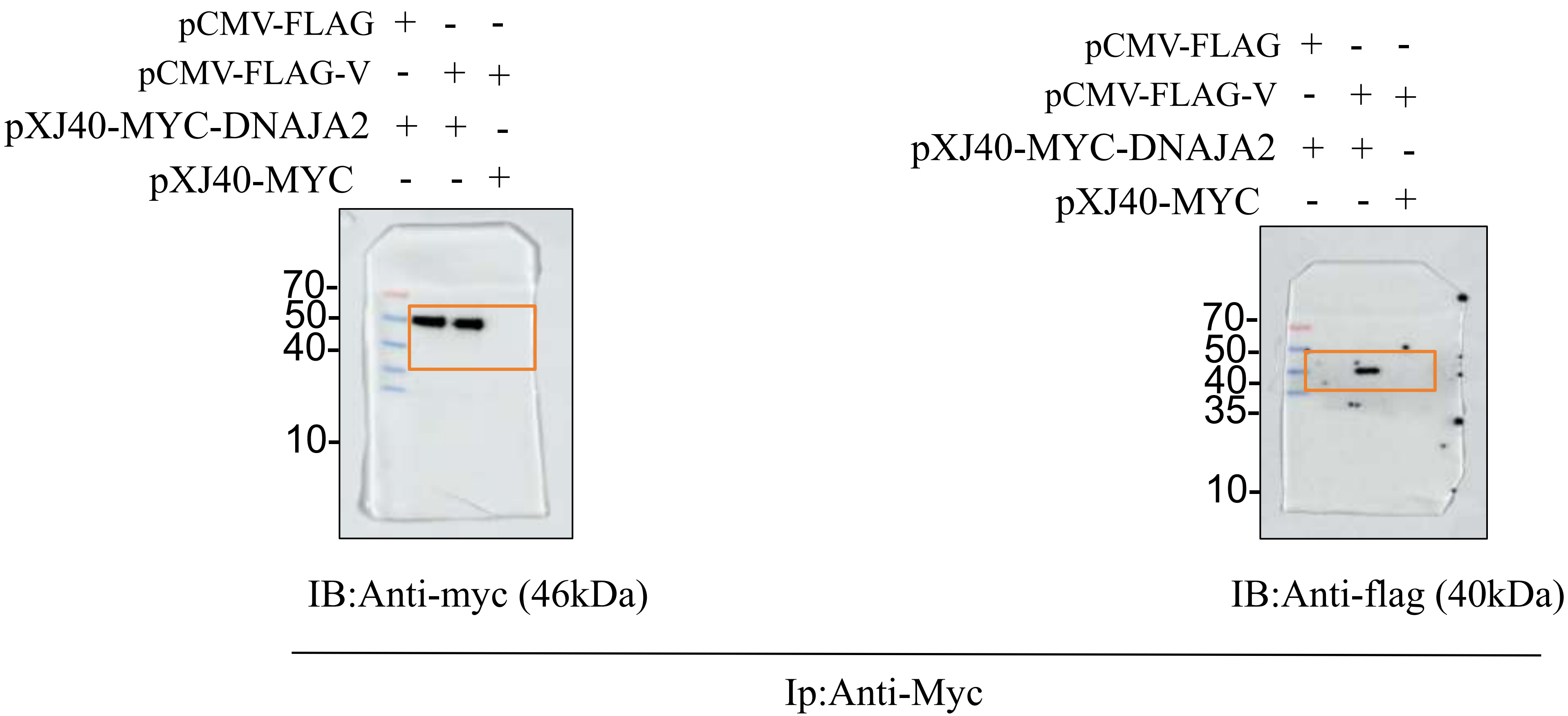

pCMV-FLAG + - -
pCMV-FLAG-V - + +
pXJ40-MYC-DNAJA2 + + -
pXJ40-MYC - - +
70-
50-
40-
10-
IB:Anti-myc (46kDa)
pCMV-FLAG + - -
pCMV-FLAG-V - + +
pXJ40-MYC-DNAJA2 + + -
pXJ40-MYC - - +
70-
50-
40-
35-
10-
IB:Anti-flag (40kDa)
Ip:Anti-Myc

Fig.1 D used

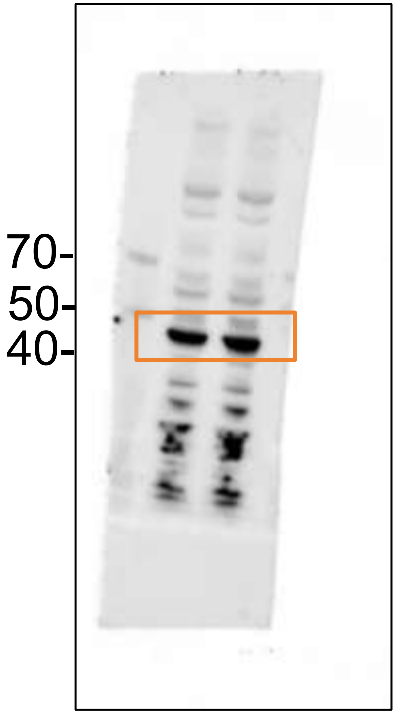

IB:Anti-DNAJA2 (46kDa)

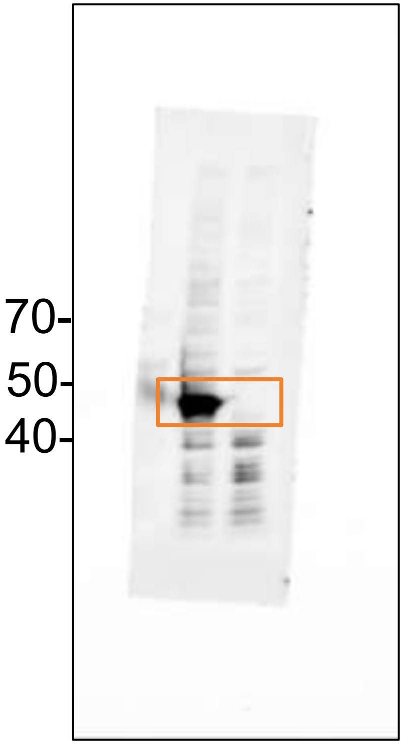

IB:Anti-flag (40kDa)

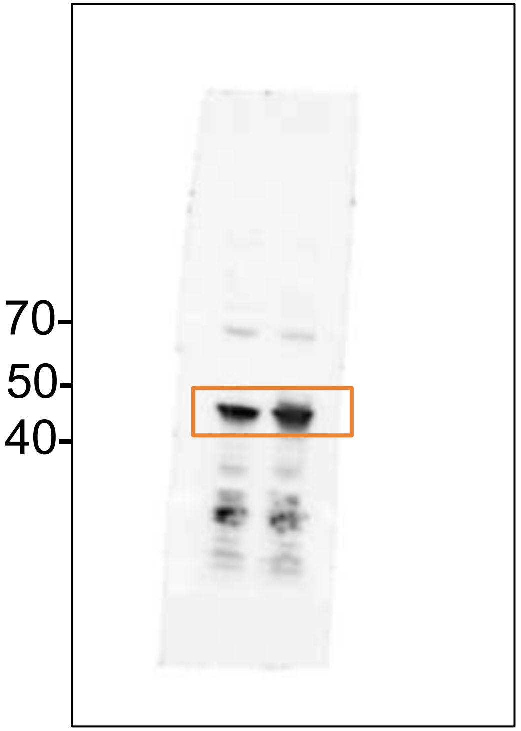

IB:Anti-Actin(42kDa)

input

Fig.1 D used

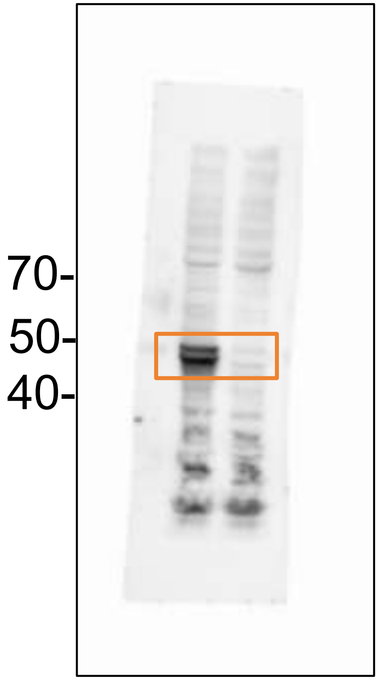

IB:Anti-DNAJA2 (46kDa)

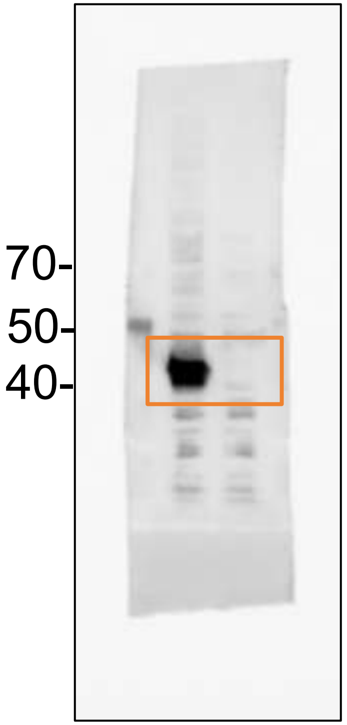

IB:Anti-flag (40kDa)

Ip:Anti-Myc

Fig.1 H used

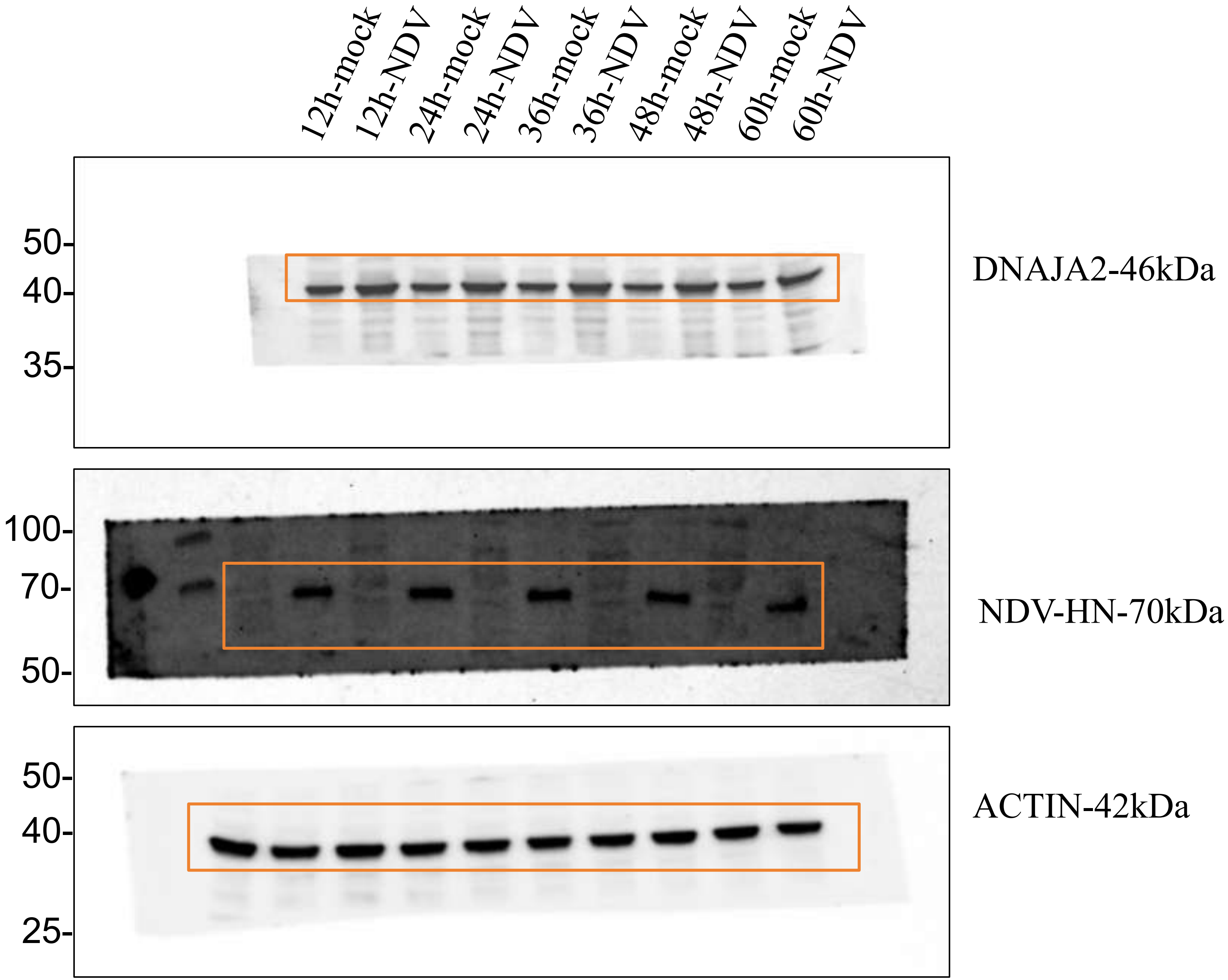
12h-mock
12h-NDV
24h-mock
24h-NDV
36h-mock
36h-NDV
48h-mock
48h-NDV
60h-mock
60h-NDV
50-
40-
35-
DNAJA2-46kDa
100-
70-
50-
NDV-HN-70kDa
50-
40-
25-
ACTIN-42kDa

Fig.1 H Repeat 1

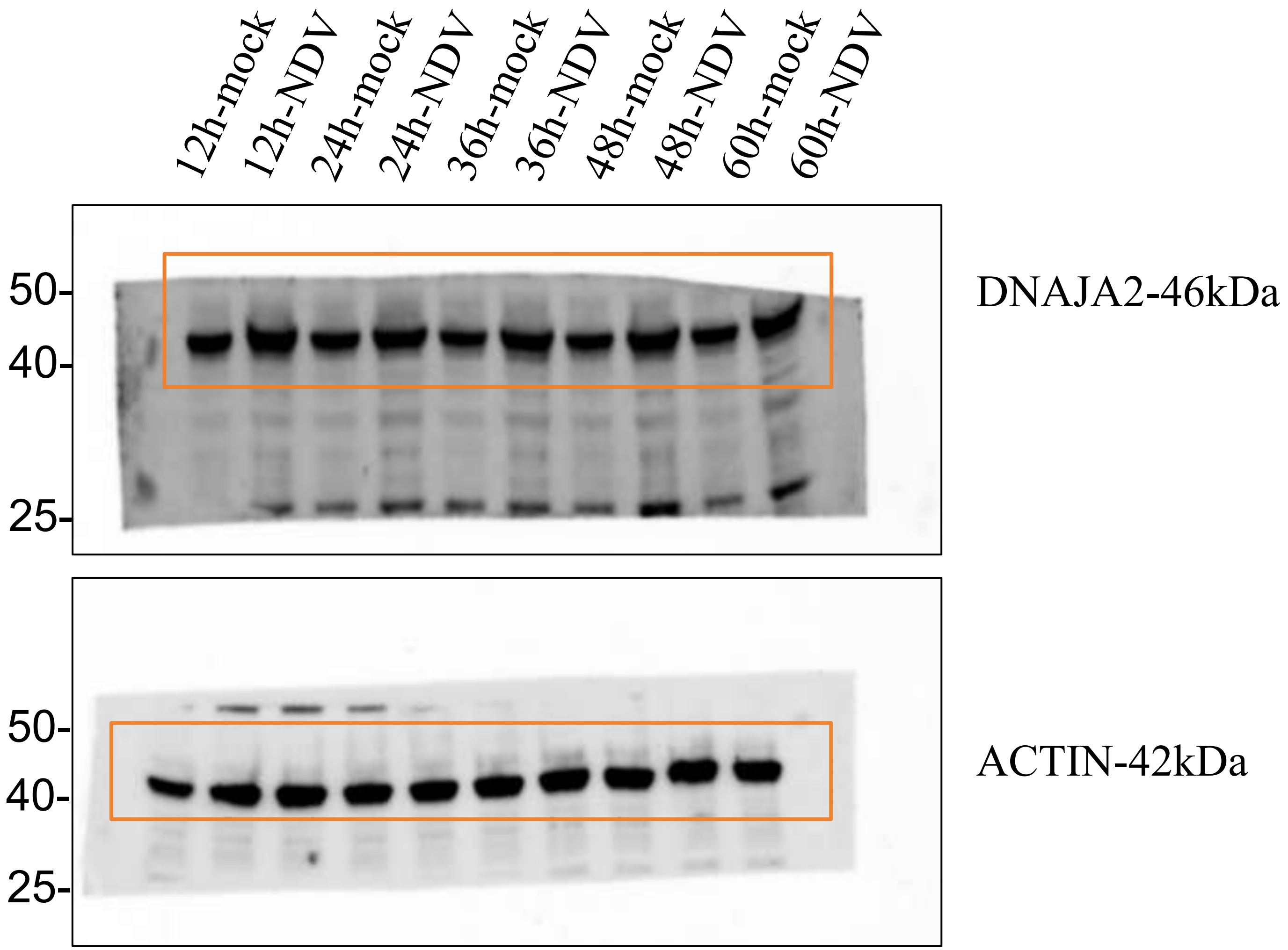

Fig.1 H Repeat 2

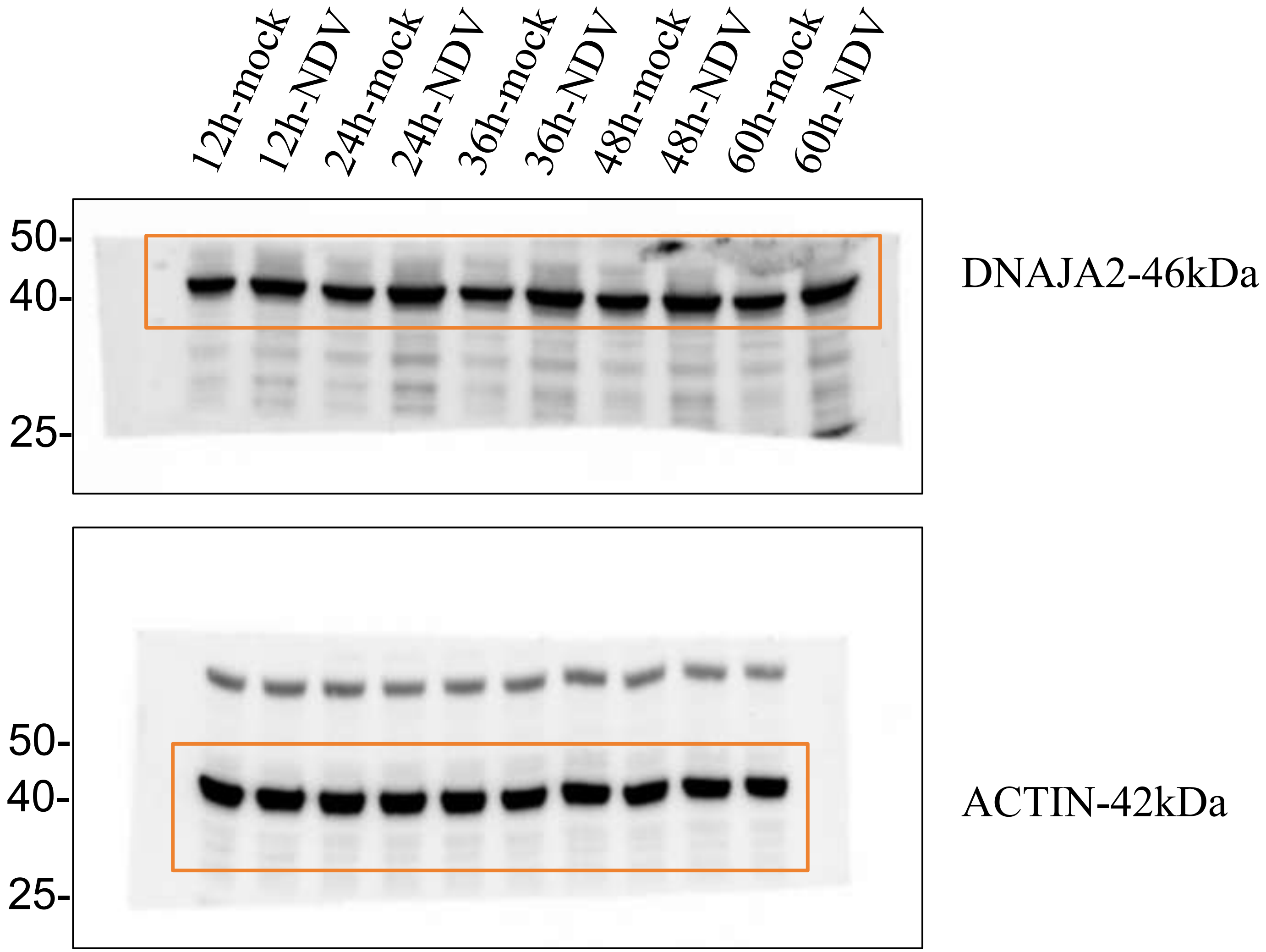

Fig.1 I used

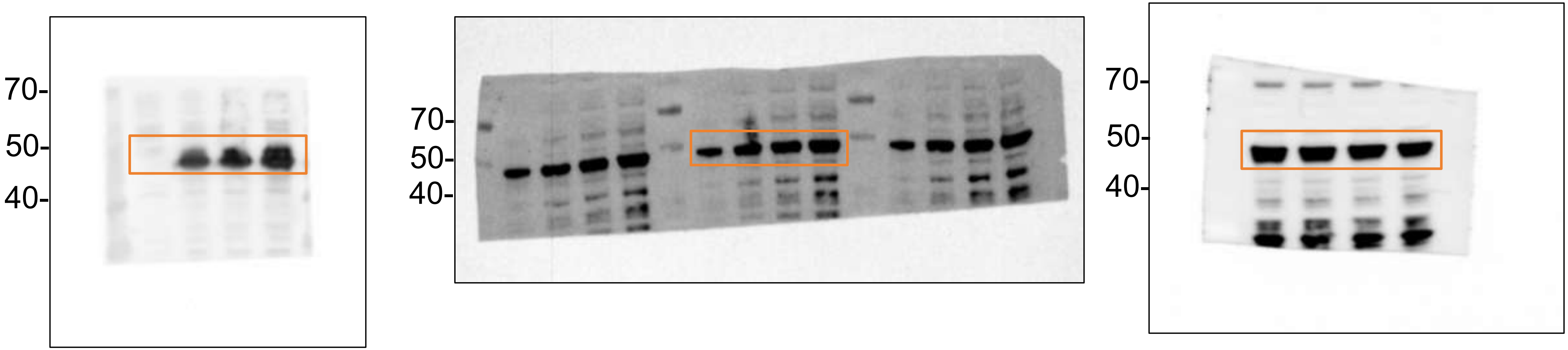

Flag-40kDa DNAJA2-46kDa ACTIN-42kDa

Fig.1 I repeat

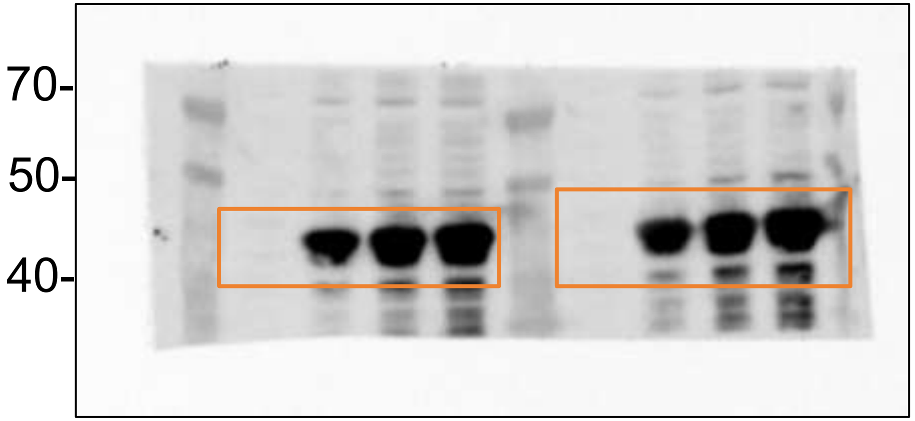

Flag-40kDa

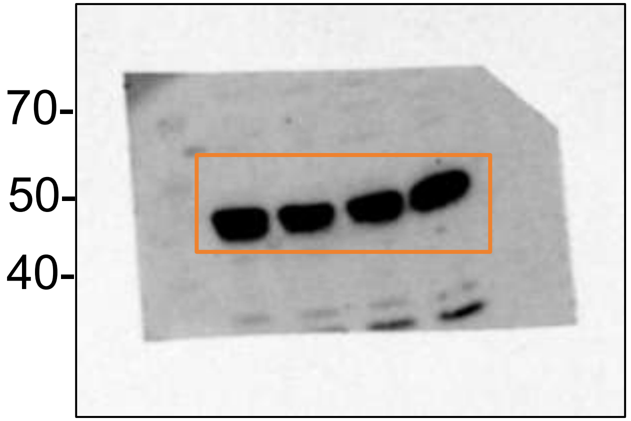

ACTIN-42kDa

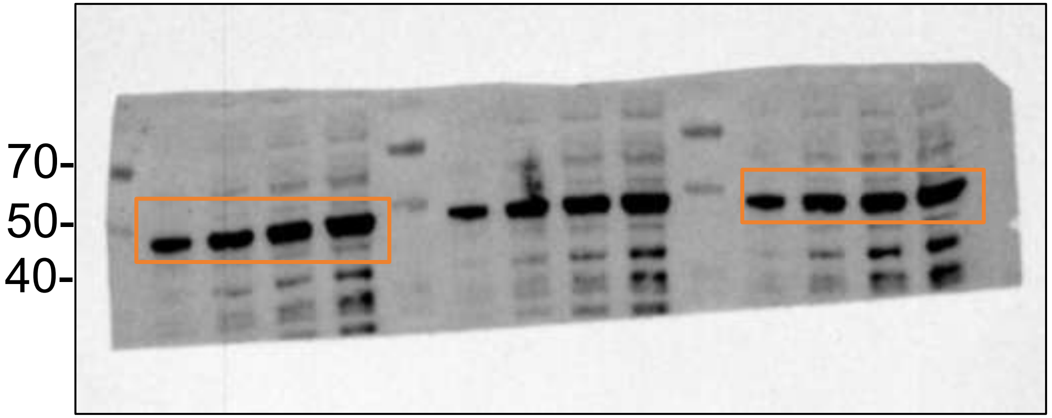

DNAJA2-46kDa

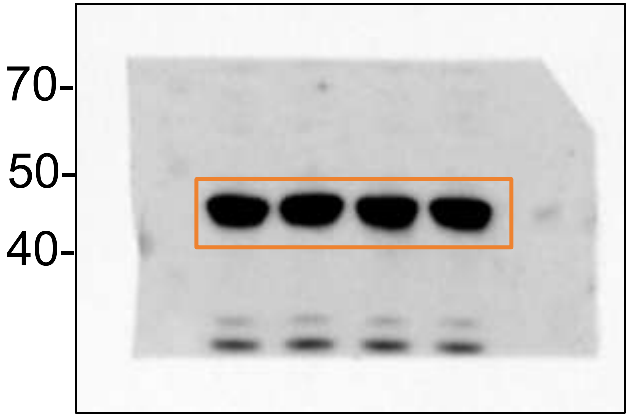

ACTIN-42kDa

Fig.2B used

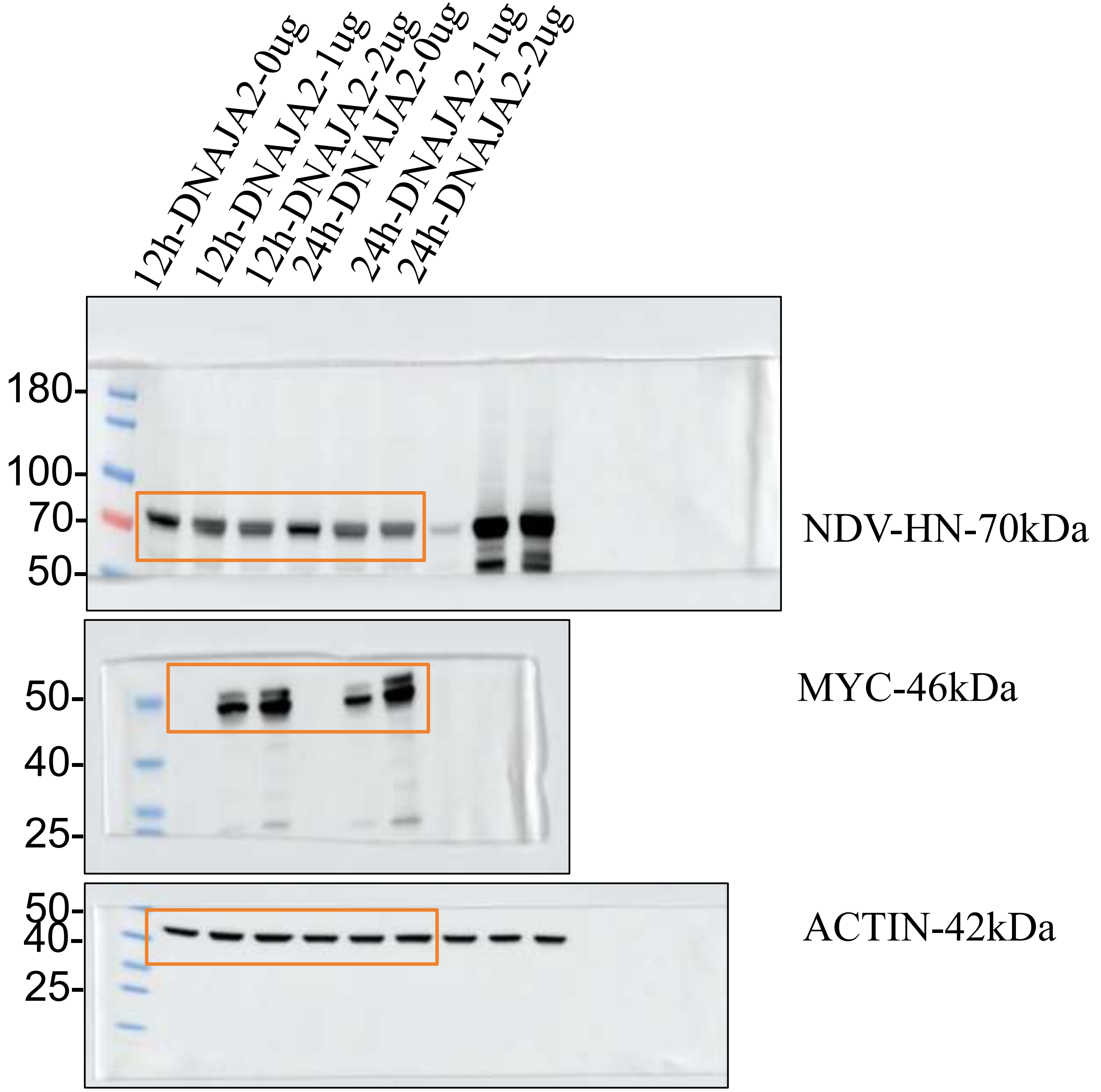

12h-DNAJA2-0ug
12h-DNAJA2-1ug
12h-DNAJA2-2ug
24h-DNAJA2-0ug
24h-DNAJA2-1ug
24h-DNAJA2-2ug
180-
100-
70-
50-
NDV-HN-70kDa
50-
40-
25-
MYC-46kDa
50-
40-
25-
ACTIN-42kDa

Fig.2B Repeat 1

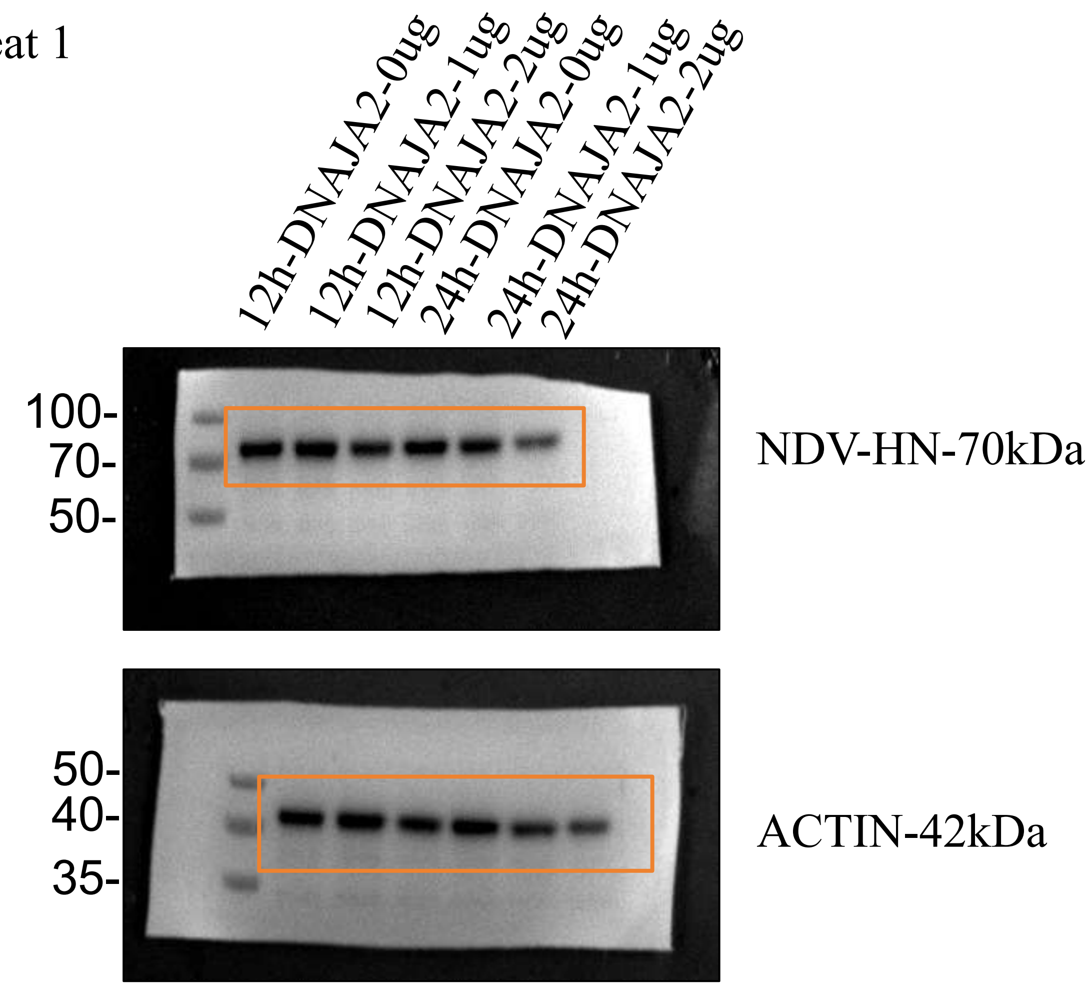

Fig.2B Repeat 2

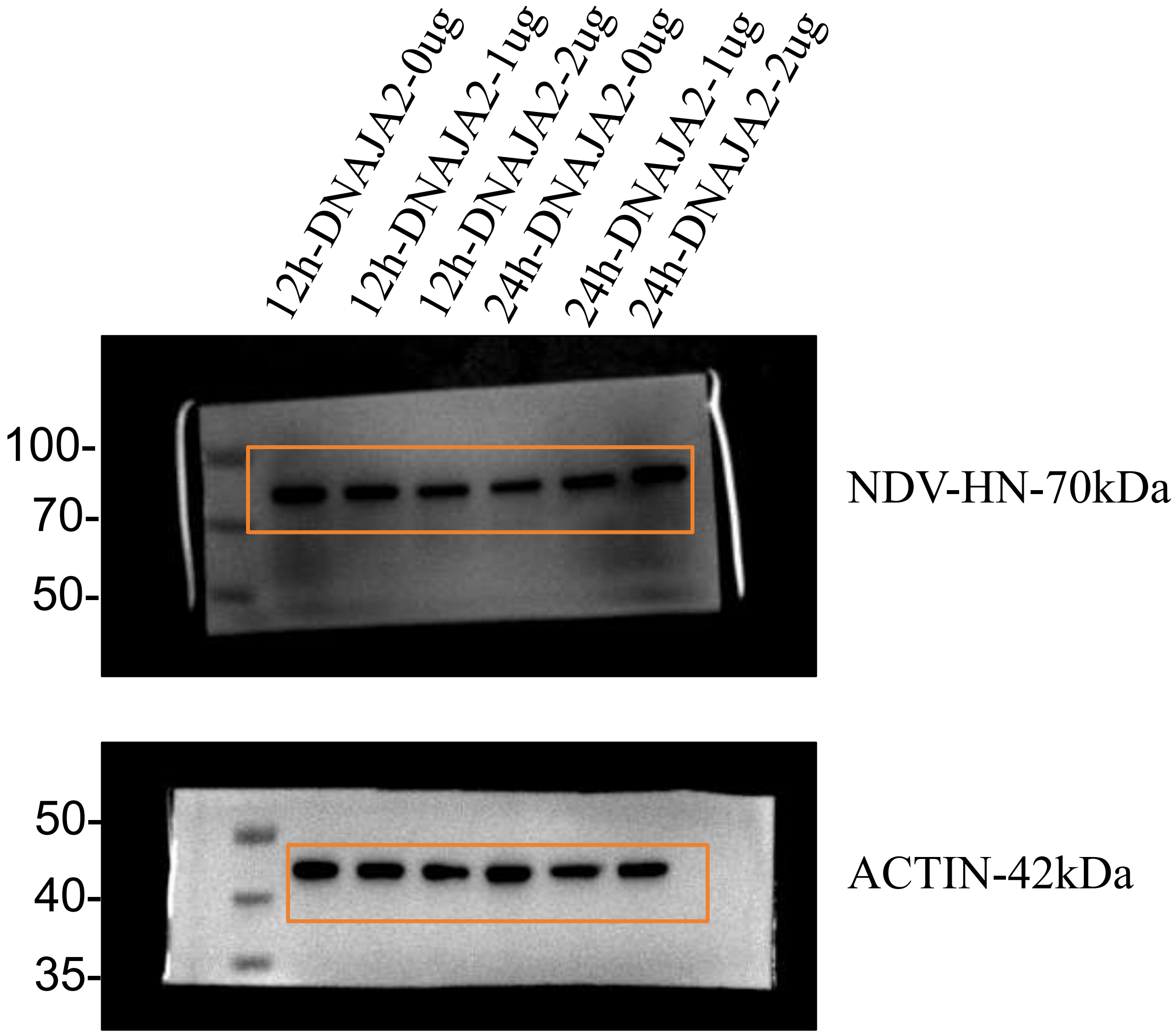

Fig.2F used

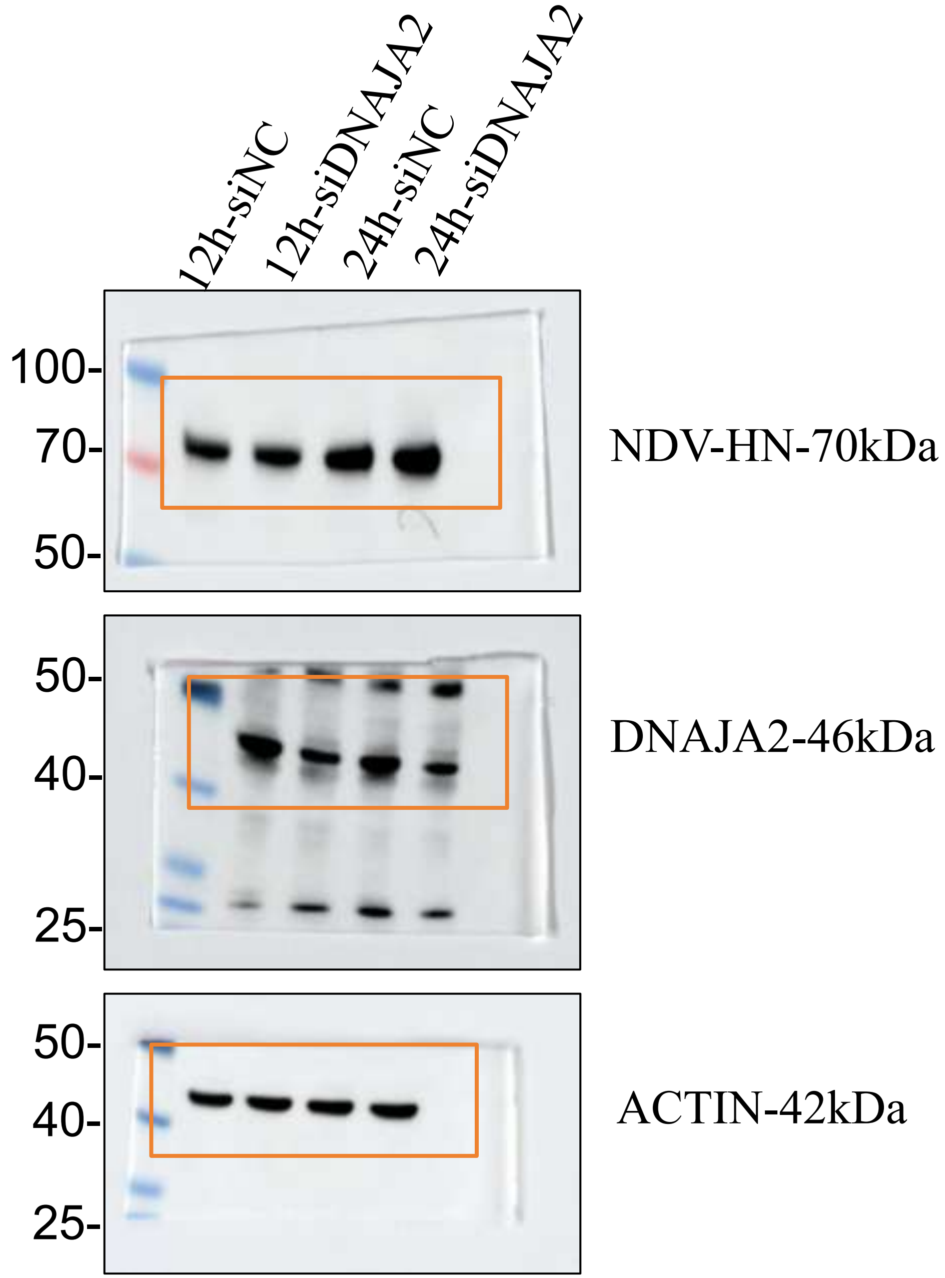

12h-siNC
12h-siDNAJA2
24h-siNC
24h-siDNAJA2
100-
70-
50-
NDV-HN-70kDa
50-
40-
25-
DNAJA2-46kDa
50-
40-
25-
ACTIN-42kDa

Fig.2F Repeat 1 and 2

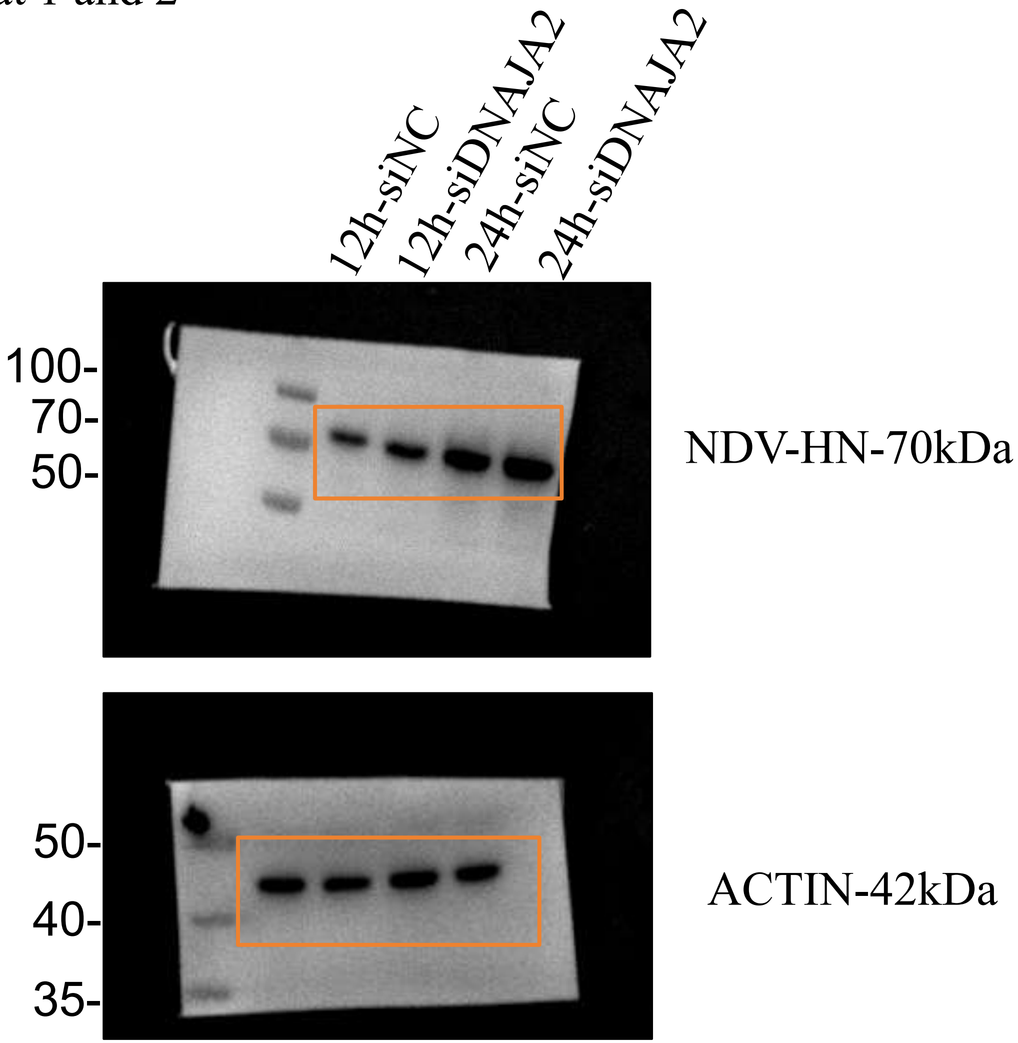

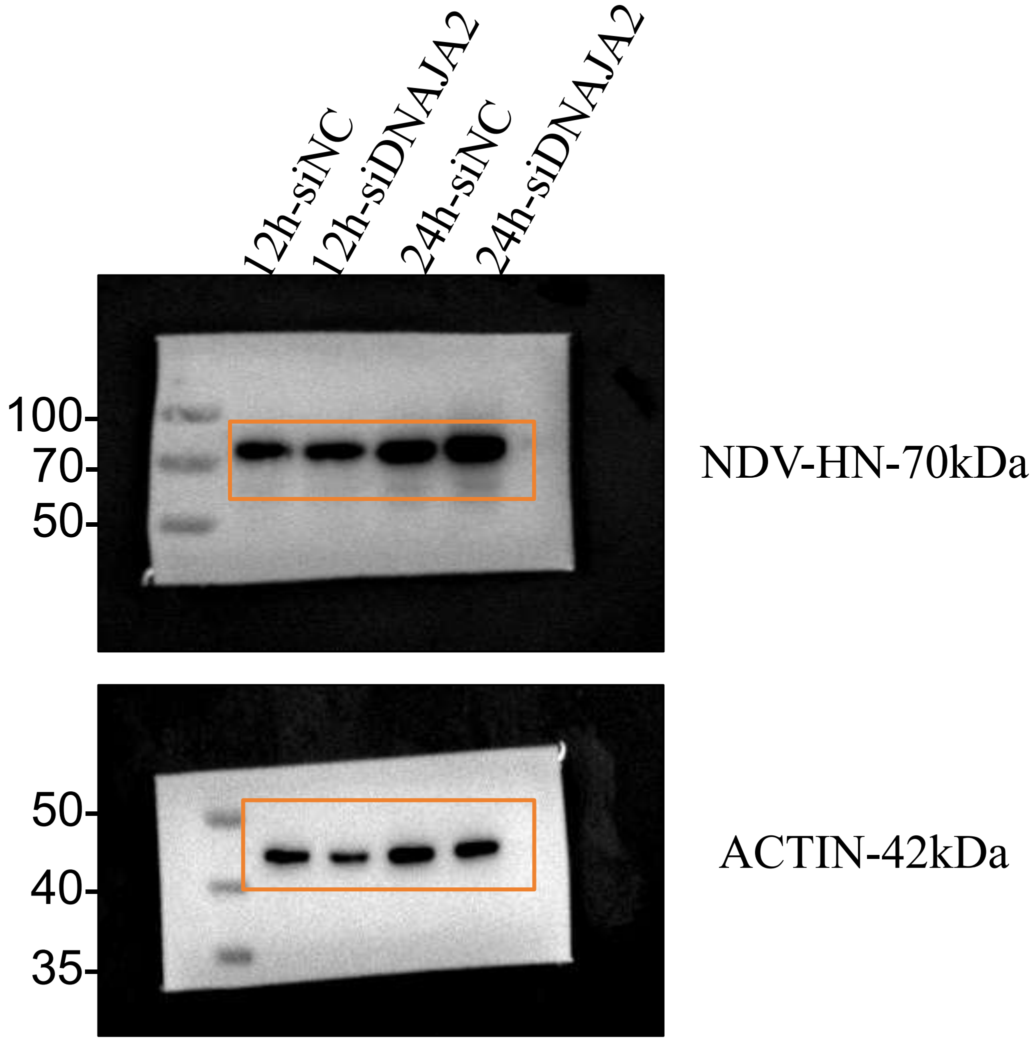

Fig.3A used

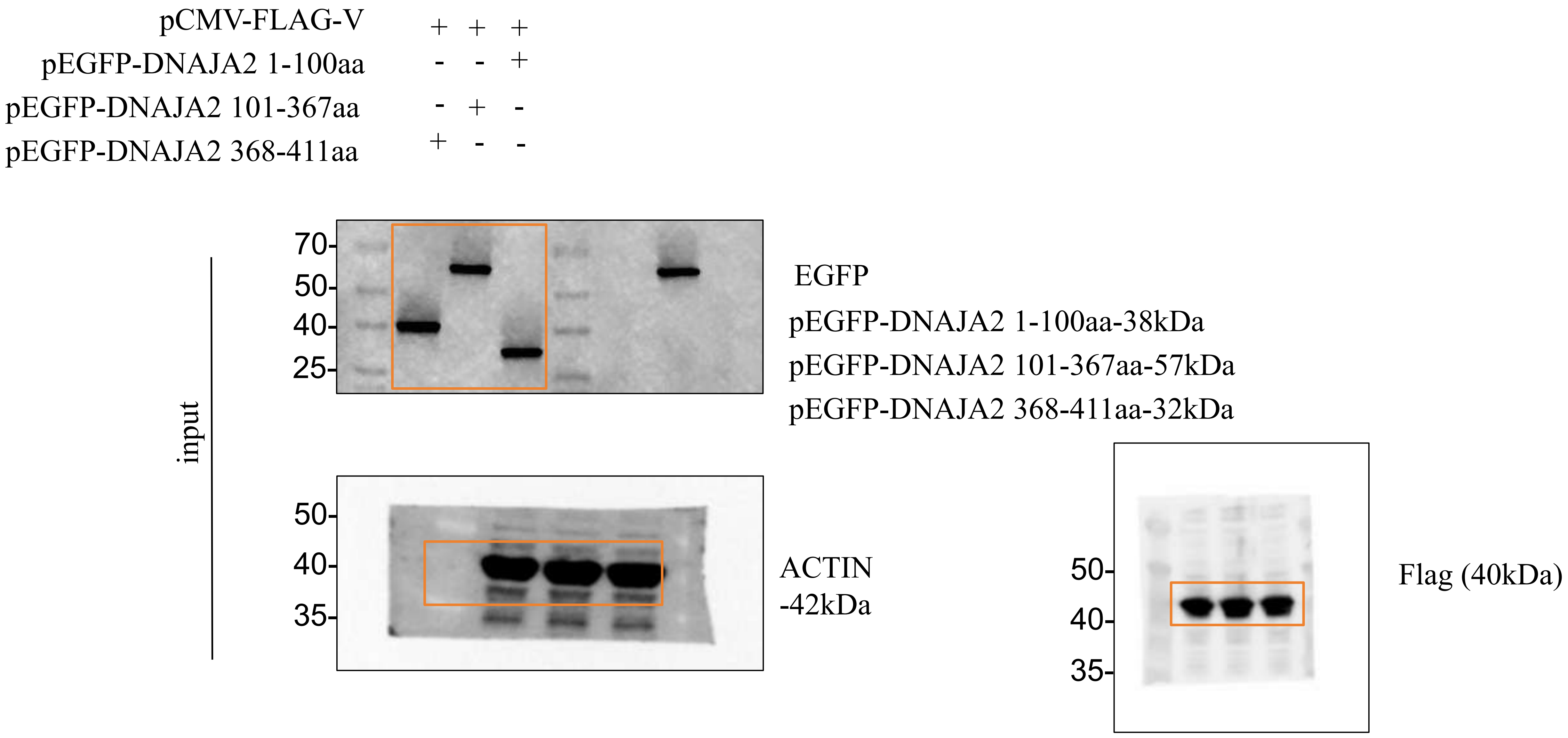

pCMV-FLAG-V + + +
pEGFP-DNAJA2 1-100aa - - +
pEGFP-DNAJA2 101-367aa - + -
pEGFP-DNAJA2 368-411aa + - -
input
70-
50-
40-
25-
EGFP
pEGFP-DNAJA2 1-100aa-38kDa
pEGFP-DNAJA2 101-367aa-57kDa
pEGFP-DNAJA2 368-411aa-32kDa
50-
40-
35-
ACTIN
-42kDa
50-
40-
35-
Flag (40kDa)

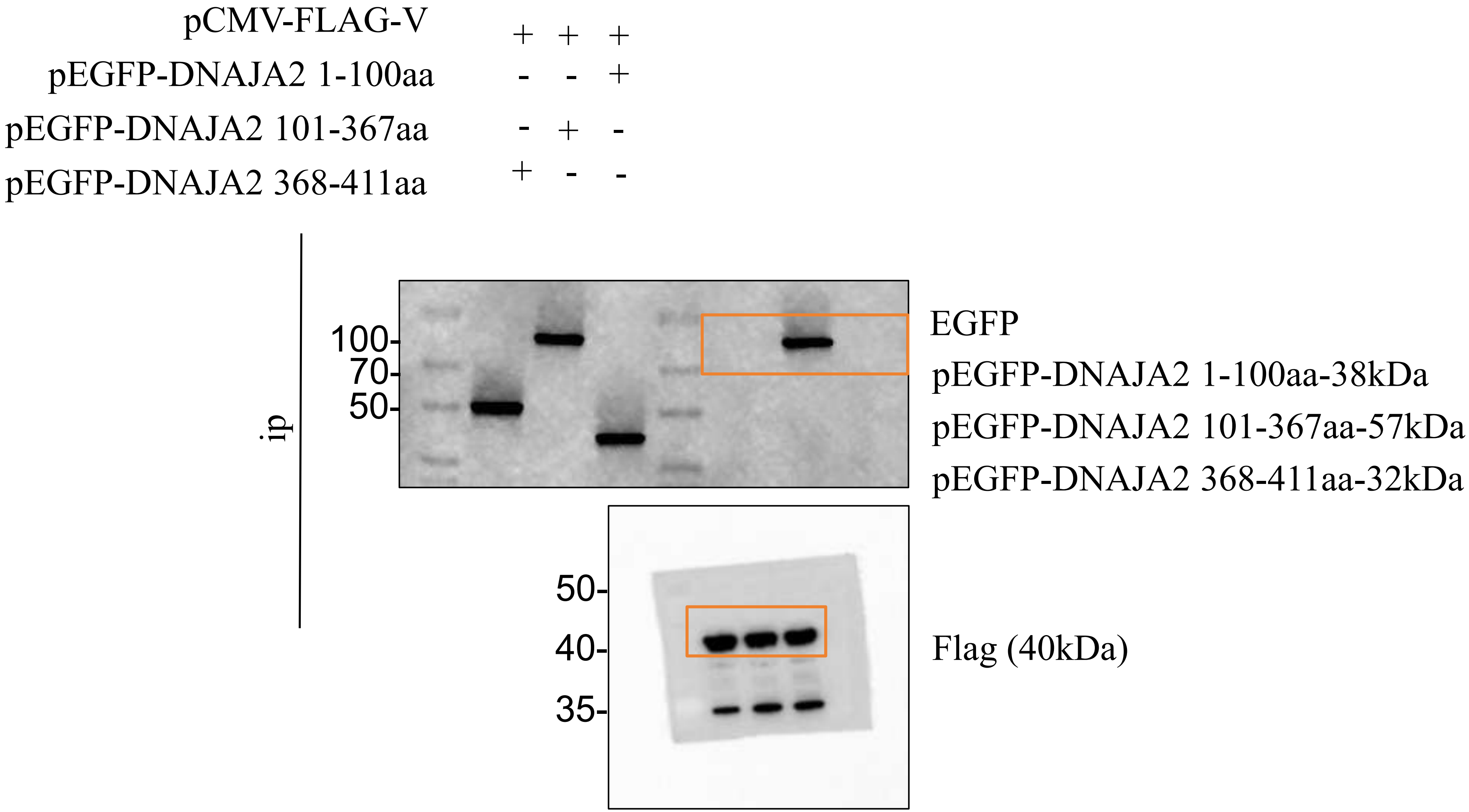
pCMV-FLAG-V + + +
pEGFP-DNAJA2 1-100aa - - +
pEGFP-DNAJA2 101-367aa - + -
pEGFP-DNAJA2 368-411aa + - -
ip
100-
70-
50-
EGFP
pEGFP-DNAJA2 1-100aa-38kDa
pEGFP-DNAJA2 101-367aa-57kDa
pEGFP-DNAJA2 368-411aa-32kDa
50-
40-
35-
Flag (40kDa)

Fig.3C used

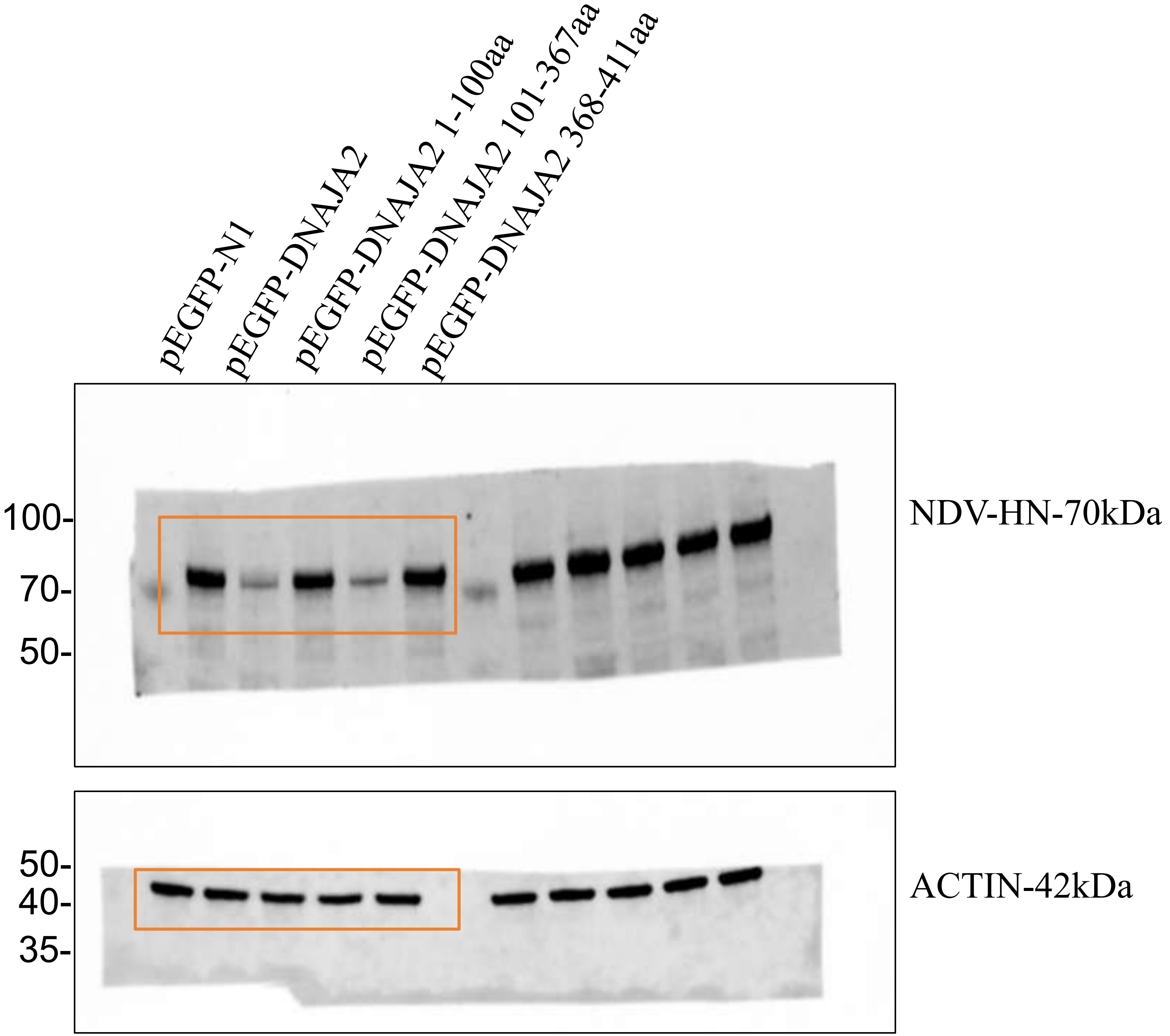

pEGFP-N1
pEGFP-DNAJA2
pEGFP-DNAJA2 1-100aa
pEGFP-DNAJA2 101-367aa
pEGFP-DNAJA2 368-411aa
100-
70-
50-
NDV-HN-70kDa
50-
40-
35-
ACTIN-42kDa

Fig.3C Repeat 1

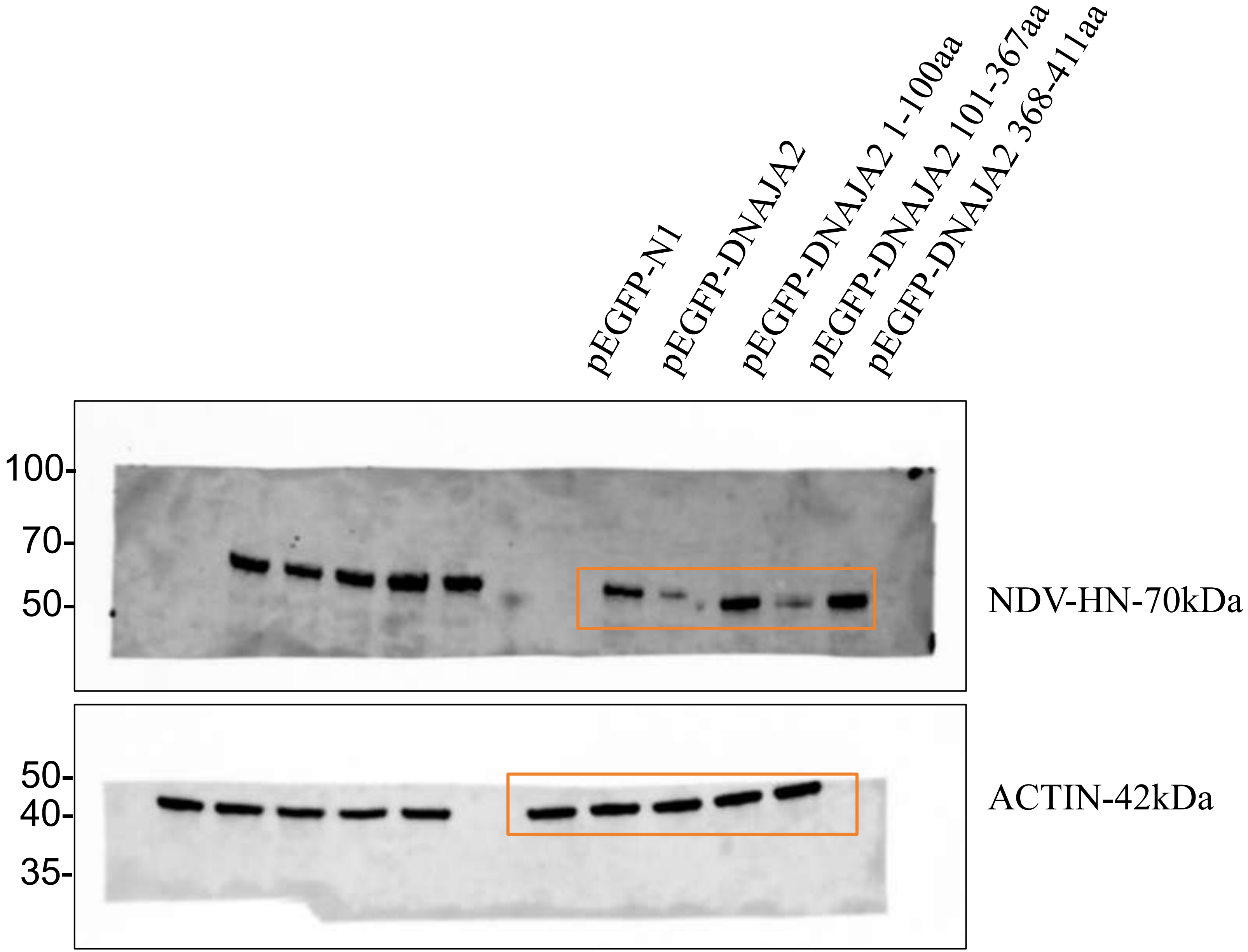

Fig.3C Repeat 2

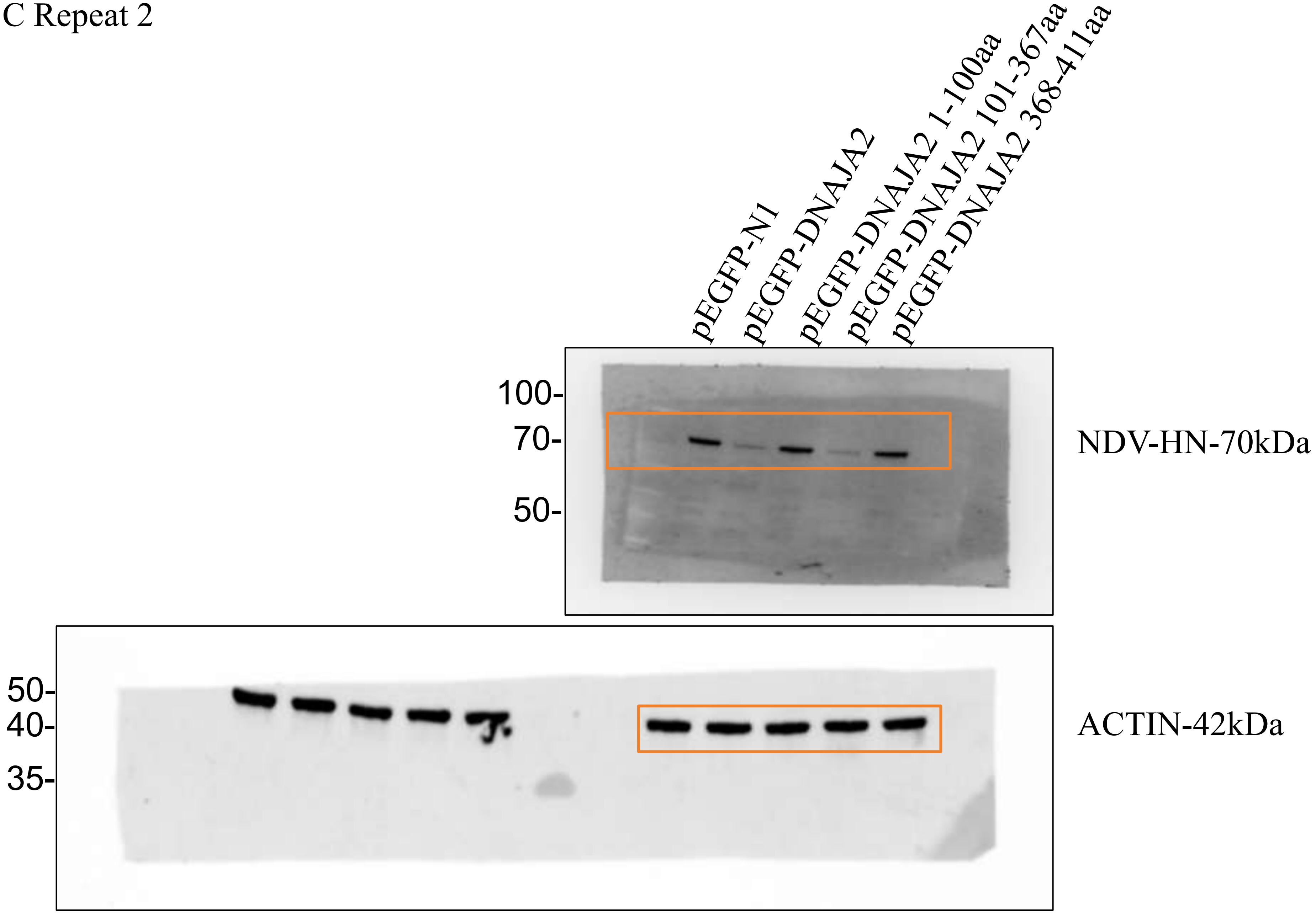

Fig.4I used

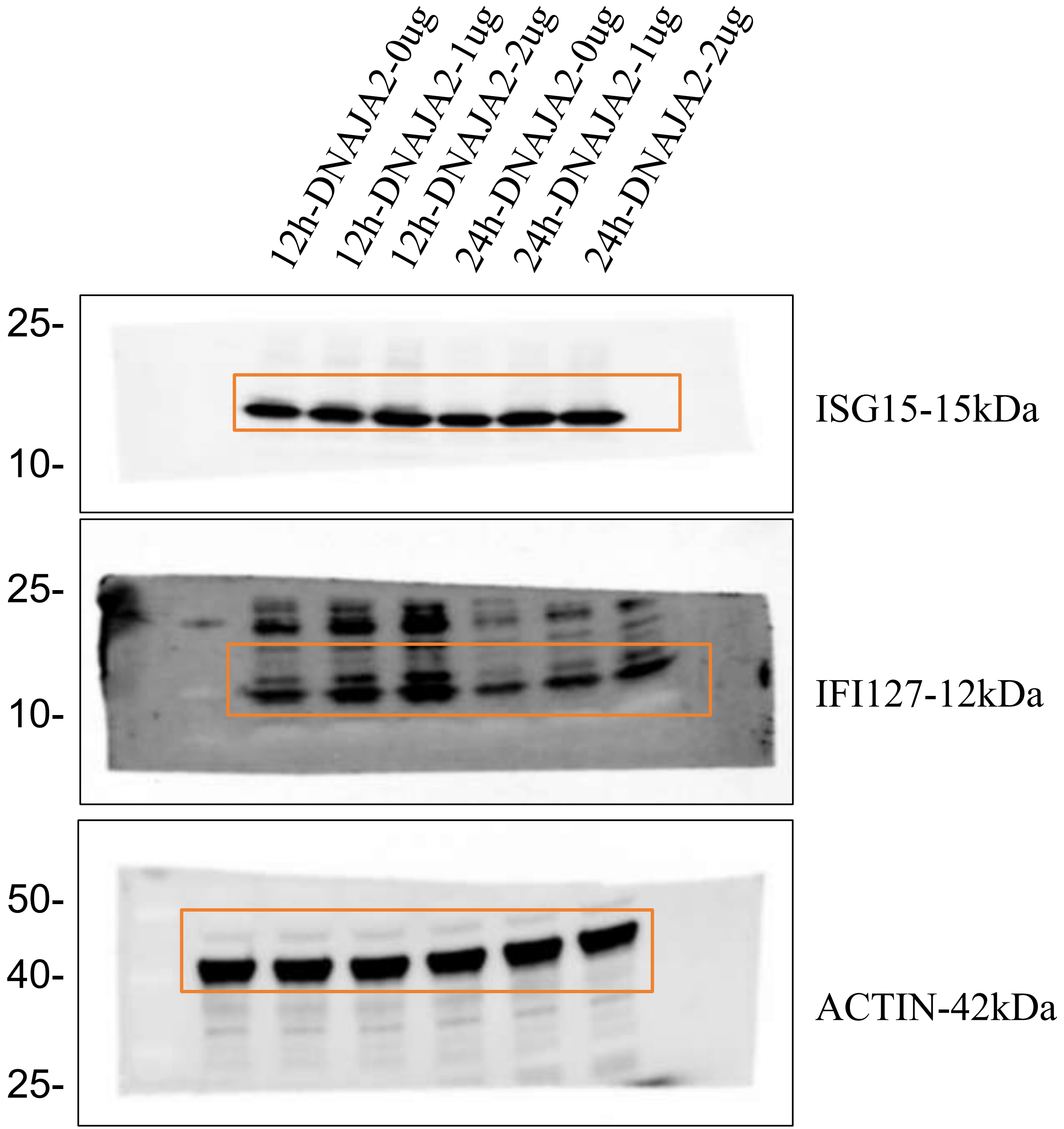
12h-DNAJA2-0ug
12h-DNAJA2-1ug
12h-DNAJA2-2ug
24h-DNAJA2-0ug
24h-DNAJA2-1ug
24h-DNAJA2-2ug
25-
10-
ISG15-15kDa
25-
10-
IFI127-12kDa
50-
40-
25-
ACTIN-42kDa

Fig.4I Repeat 1

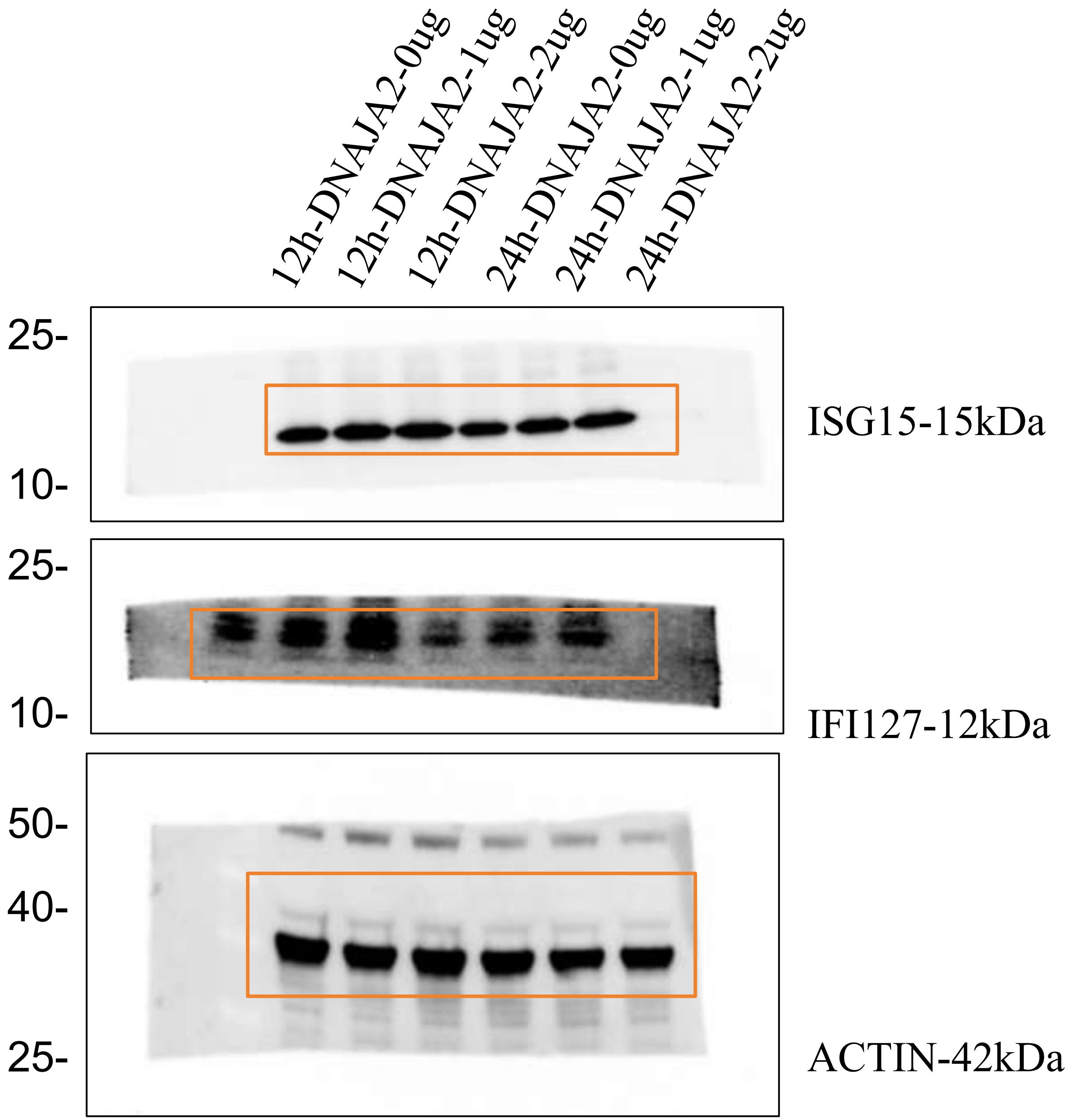

Fig.4I Repeat 2

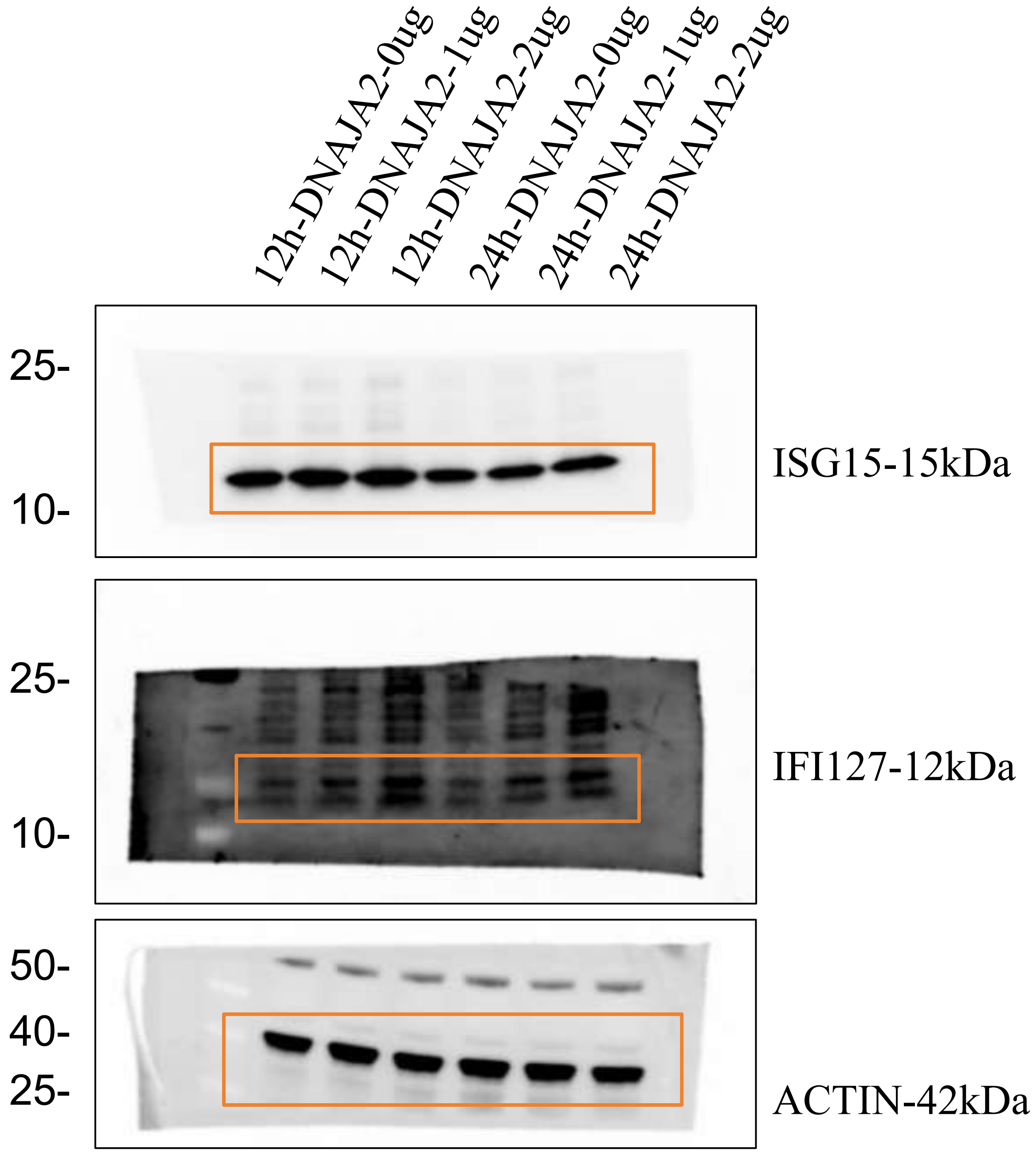

Fig.4R used

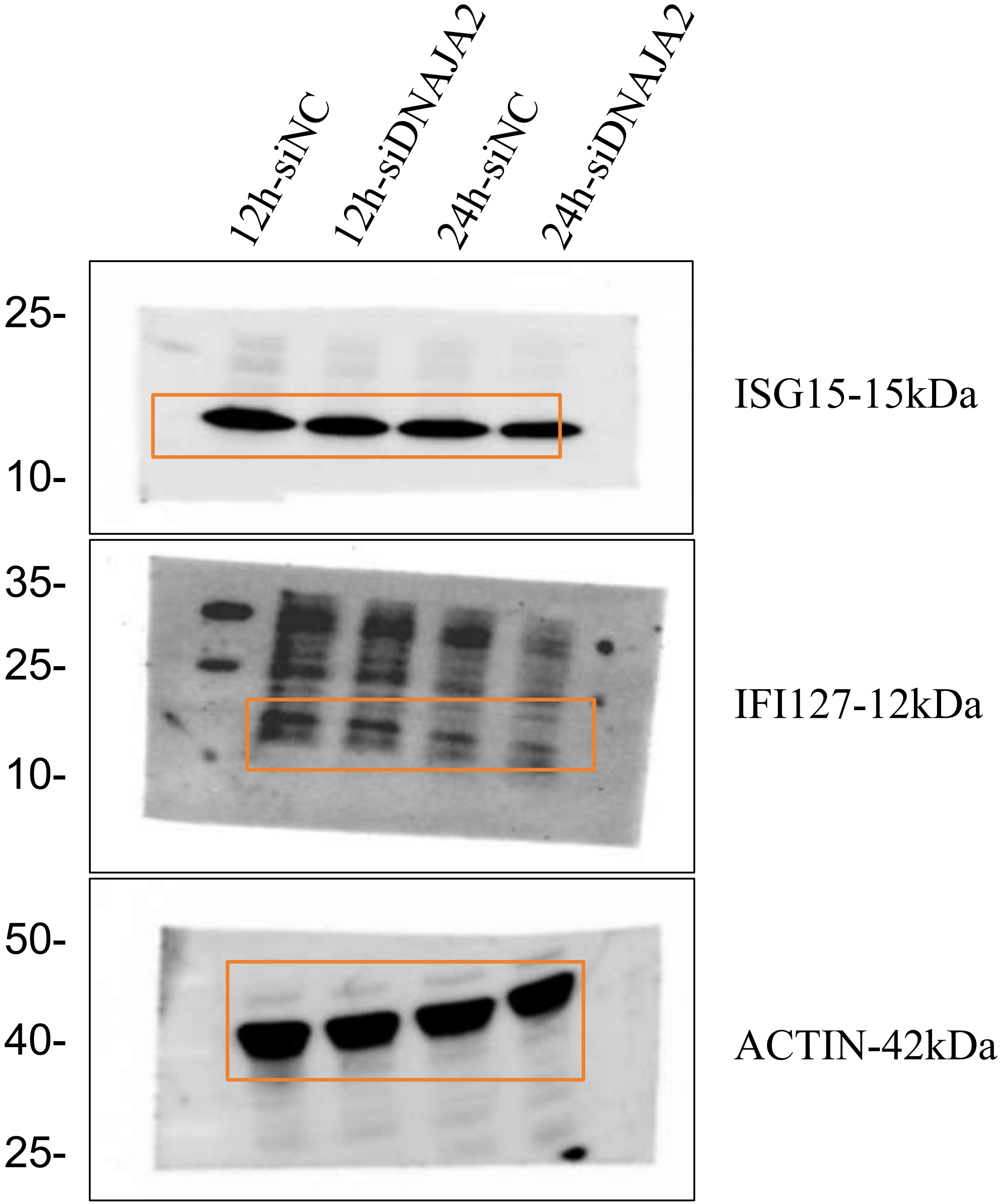
12h-siNC
12h-siDNAJA2
24h-siNC
24h-siDNAJA2
25-
10-
ISG15-15kDa
35-
25-
10-
IFI127-12kDa
50-
40-
25-
ACTIN-42kDa

Fig.4R Repeat 1

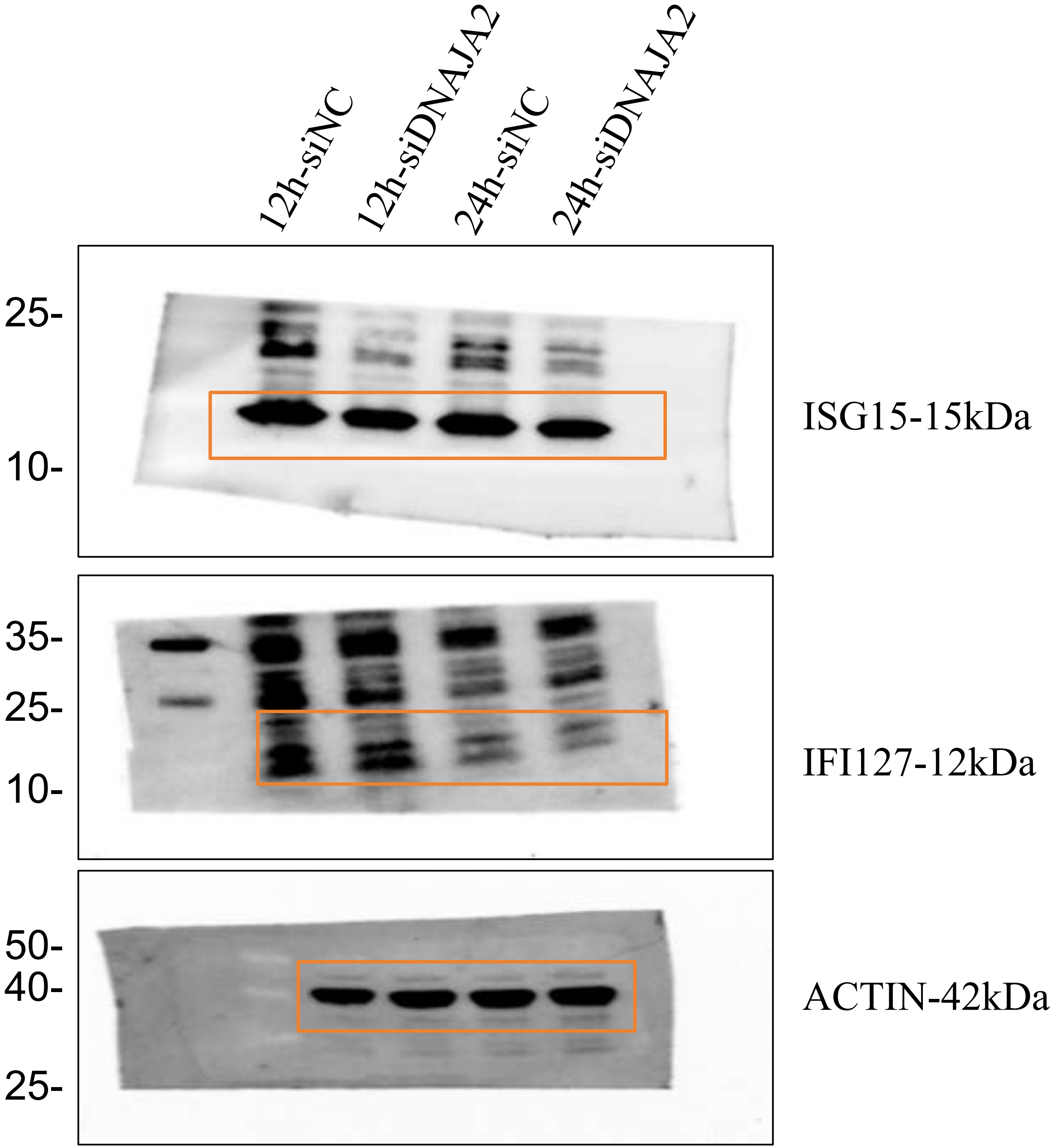

Fig.4R Repeat 2

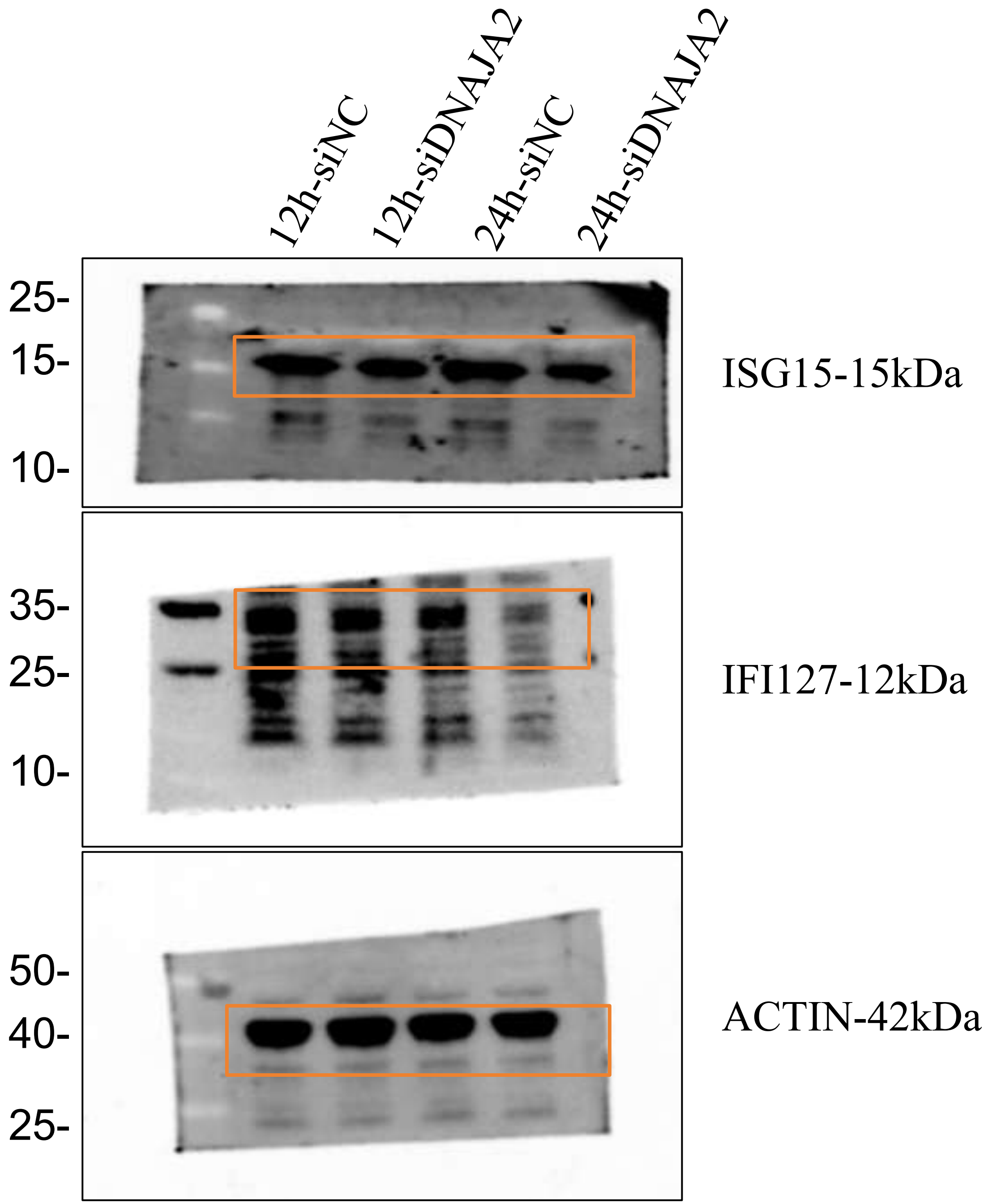

Fig.5C used

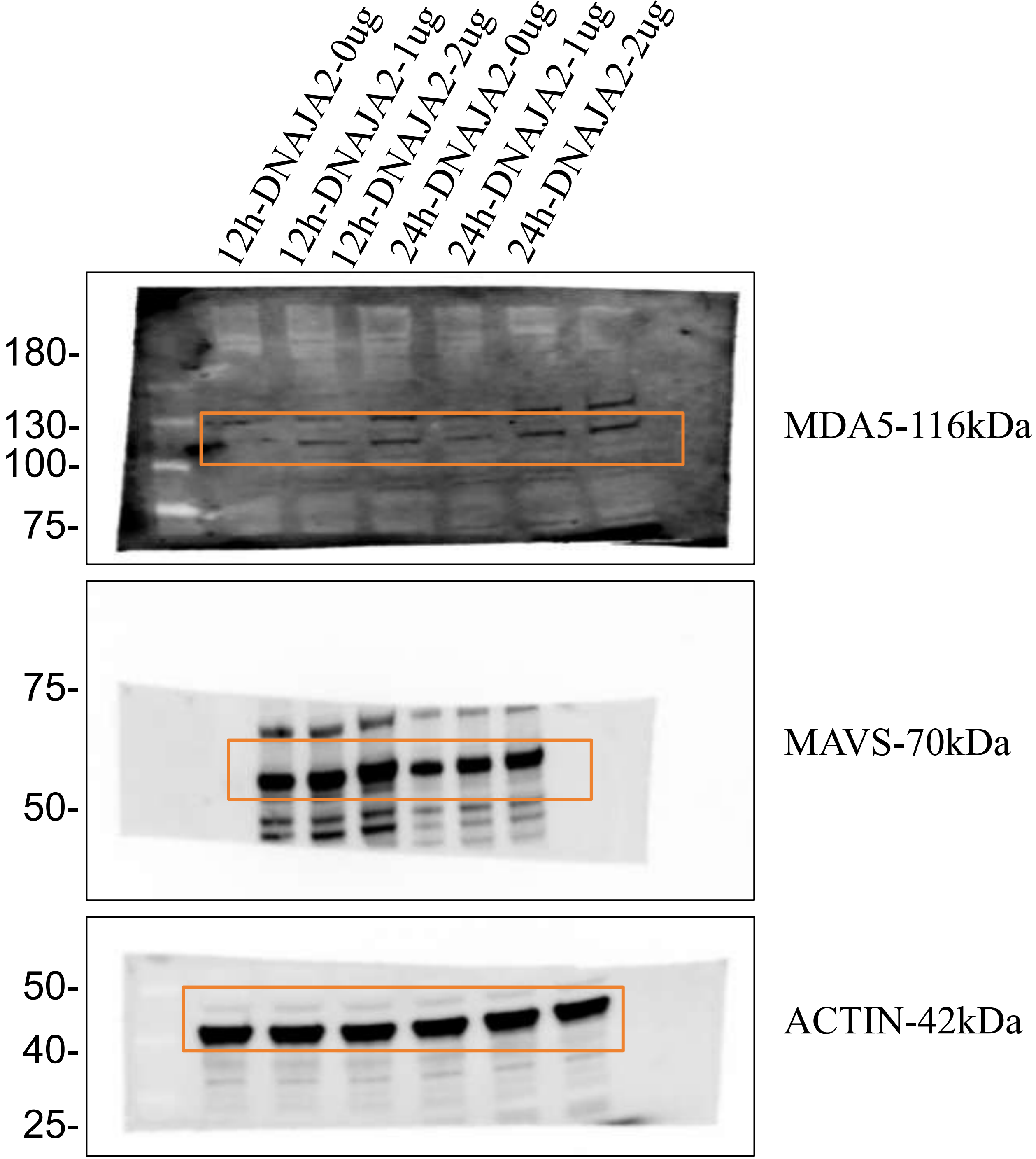

12h-DNAJA2-0ug
12h-DNAJA2-1ug
12h-DNAJA2-2ug
24h-DNAJA2-0ug
24h-DNAJA2-1ug
24h-DNAJA2-2ug
180-
130-
100-
75-
MDA5-116kDa
75-
50-
MAVS-70kDa
50-
40-
25-
ACTIN-42kDa

Fig.5C Repeat 1

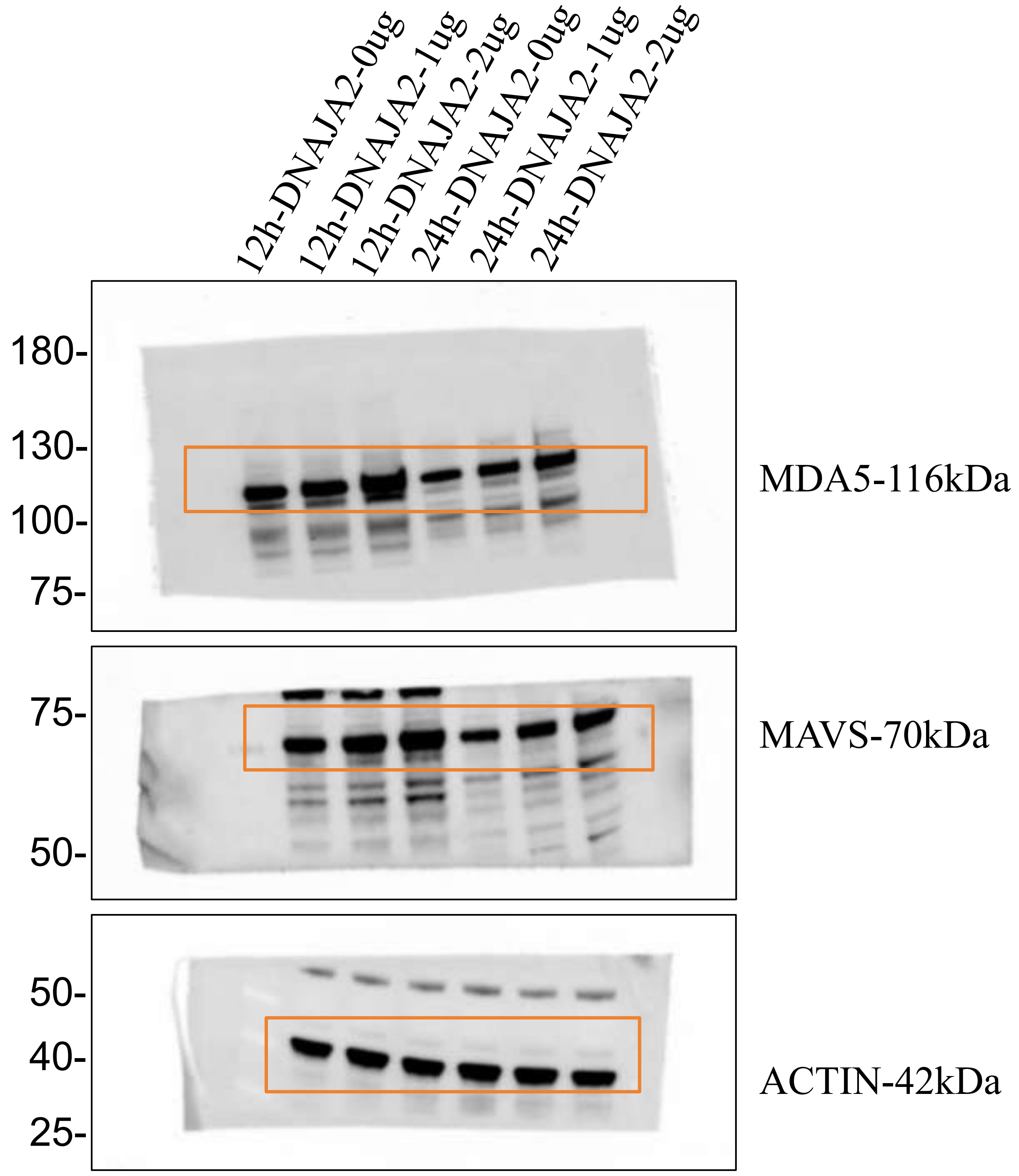

Fig.5C Repeat 2

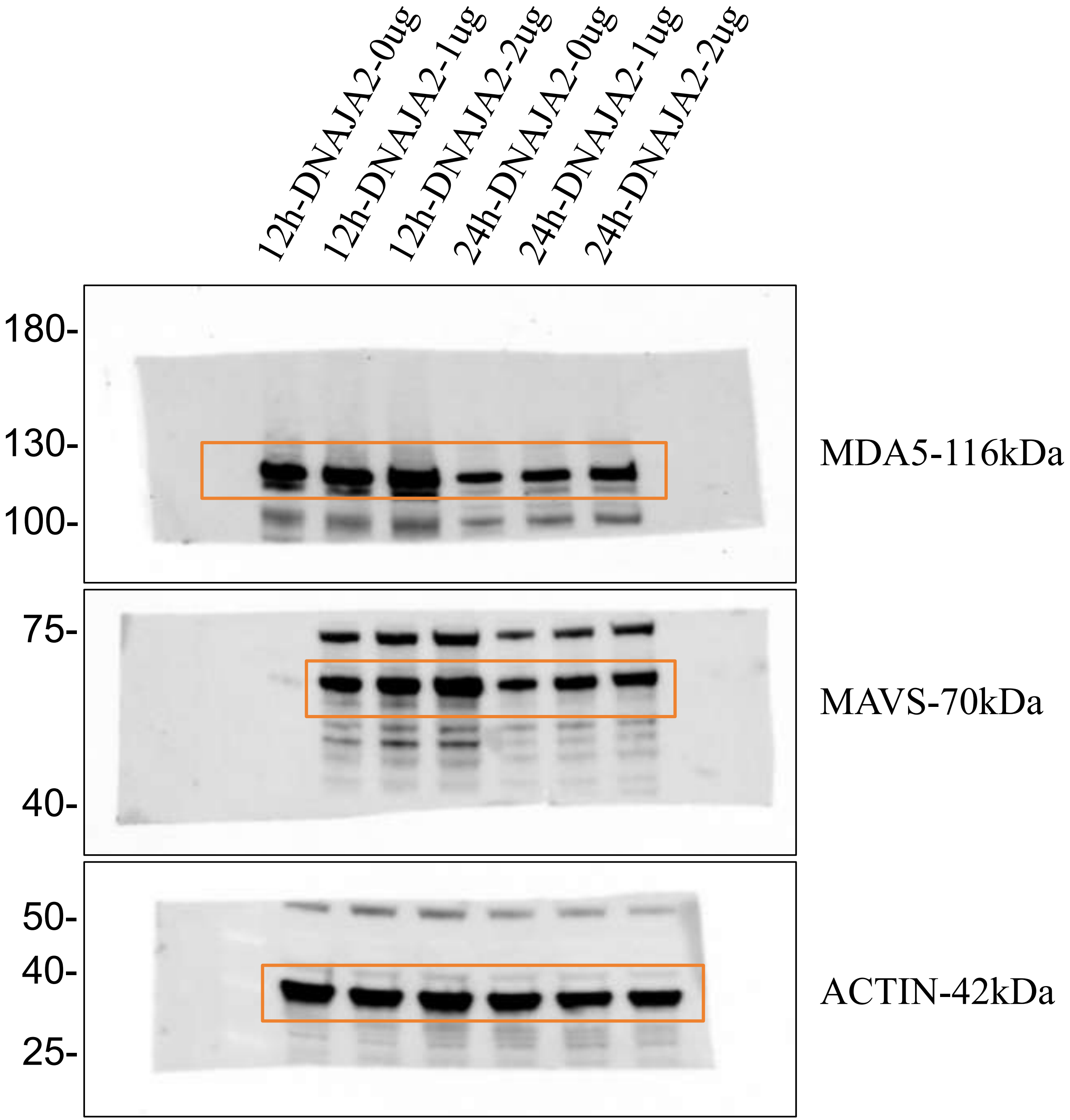

Fig.5D used

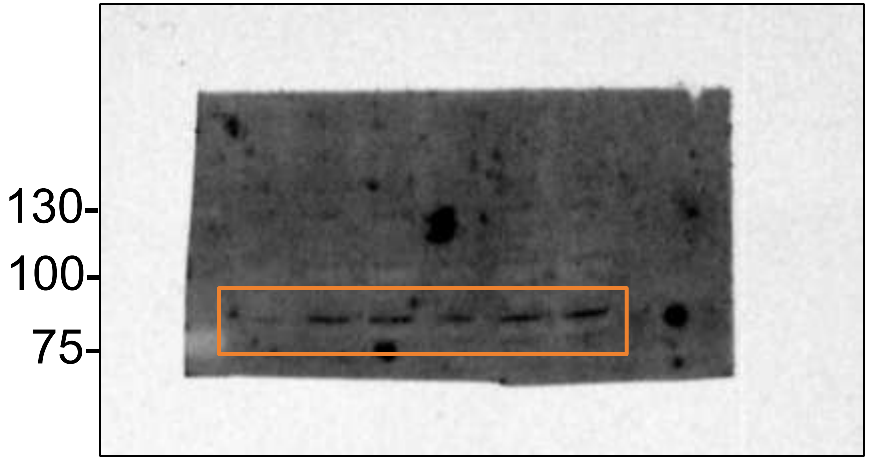

TBK1-86kDa

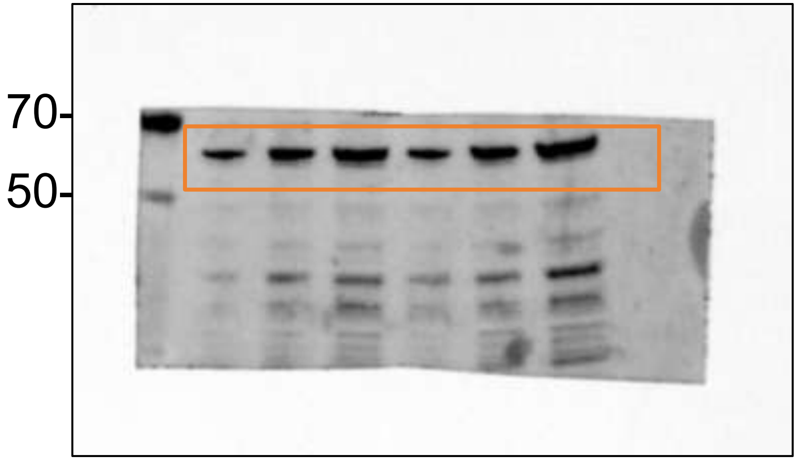

P-IRF3-56kDa

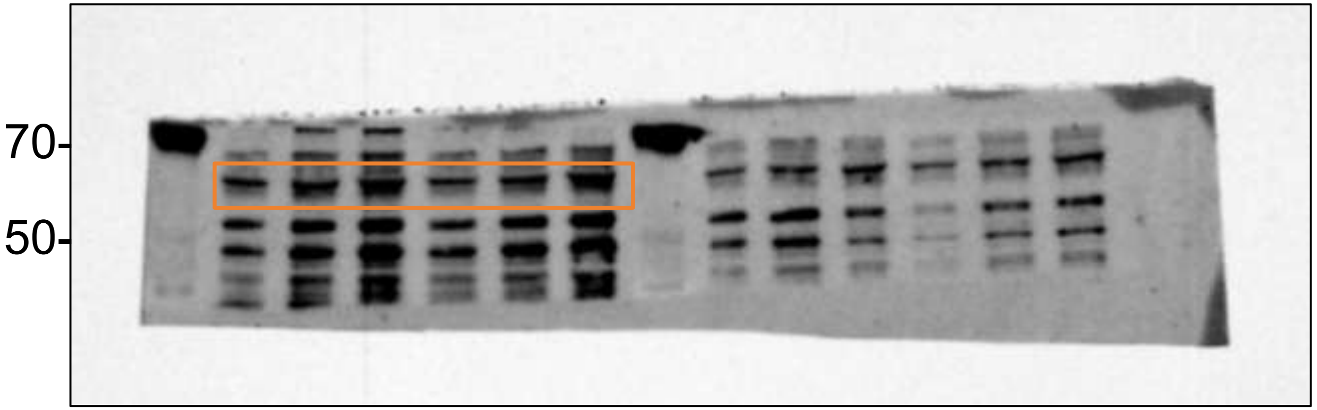

IRF3-56kDa

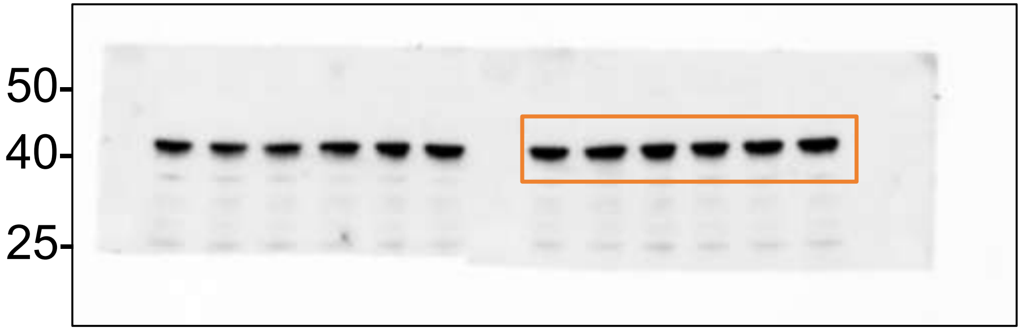

ACTIN-42kDa

Fig.5D repeat

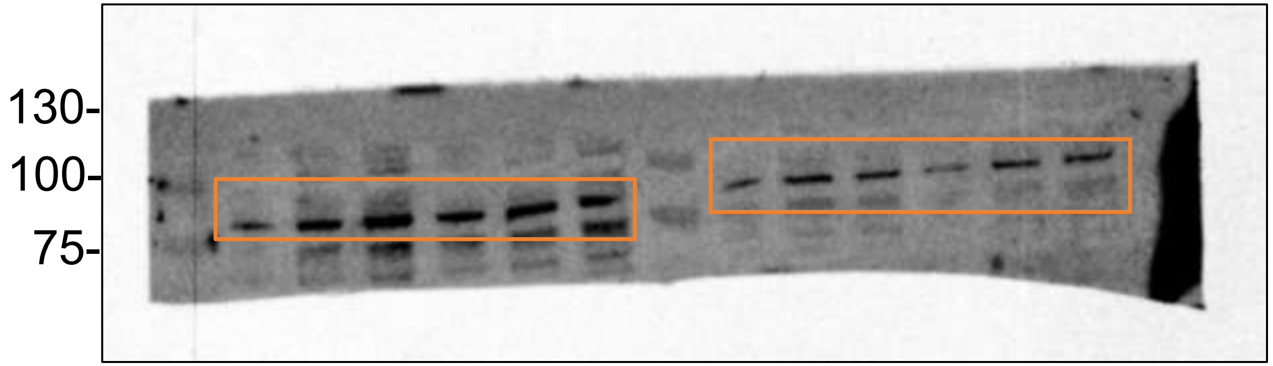

TBK1-86kDa

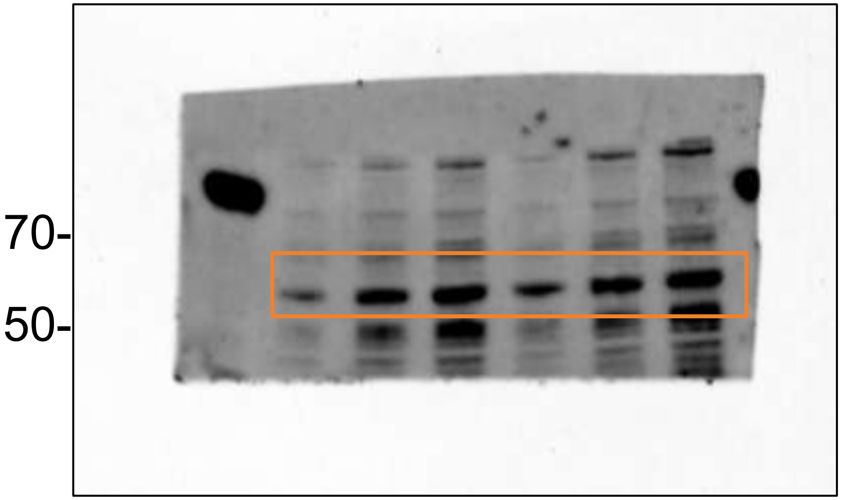

IRF3-56kDa

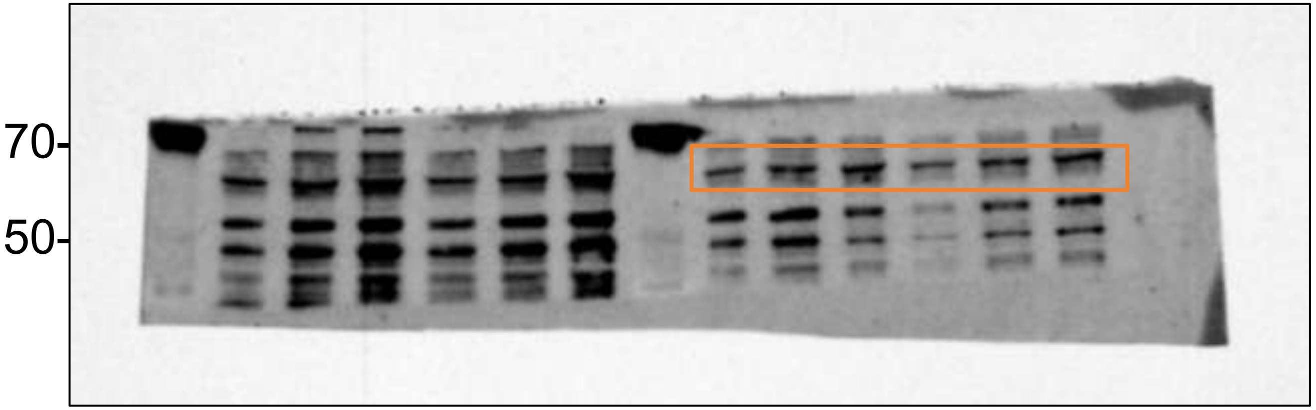

IRF3-56kDa

Fig.5D repeat

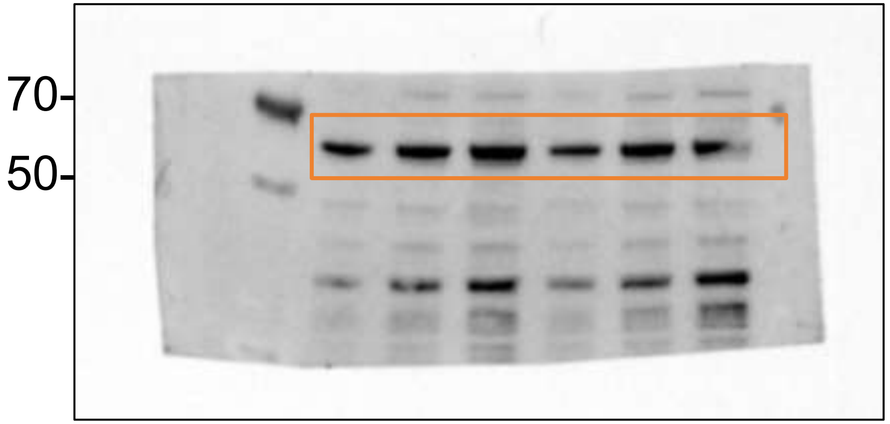

P-IRF3-56kDa

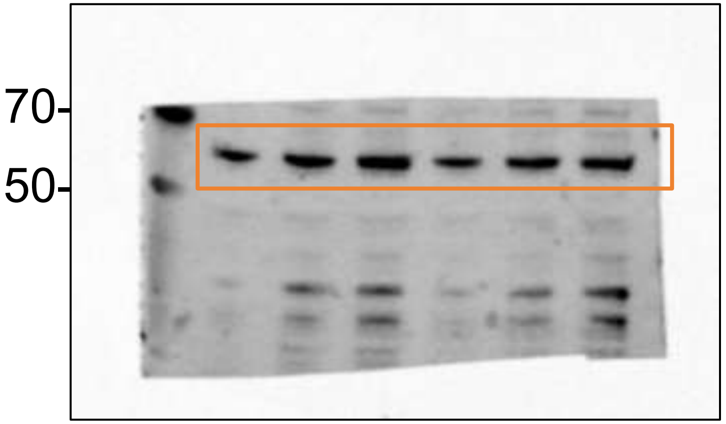

P-IRF3-56kDa

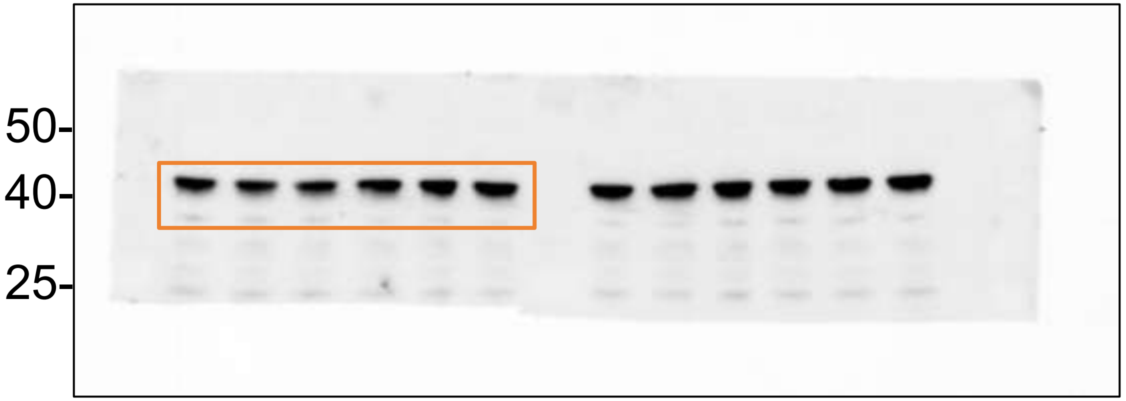

ACTIN-42kDa

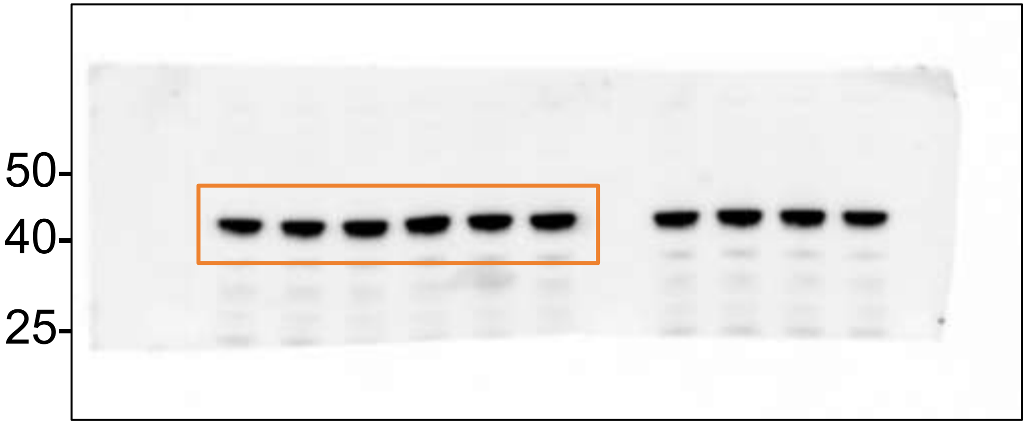

ACTIN-42kDa

Fig.5G used

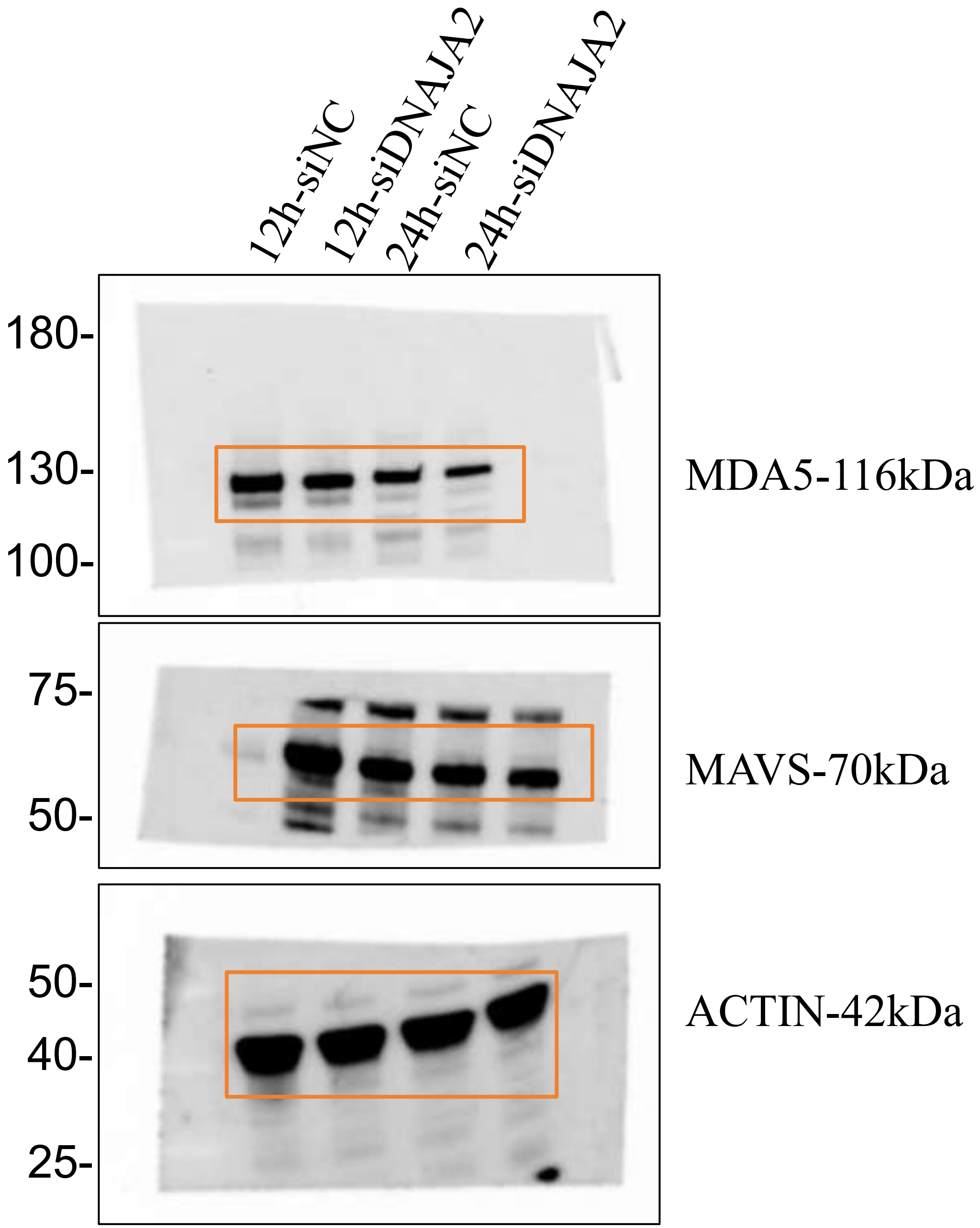

12h-siNC
12h-siDNAJA2
24h-siNC
24h-siDNAJA2
180-
130-
100-
MDA5-116kDa
75-
50-
MAVS-70kDa
50-
40-
25-
ACTIN-42kDa

Fig.5G Repeat 1

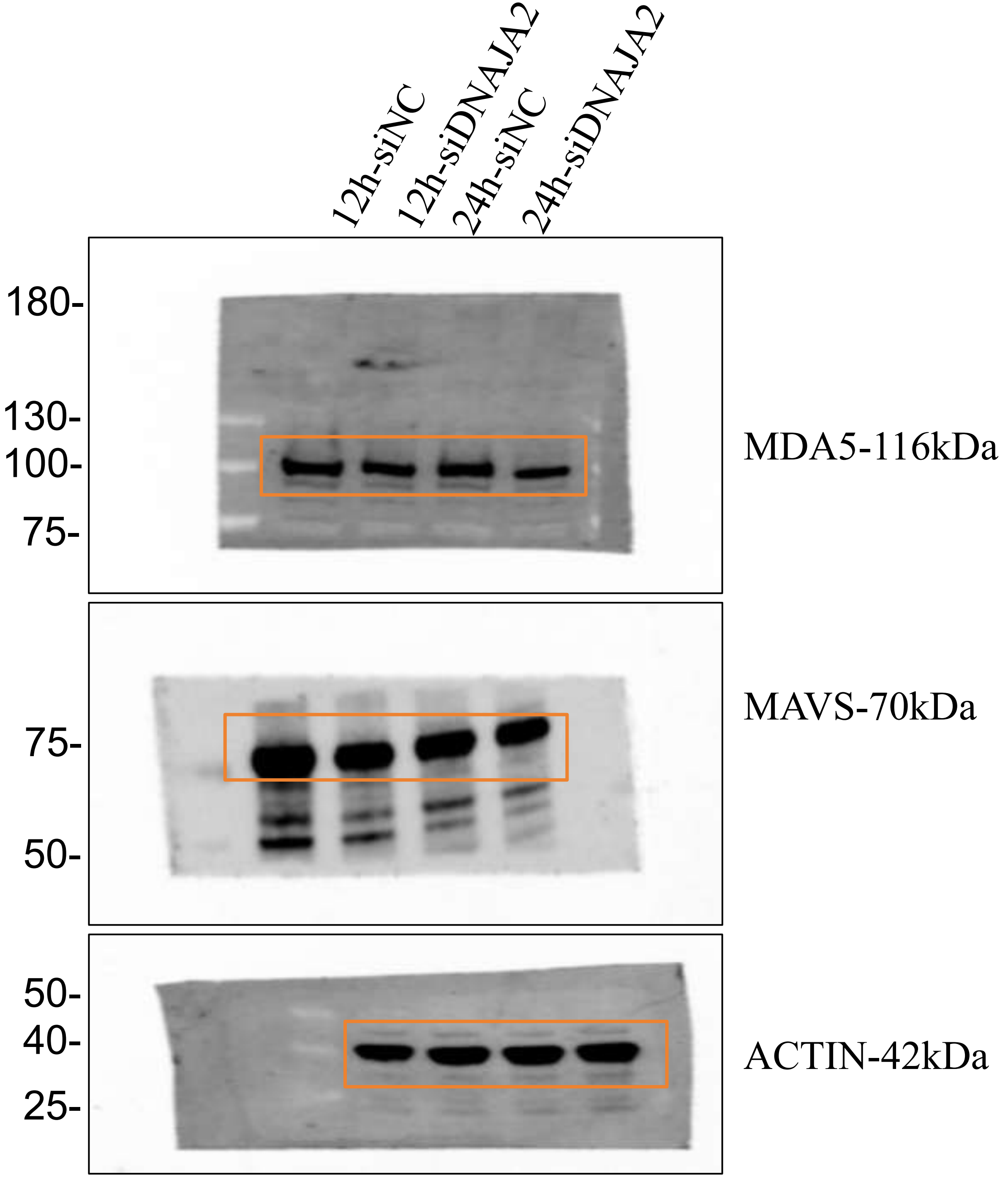

Fig.5G Repeat 2

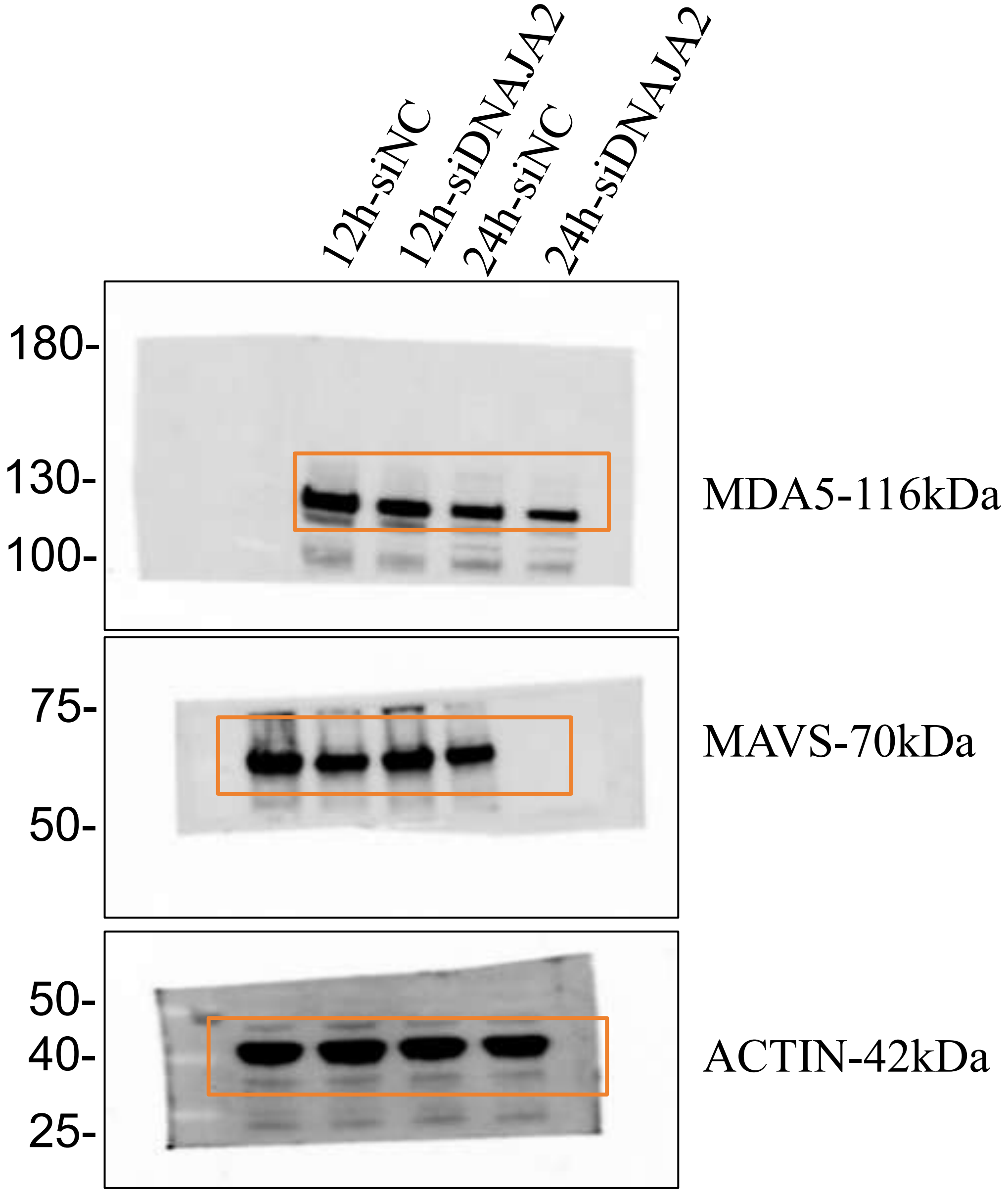

Fig.5H used

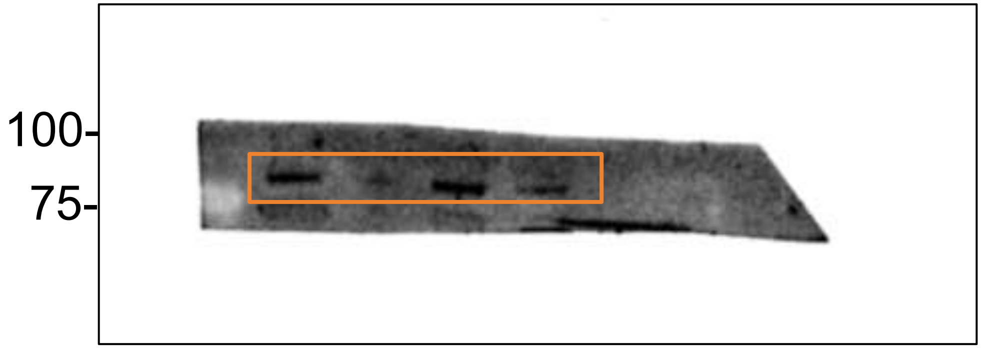

TBK1-86kDa

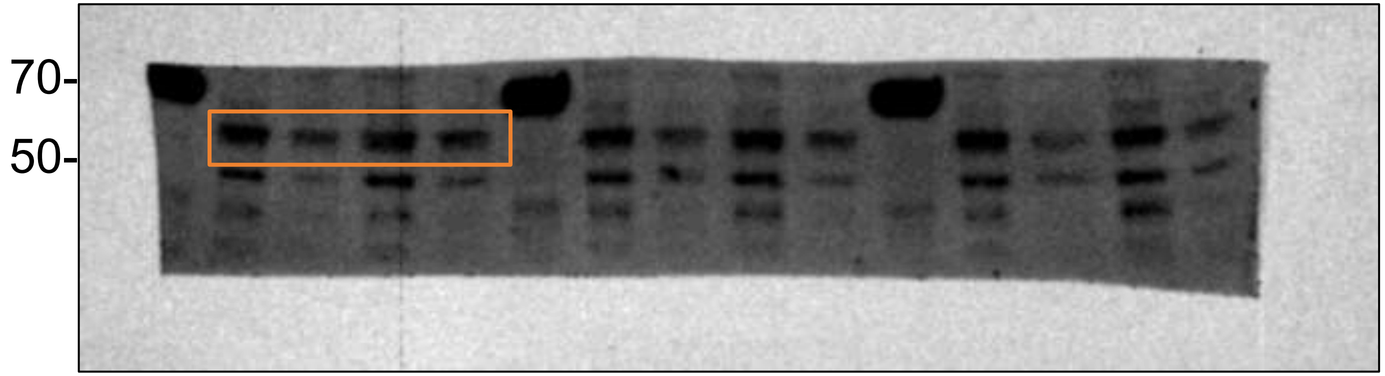

IRF3-56kDa

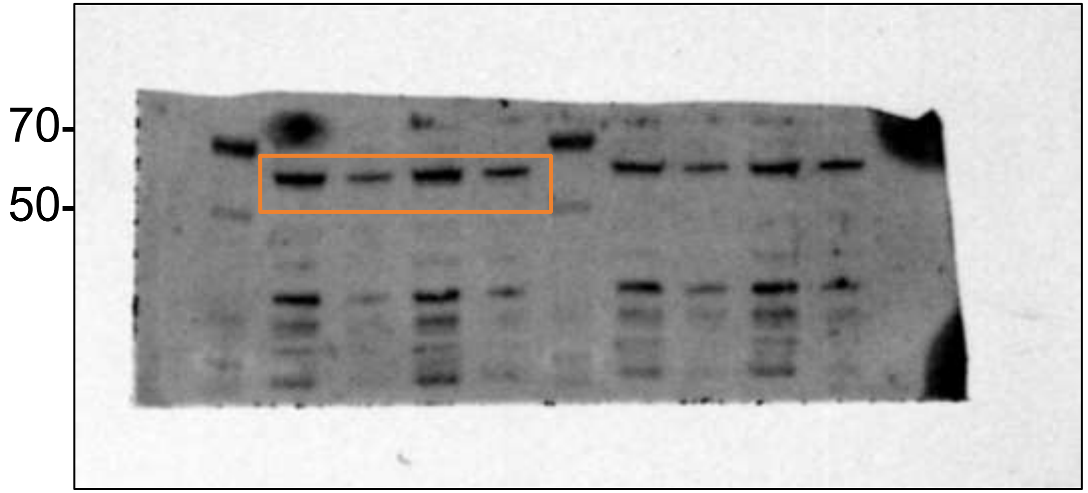

P-IRF3-56kDa

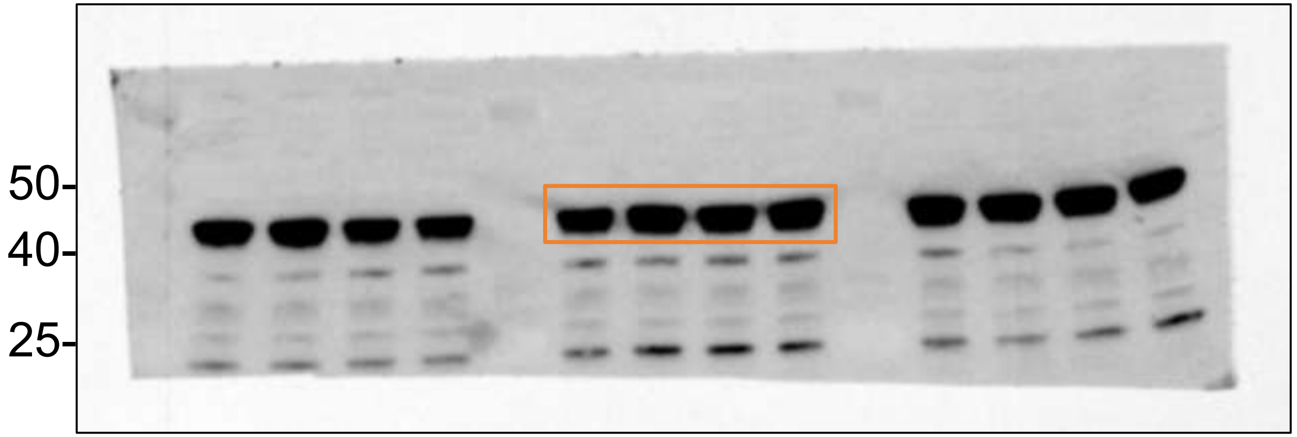

ACTIN-42kDa

Fig.5H repeat

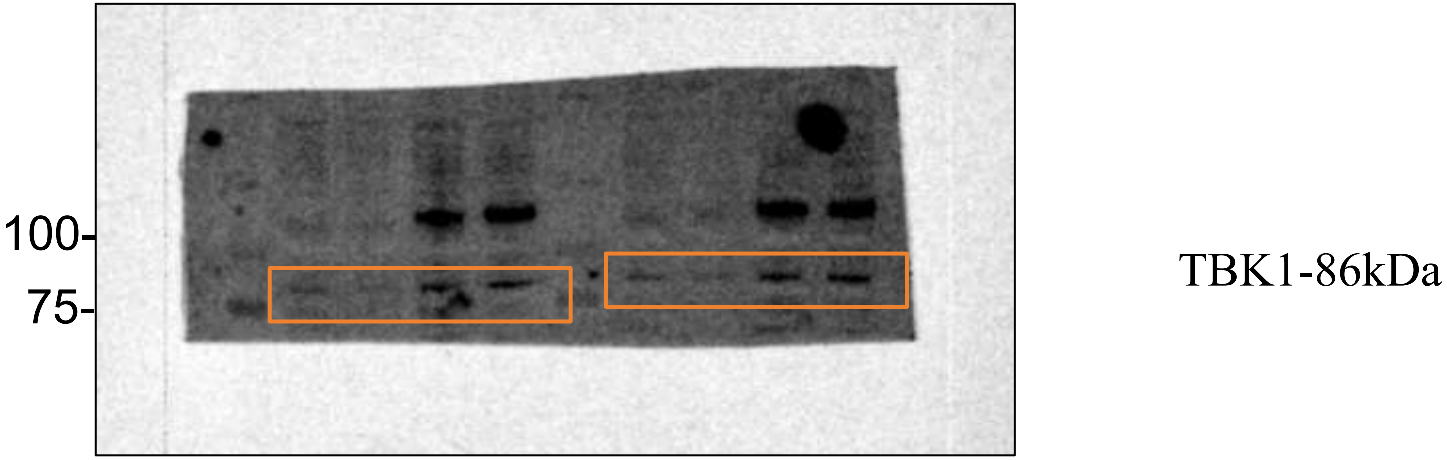

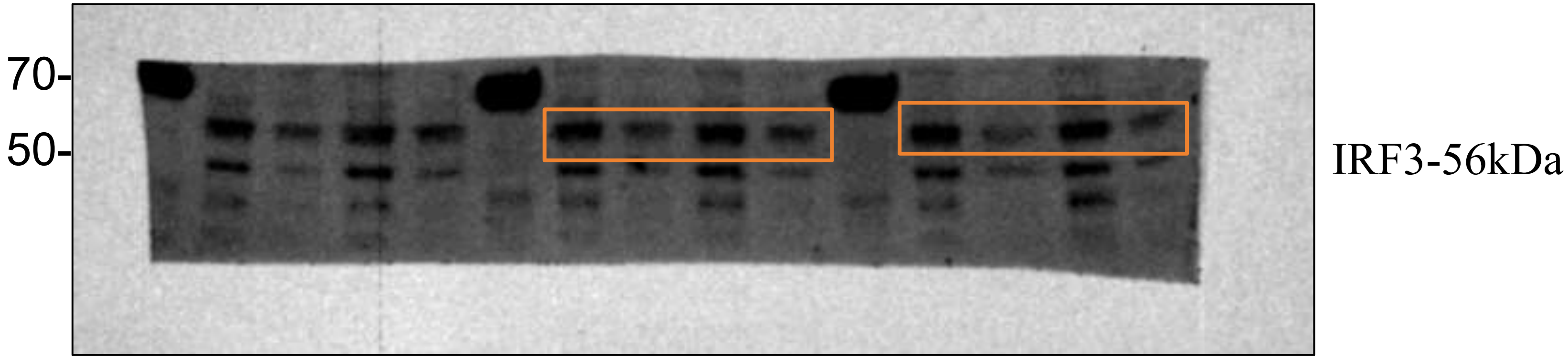

Fig.5H repeat

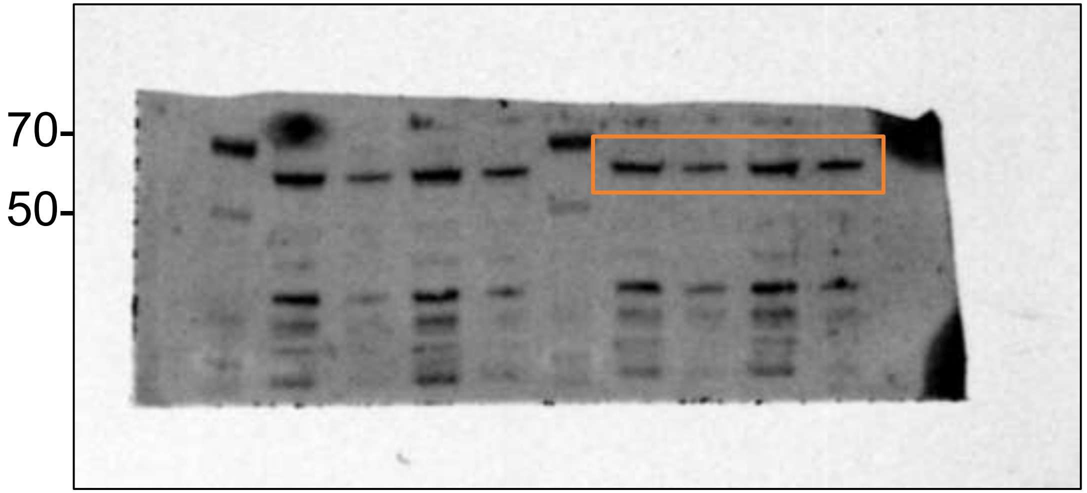

P-IRF3-56kDa

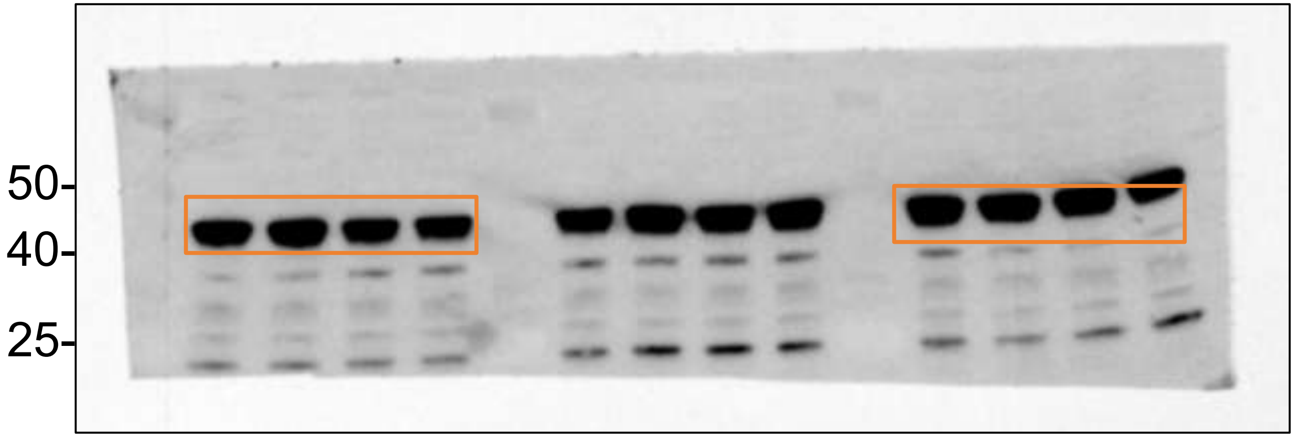

ACTIN-42kDa

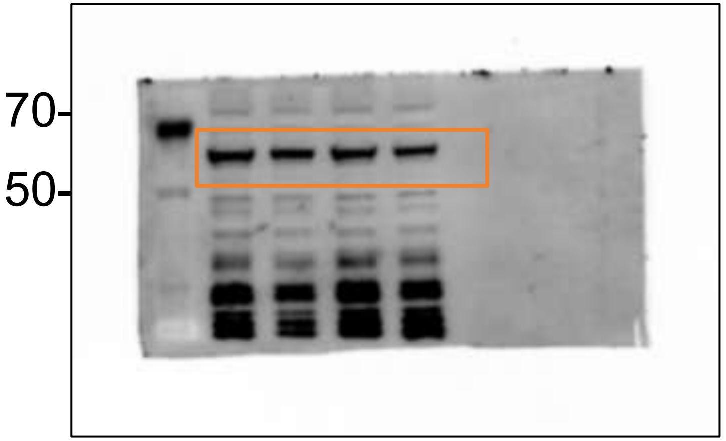

P-IRF3-56kDa

Fig.5K used

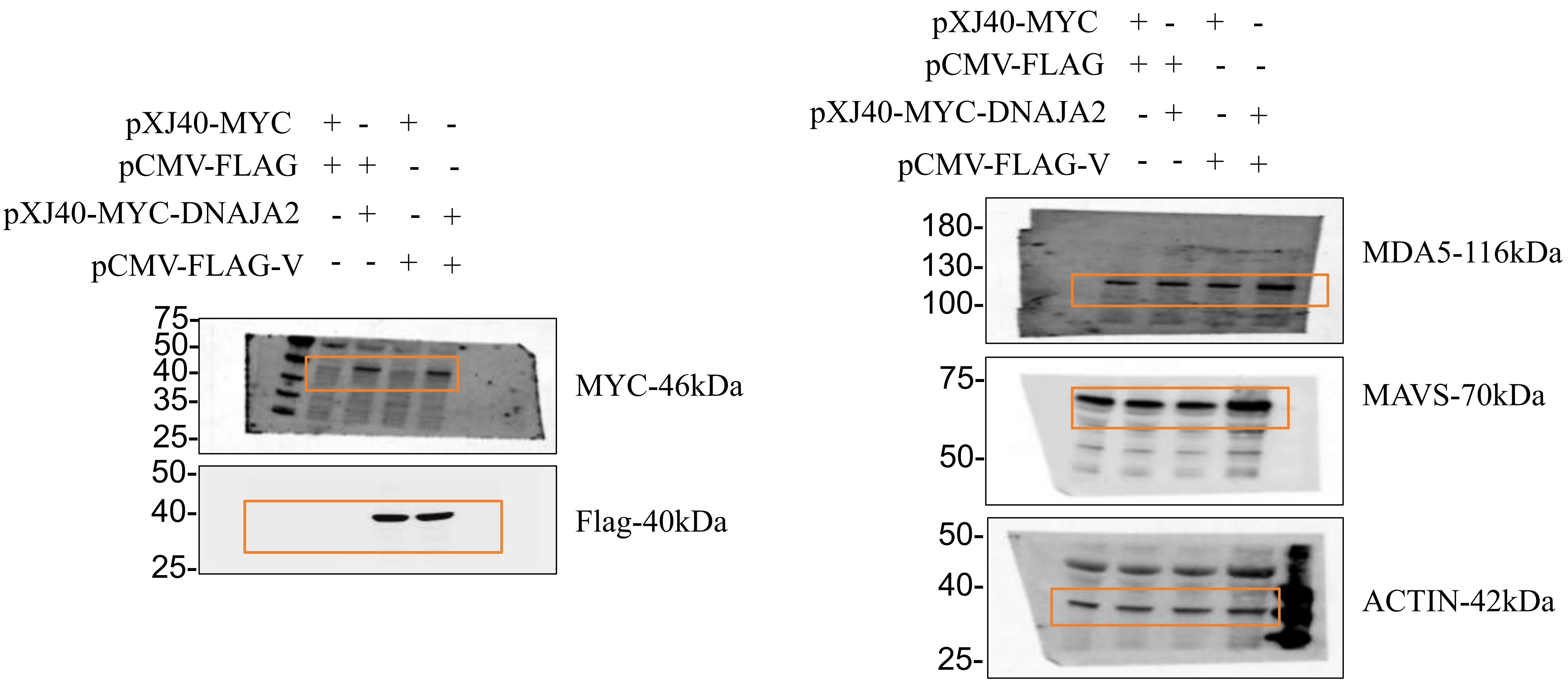

pXJ40-MYC + - + -
pCMV-FLAG + + - -
pXJ40-MYC-DNAJA2 - + - +
pCMV-FLAG-V - - + +
75-
50-
40-
35-
25-
MYC-46kDa
50-
40-
25-
Flag-40kDa
pXJ40-MYC + - + -
pCMV-FLAG + + - -
pXJ40-MYC-DNAJA2 - + - +
pCMV-FLAG-V - - + +
180-
130-
100-
MDA5-116kDa
75-
50-
MAVS-70kDa
50-
40-
25-
ACTIN-42kDa

Fig.5K Repeat 1

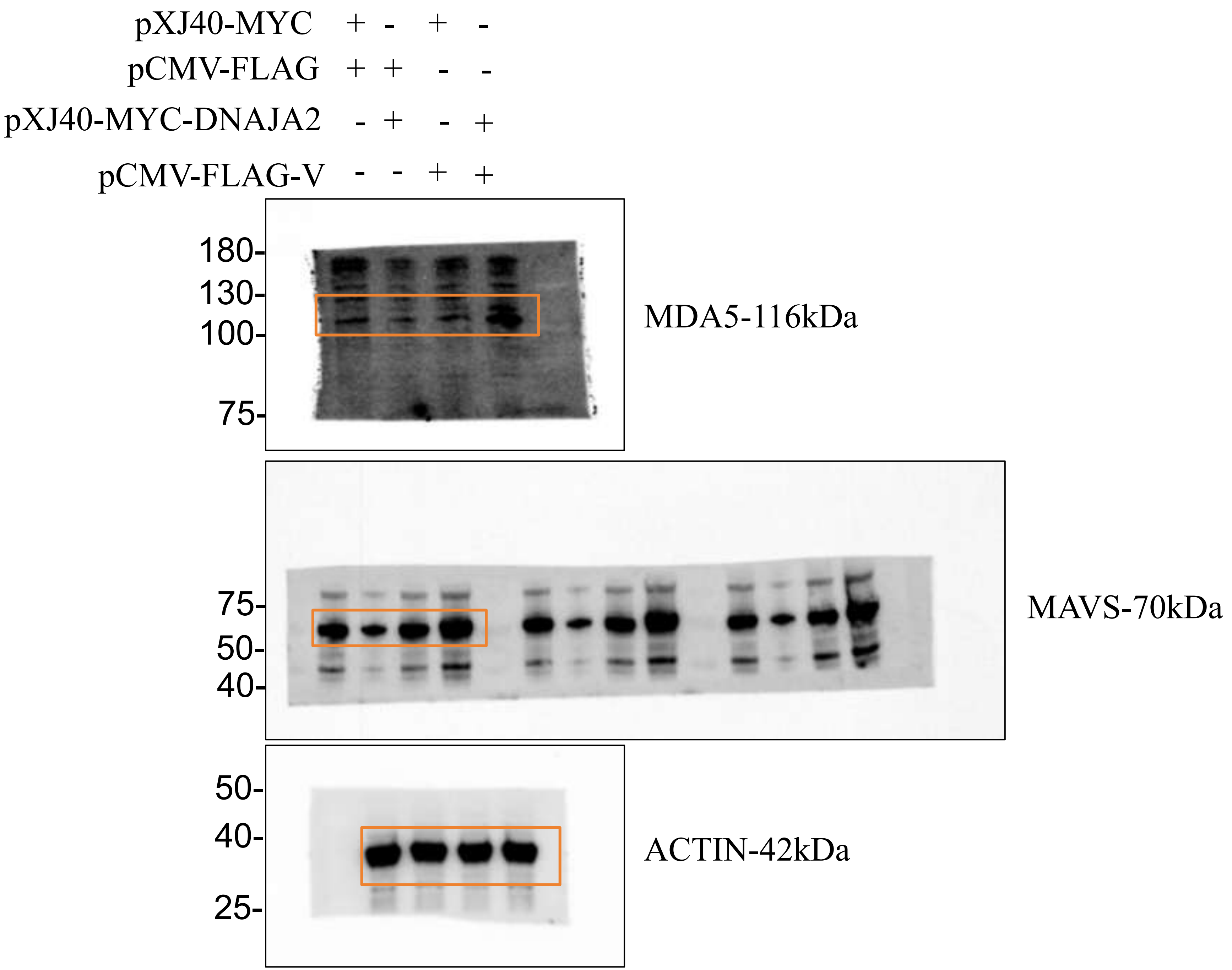

Fig.5K Repeat 2

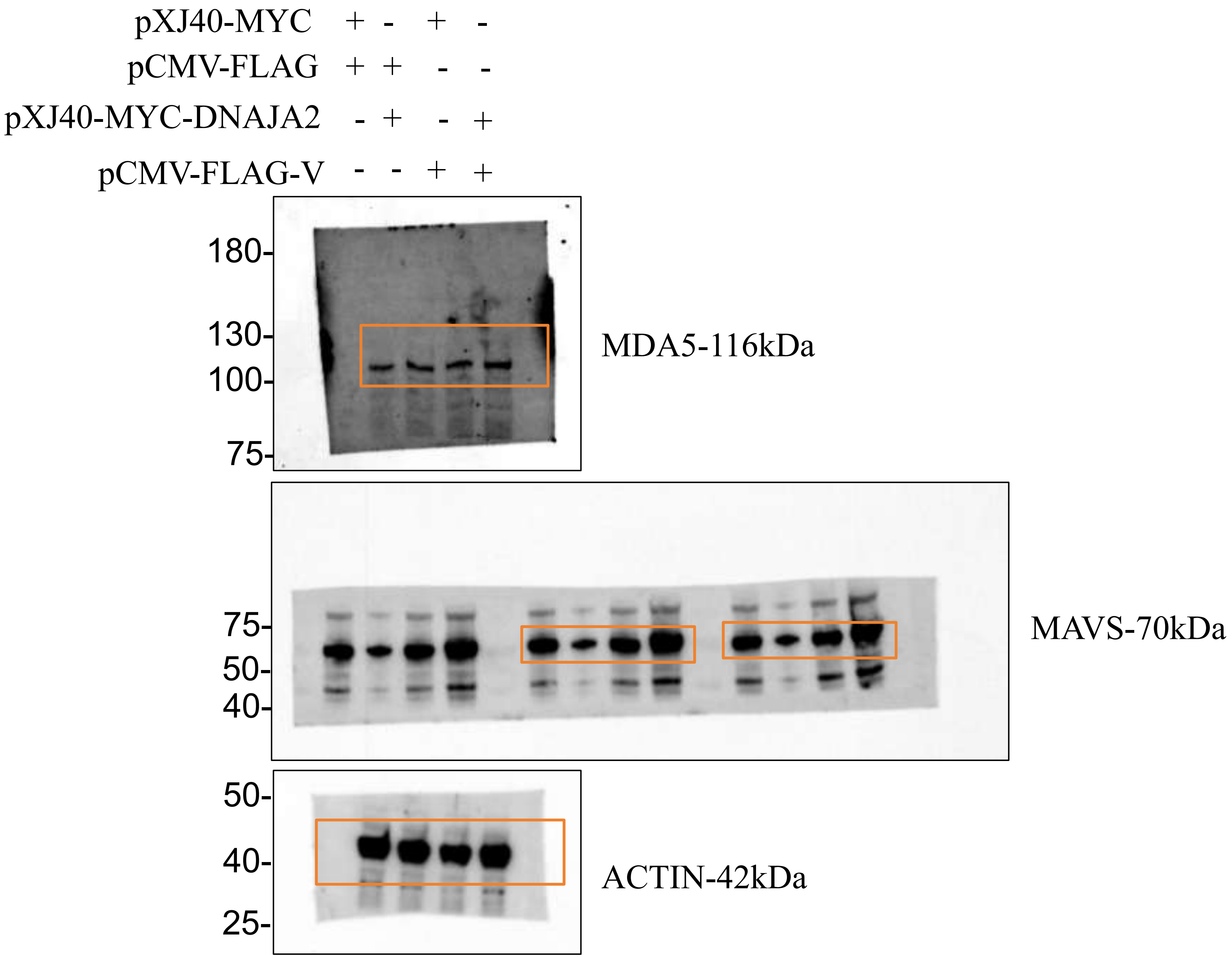

Fig.5L used

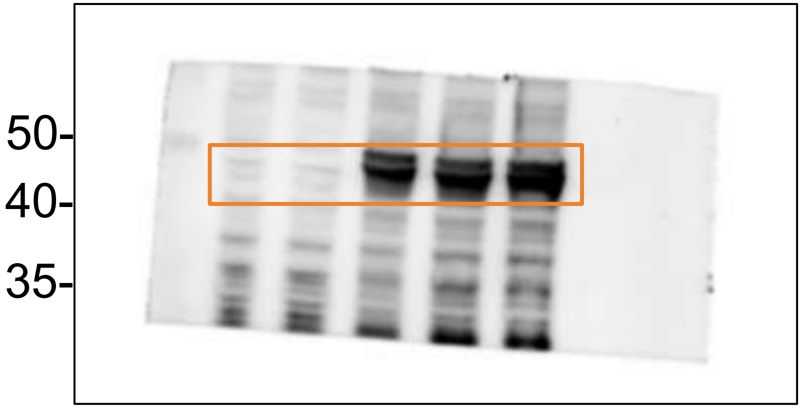

MYC-46kDa

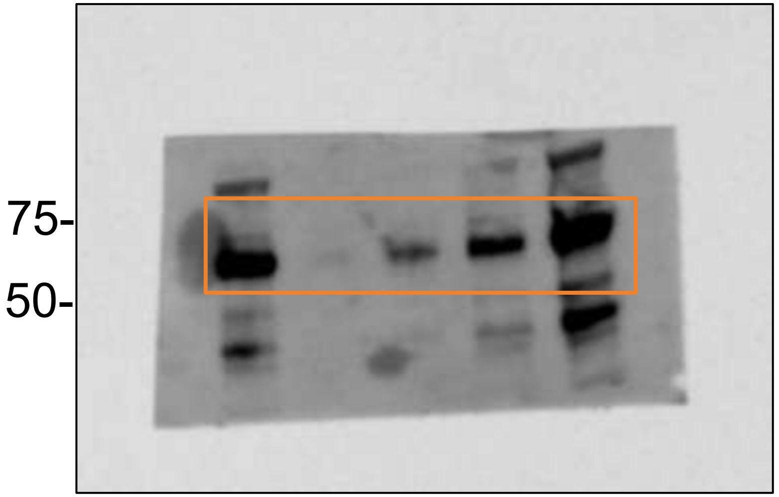

MAVS-70kDa

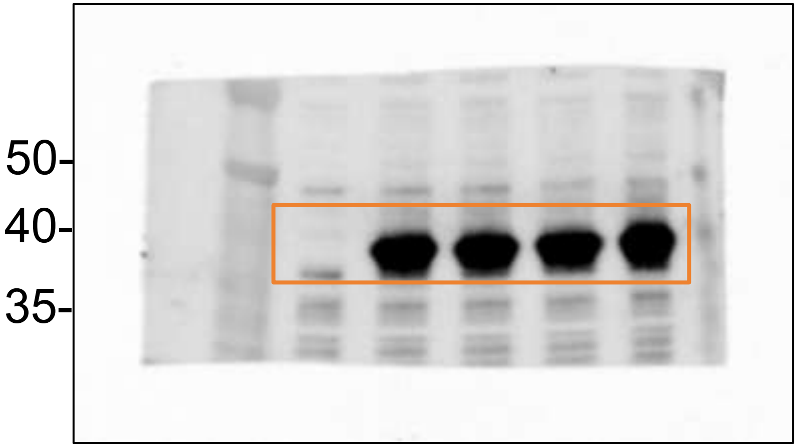

Flag-40kDa

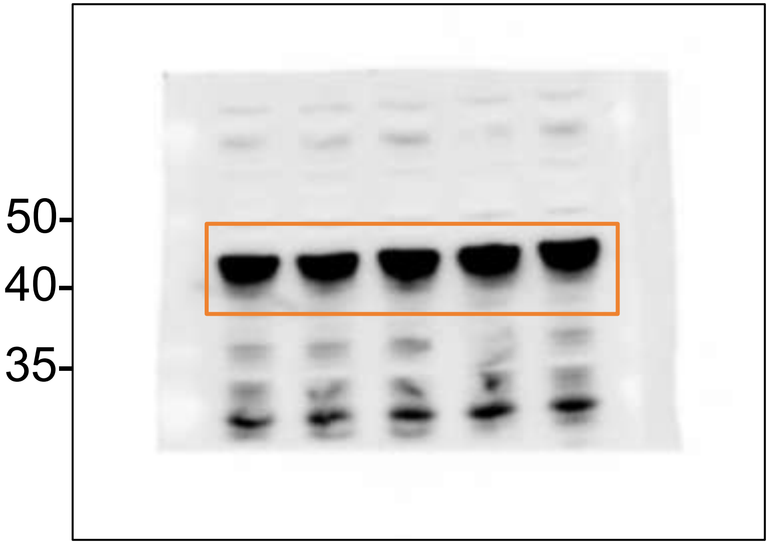

ACTIN-42kDa

Fig.5L Repeat 1

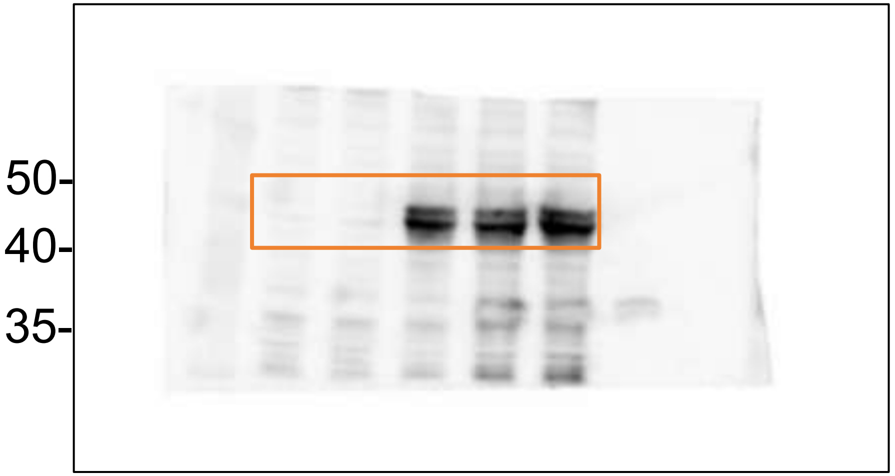

MYC-46kDa

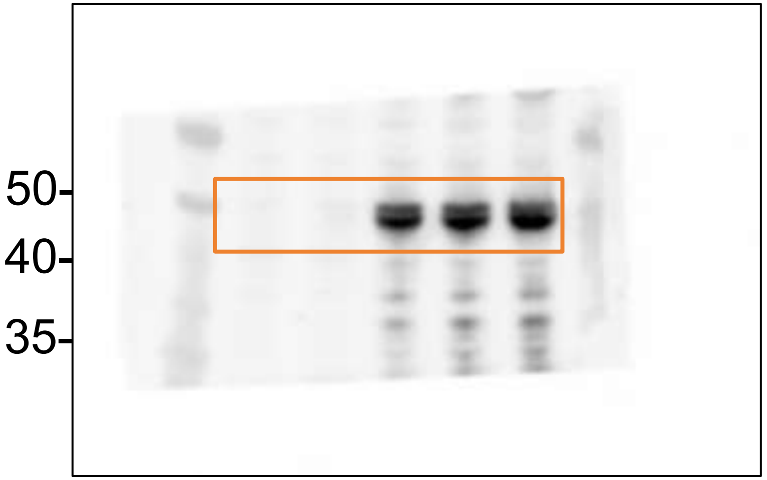

MYC-46kDa

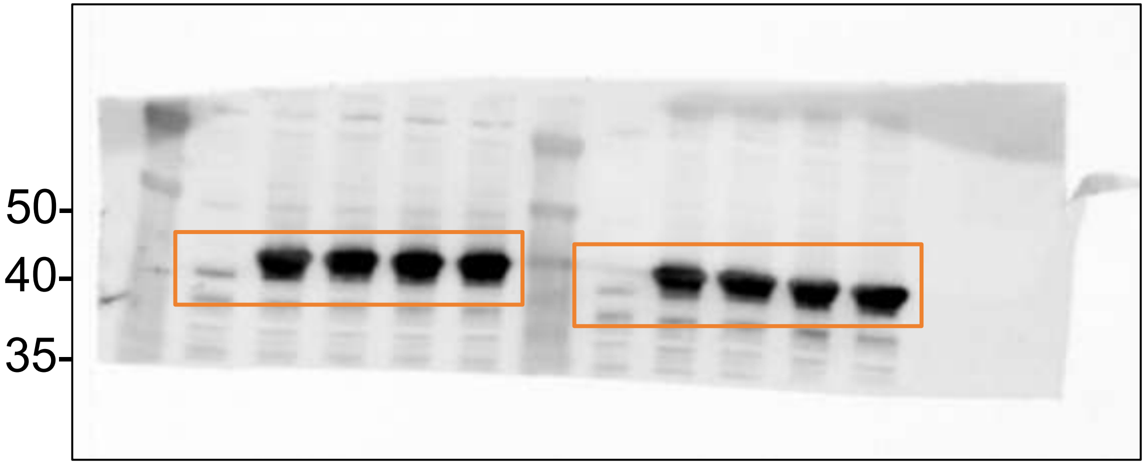

Flag-40kDa

Fig.5L Repeat 1

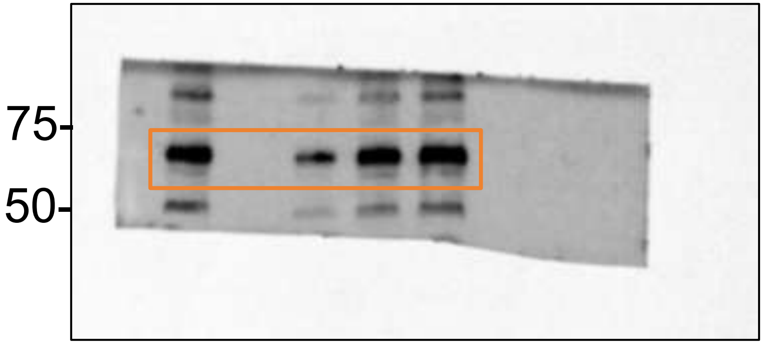

MAVS-70kDa

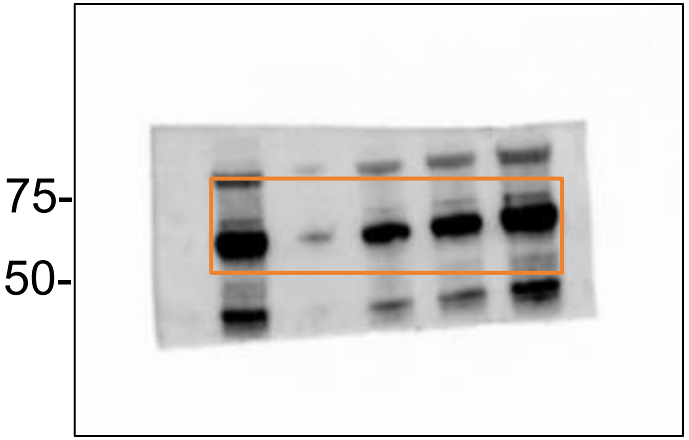

MAVS-70kDa

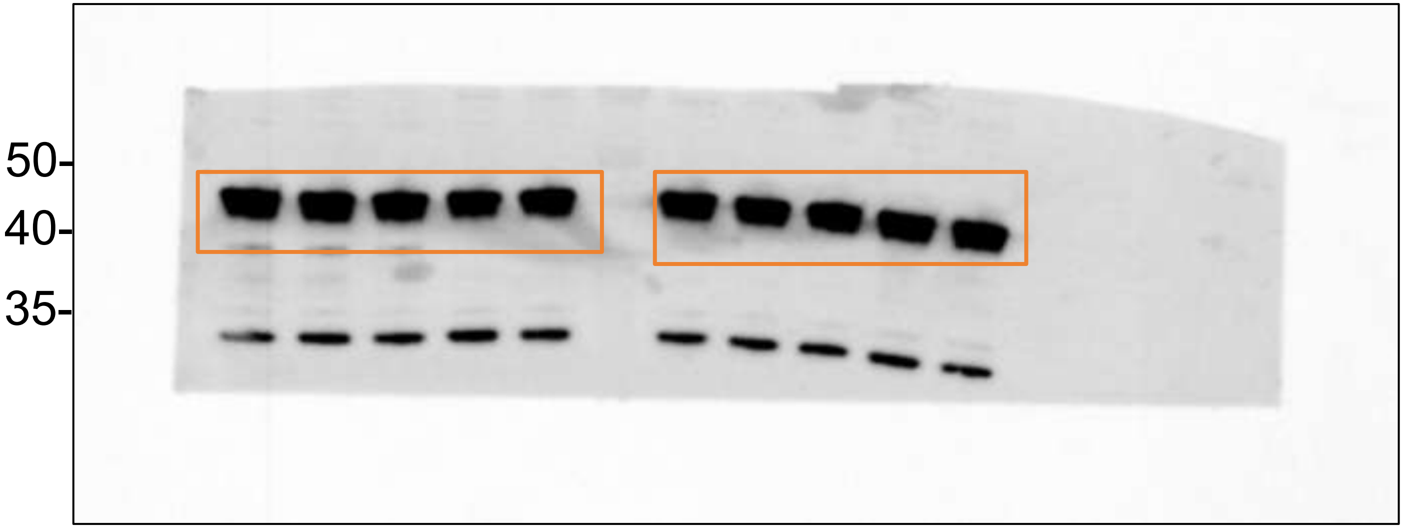

ACTIN-42kDa
